# Xanthine oxidase inhibitors for prevention of cardiovascular events: a systematic review and meta-analysis of randomized controlled trials

**DOI:** 10.1186/s12872-018-0757-9

**Published:** 2018-02-07

**Authors:** Markus Bredemeier, Lediane Moreira Lopes, Matheus Augusto Eisenreich, Sheila Hickmann, Guilherme Kopik Bongiorno, Rui d’Avila, André Luis Bittencourt Morsch, Fernando da Silva Stein, Guilherme Gomes Dias Campos

**Affiliations:** 1 Rheumatology Service at Hospital Nossa Senhora da Conceição, Grupo Hospitalar Conceição, Porto Alegre, RS Brazil; 2 Serviço de Reumatologia do Hospital Nossa Senhora da Conceição, Avenida Francisco Trein, 596, sala 2048, Porto Alegre, RS 91350-200 Brazil

**Keywords:** Gout, Treatment, Cardiovascular disease, Meta-analysis, Xanthine Oxidase inhibitors

## Abstract

**Background:**

Xanthine oxidase inhibitors (XOI), classified as purine-like (allopurinol and oxypurinol) and non-purine (febuxostat and topiroxostat) XOI, present antioxidant properties by reducing the production of reactive oxygen species derived from purine metabolism. Oxidative stress is an important factor related to endothelial dysfunction and ischemia-reperfusion injury, and may be implicated in the pathogenesis of heart failure, hypertension, and ischemic heart disease. However, there is contradictory evidence regarding the possible cardiovascular (CV) protective effect exerted by XOI. Our objective is to compare the incidence of major adverse cardiovascular events (MACE), mortality, total (TCE) and specific CV events in randomized controlled trials (RCTs) testing XOI against placebo or no treatment.

**Methods:**

PubMed, EMBASE, Web of Science, Cochrane Central, Lilacs databases were searched from inception to Dec 30 2016, along with hand searching. RCTs including exclusively adult individuals, lasting ≥ 4 weeks, with no language restriction, were eligible. Independent paired researchers selected studies and extracted data. Considering the expected rarity of events, Peto and DerSimonian/Laird odds ratios (OR), the latter in case of heterogeneity, were used for analysis. Random-effects meta-regression was used to explore heterogeneity.

**Results:**

The analysis of MACE included 81 articles (10,684 patients, 6434 patient-years). XOI did not significantly reduce risk of MACE (OR_P_ = 0.71, 95% CI 0.46–1.09) and death (0.89, 0.59–1.33), but reduced risk of TCE (0.60, 0.44–0.82; serious TCE: 0.64, 0.46 to 0.89), and hypertension (0.54, 0.37 to 0.80). There was protection for MACE in patients with previous ischemic events (0.42, 0.23–0.76). Allopurinol protected for myocardial infarction (0.38, 0.17–0.83), hypertension (0.32, 0.18–0.58), TCE (0.48, 0.31 to 0.75, I^2^ = 55%) and serious TCE (0.56, 0.36 to 0.86, I^2^ = 44%). Meta-regression associated increasing dose of allopurinol with higher risk of TCE and serious TCE (*P* < 0.05). Accordingly, lower doses (≤ 300 mg/day) of allopurinol reduced the risk of TCE, unlike higher doses. Non-purine-like XOI did not significantly reduce or increase the risk of adverse CV events, but confidence intervals were wide. Quality of evidence was generally low to moderate.

**Conclusions:**

Purine-like XOI may reduce the incidence of adverse CV outcomes. However, higher doses of allopurinol (> 300 mg/day) may be associated with loss of CV protection.

**Electronic supplementary material:**

The online version of this article (10.1186/s12872-018-0757-9) contains supplementary material, which is available to authorized users.

## Background

In human cells, the metabolism of purine bases generates hypoxanthine, which is converted to uric acid in a 2-step process catalyzed by xanthine oxidase. During this process, there is production of reactive oxygen species (ROS, H_2_O_2_ and O_2_-), which in excess may reduce the production of nitric oxide, leading to endothelial dysfunction (ED). ED, characterized by vasoconstriction, thrombogenicity due to activation of platelets, and smooth muscle proliferation, is an important step in the promotion of atherosclerosis, thrombosis, and hypertension. ROS may also reduce myocardial contractility, leading to heart failure, and are implicated in ischemia-reperfusion injury [1, 2].

There is evidence suggesting that high levels of uric acid represent an independent cardiovascular risk factor and that the use of xanthine oxidase inhibitors (XOI) may reduce the risk of major adverse cardiovascular events (MACE) [1–5]. XOI are classified as purine- (allopurinol and oxypurinol) and non-purine-like (febuxostat and topiroxostat) depending on their chemical structure. The possible cardiovascular (CV) benefits imputed to XOI may rely on their anti-oxidant properties (by inhibiting the production of ROS released during the activity of xanthine oxidase) and/or the reduction of uric acid levels [1, 2, 6, 7]. Improvement in markers of endothelial dysfunction have been demonstrated in experimental studies testing the CV effects of XOI [6, 8, 9]. However, the clinico-epidemiological evidence of the protective CV action of XOI is based mainly on observational studies [3, 10–12], and the results have not been unanimously positive [13, 14]. Among randomized controlled trials (RCTs), the results are widely variable, with some studies suggesting CV benefits [15–17] and others presenting negative results [18, 19]. When considering the use of XOI beyond it is usual indication in the treatment of gout, the potential harms related to adverse events, which may be serious, must also be considered [20, 21]. At the present moment, asymptomatic hyperuricemia or prevention of CV events are not accepted indications for the use XOI [21, 22].

Considering the doubts on the benefits of XOI for prevention of CV events, in the present study we perform a systematic review and meta-analysis comparing the incidence of MACE, death, and specific CV events in patients enrolled in RCTs where XOI was compared with placebo or no treatment. Our objective was to perform a broad review of the existing literature in order to synthesize the evidence derived from experimental studies in relation to CV effects of XOI.

## Methods

The systematic review was conducted in accordance with the recommendations from the Preferred Reporting Items for Systematic Reviews and Meta-Analyses (PRISMA) statement [23]. The study protocol was registered and published in the International Prospective Register of Systematic Reviews (PROSPERO: CRD42015016073 [24]).

### Study selection criteria

Following the inclusion criteria, RCTs comparing purine-like or non-purine XOI with placebo or no treatment (control) for a period equal or superior to 28 days in adult patients were potentially eligible, with no language restriction. Study eligibility was independent from the clinical condition under treatment, cardiovascular risk profile of the patients, or primary outcomes of the RCTs. Studies presenting 3 or more treatment arms (one arm receiving an alternative medication as colchicine, for example) were included as long as separate data of XOI and control groups could be extracted; however, data from study arms not testing XOI or placebo/no treatment were not considered in the analysis. Studies that followed or treated the patients with XOI for less than 4 weeks, or included individuals with age < 18 years, were excluded. Studies presenting any kind of co-intervention (diet, physical therapy, background or adjuvant medications, or any procedures) differing systematically between intervention (XOI) and control groups were not allowed. However, studies whose background medications or co-interventions did not differ systematically between XOI or control (all patients were on statins, for example) were allowed if no other exclusion criteria were present. Also were excluded studies whose experimental group could receive XOI or non-XOI hypouricemiant (a mixed experimental group) at investigator’s discretion, even if there was a randomization for intervention and control group. RCTs whose phase of follow-up was longer than the period on active treatment were only allowed if such studies permitted to determine the occurrence of adverse events during the on-treatment period, as our objective was to balance the benefits and risks while patients were receiving active therapy with XOI; otherwise, the study was excluded. Non-controlled experimental studies, controlled studies in which the treatment was not randomly allocated (quasi-experimental), or observational studies were not allowed. RCTs considered to be fraudulent, as well as those with inadequate, incomplete, or absent report of adverse events were also excluded. Cross-over studies in which the occurrence of adverse events could not be ascertained to the first period (i.e., before crossing over) were excluded to avoid the potential of carry-over effect confounding the analysis.

### Data sources and searches

Major electronic databases (PubMed, EMBASE, Web of Science, Cochrane Library, and Lilacs) were searched for published literature from their inception to September 29, 2014. The literature search (see Supplementary Texts 1, 2, 3 and 4 of Additional file 1 for search terms) was updated to January 11, 2016 and again to Dec 30, 2016. An extensive hand search (including a site indexing Chinese articles) was begun in July 9, 2016 and also was updated to December 30, 2016. No language restriction was applied. See further details in Additional file 1: Supplementary text 5. Missing or unpublished data were sought by trying to contact authors or sponsors via e-mail and the ResearchGate™ site; repeated messages were sent in case of no response.

### Study selection

The references obtained using the described search strategy were evaluated by 2 independent investigators. The initial evaluation was based on title and abstract; the articles selected by at least one of the investigators were obtained in electronic or printed full-text format and reevaluated for inclusion by 2 independent investigators. The final decision for inclusion was made by consensus or discussion with a third observer in case of divergence.

### Data extraction and quality assessment

Data on study features and outcomes were extracted independently by 2 trained researchers using a specifically designed protocol. The articles were evaluated for risk of bias using the method described in the Cochrane handbook [25] by 2 independent reviewers. Divergences were resolved by consensus or discussion involving another researcher. All data extraction and database typing were reviewed by the principal investigator (MB) before the final analysis, and doubts were resolved in consensus. For further details, see Supplementary Text 5 in Additional file 1.

### Data synthesis and analysis

Aggregate data extracted from RCTs were analyzed using the modified intention-to-treat (considering patients who received at least one dose of the allocated treatment) or, alternatively, intention-to-treat results. If that was not possible, available case analysis was employed. Statistical analysis was made using OpenMetaAnalyst for Windows Version 8.0 (Center for Evidence-based Medicine, Brown University, Providence, RI, USA) and Meta and Metafor packages of R version 3.3.3 (R Foundation for Statistical Computing, Vienna, Austria); IBM SPSS Statistics for Windows, Version 20.0 (IBM Corporation, Armonk, NY, USA) was used for ancillary analyses.

The primary outcomes were the incidence of major adverse cardiovascular outcomes (MACE: cardiovascular death, non-fatal myocardial infarction, unstable angina requiring urgent revascularization, or non-fatal stroke) and death. The secondary outcomes were cardiovascular death, acute ischemic heart disease (myocardial infarction or unstable angina requiring revascularization), stroke, new or worsening hypertension (including hypertensive crisis), heart failure or worsening heart failure (including acute pulmonary edema), cardiac arrhythmias, and total adverse cardiovascular events (TCE; any of the above plus venous and arterial visceral or peripheral thrombotic events), and serious adverse events (those requiring urgent medical procedures and/or hospitalization, life-threatening or leading to death). We created the variable serious CV events during the phase of analysis considering that the variable total CV events included events that did not threaten patients’ life (mild elevations of blood pressure, mild worsening heart failure, or non-serious arrhythmias, for example). If one or more outcomes could not be extracted from a study, this study was removed only from the analysis involving these outcomes.

The statistical analysis was planned considering the expected rarity of adverse CV events in most studies (i.e., meta-analysis of rare events) [26, 27]. The results were described in terms of odds-ratios (OR) along with 95% confidence intervals. Associations were analyzed using Peto odds ratio (OR_P_) without zero-cell corrections. Heterogeneity was evaluated using Cochran’s Q test and I-squared (I^2^) statistics and considered present when the Cochran’s test showed *P* ≤ 0.10 or the I-squared statistic was ≥ 40%. DerSimonian and Laird random effects model with continuity correction was used to account for heterogeneity. Random effects uni- and multivariate meta-regression analyses including some specific independent variables (such as duration of study, age, and dosage of allopurinol) were planned to explore heterogeneity; maximum likelihood (ML) was the method used to estimate the between-study variance tau-squared, and the amount of heterogeneity accounted for by the independent variables was estimated by the R^2^ statistic. As a sensitivity analysis, meta-regression was also performed using restricted maximum likelihood (REML) with Hartung and Knapp (hakn) method to adjust test statistics. Meta-regression analyses were performed excluding studies with both arms zero events (BA0E), except if indicated otherwise. *P* values less than or equal to 0.05 were considered statistically significant. Sensitivity analyses were conducted to account for risk of bias. Publication bias was assessed using funnel plots and Egger’s test; trim and fill method was used to compensate for publication bias. Subgroup analyses were planned for patients with and without cardiovascular risk factors or diagnosis of established diseases.

## Results

The search procedures are described in Additional file 1: Figures S1 to S3. In total, 12,273 records were screened, 434 were assessed for eligibility, and 91 RCTs had at least one outcome of interest that could be analyzed. The analysis of MACE included 81 articles (10,684 patients, 6434 patient-years), and death, 90 articles (11,861 patients, 7571 patient-years). Additional file 2 summarizes the features of the studies included in the meta-analysis, and Additional file 3 describes the list of potentially relevant studies that were excluded in the phase of analysis of full-text articles. Most included studies (79%) selected predominantly individuals presenting at least one risk factor for cardiovascular events (gout/hyperuricemia, hypertension, older age, renal dysfunction, diabetes, smoking, dislipidemia, previous CV events or established CV disease, or obesity). The evaluation of risk of bias is described in Additional file 1: Table S1; 20 studies were at low risk of bias, but most studies were at unknown (41) or high risk (30) of bias. The mean (SD) and median duration of follow-up were, respectively, 198 (224) and 90 days (percentiles 25th, 75th: 60, 270 days; range 28 to 1095 days).

**Fig. 1:**
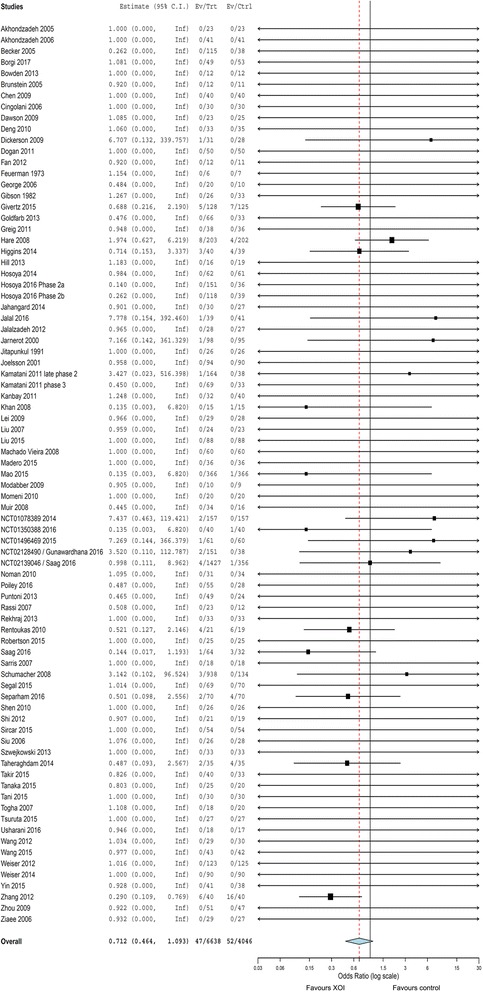
Forest plot analysis comparing the risk of major adverse cardiovascular events (MACE) between xanthine oxidase inhibitors and control. Numbers are Peto odds ratio and 95% CI. Heterogeneity: I^2^ = 10%; Cochrans’s Q test, *P* = 0.324

The results for the primary outcomes are shown in Fig. 1 (only for MACE) and Table 1. The use of XOI was not significantly associated with the risk of MACE (OR_P_ = 0.71, 95% CI 0.46 to 1.09) or death (0.89, 0.59 to 1.33; Additional file 1: Figure S4) in the entire sample. Excluding studies where most individuals did not present CV risk factors, there was a trend for protection for MACE (0.67, 0.44 to 1.04, *P* = 0.074, I^2^ = 8% [Cochran’s Q test, *P* = 0.353], 63 studies). Subgroup analysis showed reduced risk of MACE in individuals who presented acute ischemic encephalic or coronary events (0.42, 0.23 to 0.76, *P* = 0.004, I^2^ = 0% [*P* = 0.914], 9 studies; Additional file 1: Figure S5), suggesting benefit of XOI in secondary prevention.

**Table 1:** Results for primary and secondary outcomes

Outcomes	All studies with extractable data
	OR_P_ (95% CI), *P* value, I^2^ (*P* value), number of studies
Primary outcomes	
Major adverse cardiovascular events (MACE)	0.71 (0.46 to 1.09), *P* = 0.120, I^2^ = 10% (*P* = 0.324), 81 studies
Death	0.89 (0.59 to 1.33), *P* = 0.573, I^2^ = 0% (*P* = 0.704), 90 studies
Secondary outcomes	
Myocardial infarction or urgent revascularization procedure	0.56 (0.28 to 1.11), *P* = 0.096, I^2^ = 5% (*P* = 0.395), 78 studies
Stroke	0.65 (0.20 to 2.19), *P* = 0.490, I^2^ = 0% (*P* = 0.486), 76 studies
Cardiovascular death	0.82 (0.49 to 1.38), *P* = 0.457, I^2^ = 0% (*P* = 0.725), 82 studies
New/worsening hypertension	0.54 (0.37 to 0.80), *P* = 0.002, I^2^ = 0% (*P* = 0.494), 71 studies
New/worsening heart failure	0.94 (0.68 to 1.28), *P* = 0.670, I^2^ = 55% (*P* = 0.023), 75 studies
Arrhythmias	0.94 (0.33 to 2.67), *P* = 0.909, I^2^ = 0% (*P* = 0.539), 71 studies
Total cardiovascular events	0.66 (0.54 to 0.80), *P* < 0.001, I^2^ = 49% (*P* = 0.002), 81 studies; D-L: 0.60 (0.44 to 0.82), *P* = 0.001, I^2^ = 41% (*P* = 0.012)
Serious cardiovascular events	0.64 (0.51 to 0.81), *P* < 0.001, I^2^ = 34% (*P* = 0.050), 81 studies; D-L: 0.64 (0.46 to 0.89), *P* = 0.008, I^2^ = 24% (*P* = 0.135)
Serious adverse events	0.93 (0.76 to 1.14), *P* = 0.488, I^2^ = 11% (*P* = 0.270), 81 studies

*OR*_*P*_ Peto odds ratio, except when indicted otherwise, *CI* confidence interval, *I*^*2*^ statistic of heterogeneity (P value of Cochran’s Q test), *D-L* DerSimonian and Laird random effects odds ratio with zero-cell continuity correction

The results for secondary outcomes of XOI versus control are shown in Table 1 and Additional file 1: Figures S6 to S10. There was a significant reduction in the incidence of new/worsening hypertension, total cardiovascular events, and serious CV events. In subgroup analysis, serious CV events were more strongly reduced in studies including only patients with previous ischemic events (0.36, 0.20 to 0.63, *P* < 0.001, I^2^ = 0% [*P* = 0.978], 9 studies).

In a pre-specified secondary analysis (Table 2), we tested separately the effects of purine-like XOI (allopurinol and oxypurinol) and non-purine like XOI (febuxostat and topiroxostat). Studies testing non-purine like XOI demonstrated no statistically significant cardiovascular protective effect, but confidence intervals were wide. Statistically significant reductions in the risk of myocardial infarction/urgent revascularization procedure, hypertension, total CV events, and serious CV events were observed in the purine XOI subgroup (see also Additional file 1: Figures S11 to S15). Excluding studies where most individuals did not present CV risk factors, there was a significant protection for MACE (0.61, 0.38 to 0.98, *P* = 0.042, I^2^ = 3% [*P* = 0.413], 47 studies) with the use of purine-like XOI.

**Table 2:** The results for primary and secondary outcomes analyzed separately for purine- and non-purine-like xanthine oxidase inhibitors (XOI)

Outcomes	Purine-like XOI (allopurinol or oxypurinol)	Non-purine like XOI (febuxostat or topiroxostat)
	OR_P_ (95% CI), *P* value, I^2^ (P value), number of studies	OR_P_ (95% CI), P value, I^2^ (P value), number of studies
Primary outcomes		
Major adverse cardiovascular events	0.65 (0.41 to 1.05), *P* = 0.076, I^2^ = 9% (*P* = 0.354), 65 studies	1.13 (0.40 to 3.19), *P* = 0.824, I^2^ = 18% (0.290), 19 studies
Death	0.94 (0.62 to 1.44), *P* = 0.785, I^2^ = 0% (*P* = 0.525), 74 studies	0.71 (0.15 to 3.40), *P* = 0.671, I^2^ = 0%, (*P* = 0.956), 19 studies
Secondary outcomes		
Myocardial infarction or urgent revascularization procedure	0.38 (0.17 to 0.83), *P* = 0.015, I^2^ = 0% (*P* = 0.700), 62 studies	2.76 (0.62 to 12.35), *P* = 0.185, I^2^ = 0% (*P* = 0.502), 19 studies
Stroke	0.73 (0.16 to 3.29), *P* = 0.678, I^2^ = 2% (*P* = 0.361), 60 studies	0.54 (0.07 to 4.07), *P* = 0.551, I^2^ = 11% (*P* = 0.337), 19 studies
Cardiovascular death	0.86 (0.50 to 1.46), *P* = 0.570, I^2^ = 0% (*P* = 0.587), 66 studies	0.45 (0.06 to 3.48), *P* = 0.445, I^2^ = 0% (*P* = 0.789), 19 studies
New/worsening hypertension	0.32 (0.18 to 0.58), *P* < 0.001, I^2^ = 0% (*P* = 0.737), 55 studies	0.70 (0.43 to 1.12), *P* = 0.136, I^2^ = 13% (*P* = 0.329), 19 studies
New/worsening heart failure	0.90 (0.66 to 1.24), *P* = 0.539, I^2^ = 72% (*P* = 0.006), 59 studies	1.79 (0.43 to 7.49), *P* = 0.423, I^2^ = 0% (*P* = 0.451), 18 studies
Arrhythmias	1.95 (0.20 to 18.72), *P* = 0.564, I^2^ = 25%, (*P* = 0.264), 54 studies	0.80 (0.25 to 2.56), *P* = 0.708, I^2^ = 0% (*P* = 0.681), 19 studies
Total cardiovascular events	0.57 (0.46 to 0.72), *P* < 0.001, I^2^ = 60% (*P* < 0.001), 65 studies; D-L: 0.48 (0.31 to 0.75), *P* = 0.001, I^2^ = 55% (P = 0.001)	0.90 (0.62 to 1.30), *P* = 0.562, I^2^ = 0% (*P* = 0.734), 19 studies
Serious cardiovascular events	0.59 (0.46 to 0.76), *P* < 0.001, I^2^ = 50% (*P* = 0.011), 65 studies; D-L: 0.56 (0.36 to 0.86), *P* = 0.009, I^2^ = 44% (*P* = 0.028)	1.04 (0.58 to 1.87), *P* = 0.901, I^2^ = 0% (*P* = 0.967), 19 studies
Serious adverse events	0.88 (0.70 to 1.10), *P* = 0.280, I^2^ = 18% (*P* = 0.176), 64 studies	1.12 (0.75 to 1.66), *P* = 0.587, 0% (*P* = 0.679), 19 studies

*OR*_*P*_ Peto odds ratio, except when indicted otherwise, *CI* confidence interval, *I*^*2*^ statistic of heterogeneity (P value of Cochran’s Q test), *D-L* DerSimonian and Laird random effects odds ratio with zero-cell continuity correction

**Fig. 2:**
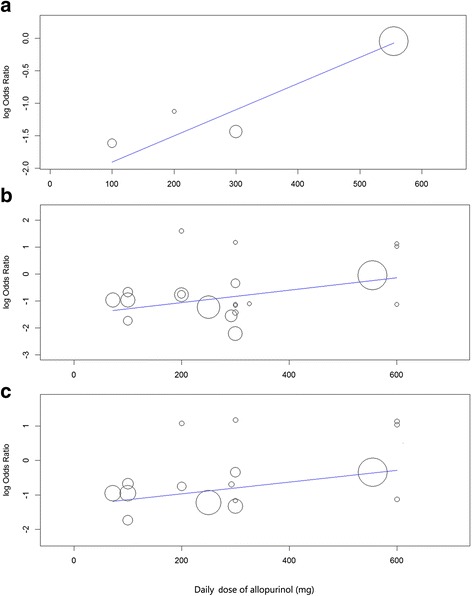
Meta-regression of dose of allopurinol* versus log odds ratio of heart failure (**a**)^a^, total cardiovascular events (**b**)^b^, and serious cardiovascular events (**c**)^c^. * One oxypurinol study [19] contributing with events was excluded from metaregression due to the absence of direct comparisons of serum oxypurinol levels between oral preparations of allopurinol and oxypurinol (produced by Cardiome Pharma Corporation, Vancouver, British Columbia, Canada) used continuously in daily doses. ^a^Meta-regression coefficient = 0.004, 95% confidence interval: 0.001 to 0.008, *P* = 0.008. ^b^0.002, 0.001 to 0.004, *P* = 0.006. ^c^ 0.002, 0.000 to 0.003, *P* = 0.039

There was significant heterogeneity in the analysis of heart failure, total CV events, and serious CV events related to the use of purine-like XOI (Table 2). To explore this heterogeneity, we performed random effects meta-regression analysis of mean age of participants, duration of study, and dose of allopurinol versus risk of the outcomes listed above. There were no significant associations of age of participants and duration of study with these outcomes (*P* > 0.050), although a trend for association of longer duration of study with protection for serious CV events (*P* = 0.052) and of higher age with loss of protection for new/worsening heart failure (*P* = 0.096) were observed. Increasing dose of allopurinol (oxypurinol studies were excluded from this analysis) was positively associated with increasing risk of heart failure (R^2^ = 100.0%, *P* = 0.008), total CV events (R^2^ = 90.4%, *P* = 0.006), and serious CV events (R^2^ = 100.0%, *P* = 0.039) (Fig. 2). Making use of multivariate meta-regression models, there was still a significant association of dose with risk total CV events (*P* = 0.007; controlled for age and study duration) and a trend for association of dose with risk of heart failure (*P* = 0.069; this analysis was adjusted only for age due to excessive number of parameters). Controlling the association of dose with risk of serious CV events for age and duration of study, the statistically significant association observed in univariate analysis (*P* = 0.039) was lost (multivariate *P* = 0.152). However, in this latter multivariate analysis and in that relative to dose versus risk of heart failure (controlled for age), there was a worsening in Omnibus *P* values in comparison to univariate analyses (from to 0.039 to 0.171 and from 0.008 to 0.022, respectively), indicating that the univariate models of dose versus effect probably better fit the data. Despite the absence of heterogeneity, we also tested the association of higher allopurinol dose with serious adverse events, and a trend for positive association was observed (*P* = 0.073). Still using the same statistical methods of meta-regression (ML to estimate the between-study variance tau-squared), but including studies with BA0E, the univariate results for dose versus total CV events (*P* = 0.005), serious CV events (*P* = 0.052), and serious adverse events (*P* = 0.098) were in general maintained. However, the analysis of dose versus risk of heart failure lost statistical significance (*P* = 0.162) possibly due to dilution of effect caused by the large number of studies with zero events in both arms.

The results of meta-regression of dose of allopurinol versus effect presented in the previous paragraph were tested using alternative statistical methods. Using REML with hakn method, there were still significant univariate associations of dose with higher risk of heart failure (*P* = 0.035), total CV events (*P* = 0.045), and serious CV events (*P* = 0.018), with a trend for associations with serious adverse events (*P* = 0.052). Using risk ratio as measure of association (instead of odds ratio, used in previous analyses) and REML with hakn adjustment, the results for all tests described in the last phrase were statistically significant (*P* = 0.010, *P* = 0.004, *P* = 0.042, and *P* = 0.027, respectively).

Considering the positive associations of dose of allopurinol and higher cardiovascular risk, we performed subgroup analysis separating the dose in low (< 300 mg/day), standard (300 mg/day), and high (> 300 mg/day). This analysis suggested protection for total CV events in low- (OR_P_ = 0.39, 0.27 to 0.55, *P* < 0.001, I^2^ = 0% [Cochran’s Q test, *P* = 0.570]) and standard-dose (0.22, 0.12 to 0.39, *P* < 0.001, I^2^ = 10% [*P* = 0.351]) groups, while there was no significant protection with high-dose (0.96, 0.60 to 1.55, *P* = 0.862, I^2^ = 0% [*P* = 0.421]). The results were similar for serious cardiovascular events (low-dose: 0.37, 0.25 to 0.53, *P* < 0.001, I^2^ = 0% [*P* = 0.766]; standard-dose: 0.41, 0.19 to 0.88, *P* = 0.022, I^2^ = 0% [*P* = 0.484]; high-dose: 0.75, 0.44 to 1.27, *P* = 0.285, I^2^ = 8% [*P* = 0.351]) and heart failure (low-dose: 0.24, 0.07, to 0.77, *P* = 0.017, I^2^ = 0% [*P* = 0.764]; standard-dose: 0.24, 0.08 to 0.74, *P* = 0.020, I^2^ = not applicable; high-dose: 0.96, 0.58 to 1.57, P = 0.862, I^2^ = not applicable). The correspondent forest plots are presented in Additional file 1: Figures S16 to S18. As we can see, analysis in subgroups according to allopurinol dose reduced importantly the heterogeneity observed in the analysis of these outcomes.

We performed sensitivity analyses pooling the results of studies at low and unknown risk of bias (a categorization that embraces all randomized placebo-controlled trials presenting no clear evidence of high-risk of bias in any domain; see Methods). Studies at low and unknown risk of bias presented generally similar estimates of effect. (Additional file 1: Table S2). The results are presented in Additional file 1: Table S3, and show that most results were not significantly changed removing studies at high risk of bias, although a reduction in CV protective effect was generally observed. Additional sensitivity analyses (see Additional file 1: Supplementary Text 7) according to risk of bias, language of publication, and study duration corroborated the results previously presented.

To test for publication bias, funnel plot analyses and Egger’ tests for selected variables (MACE, total CV events, serious CV events, hypertension, and heart failure) were made (see Additional file 1: Figure S20 A-E). The graphics were generally fairly symmetrical and Egger’s test did not show significant differences, except for hypertension. Nevertheless, trim and fill analysis still suggested protection for hypertension (DerSimonian and Laird odds ratio: 0.63, 0.41 to 0.97, *P* = 0.038, I^2^ = 13% [*P* = 0.296]) after adjusting for publication bias. There was a reduction in funnel plot asymmetry when only allopurinol/oxypurinol studies were included in the analysis of hypertension (Additional file 1: Figure S21).

## Discussion

In the present study, we evaluated the effect of XOI on the incidence major cardiovascular events, death, and specific cardiovascular outcomes in adult patients participating in RCTs testing XOI versus placebo or no treatment. To the best of our knowledge, this is the first meta-analysis of RCTs to observe a protective effect of XOI for hard CV events. However, these results are not unexpected since there is already evidence from pooling of experimental studies suggesting benefits on blood pressure [28–30] and laboratory cardiovascular parameters [6, 8, 9, 31]. Despite the fact that our results were not positive for the primary outcomes in the entire set of studies, the analysis of secondary outcomes and subgroup analyses corroborate the hypothesis of CV protection promoted by XOI, especially in individuals at high-risk using purine-like XOI.

Several mechanisms have been proposed to explain the CV benefits of XOI. The anti-hypertensive effect may be mediated by reduction in levels of uric acid, a metabolite that promotes a pro-inflammatory state activating the renin-angiotensin system, reducing nitric oxide, and promoting vascular smooth-cells proliferation [1, 2]. The blockade of the activity of xanthine oxidase, which generates reactive oxygen species (ROS) along the process of transformation of hipoxanthine in uric acid, may reduce damage to endothelial cells and myocardium [1, 2]. Improvement of endothelial dependent vasodilation [6, 8, 9], oxidative stress markers [6, 7], and arterial stiffness [31] have been demonstrated in previous meta-analyses of experimental studies testing the effects of purine-like XOI.

Our results disagree with the conclusions of a recent meta-analysis addressing this subject, which found no significant CV protection with the use of hypouricemiant drugs [32]. However the comparison of this meta-analysis with ours is difficult because the authors selected only studies in gout (so the number of studies and events was smaller), the combined effects of all hypouricemiants (not only XOI) were compared against placebo, and the effects of XOI were compared directly with other hypouricemiant drugs. The inclusion of studies testing uricosurics or pegloticase, which reduce uric acid levels but are not supposed to present anti-oxidant properties, may possibly dilute a beneficial CV effect of purine-like XOI. In addition, in the beginning of hypouricemiant treatment in gout there may an increase in inflammatory activity that may blunt a long-term CV protective effect [1, 32]. This hypothesis may also possibly explain the lack of short term CV benefits after initiation of XOI in patients with hyperuricemia and gout observed in the cohort study by Kim et al. [14].

However, similar to our own results, the meta-analysis of Zhang and Pope [32] observed no CV protection with the use of febuxostat, and almost an increase in risk in comparison to allopurinol in the long term. Our study was not designed to compare the effects of the different types of XOI, but non-purine-like XOI failed to show reduction of CV risk. Keeping in mind that the confidence intervals were wide, these results are somehow unexpected given that allopurinol inhibits only the reduced form of xanthine oxidase, in comparison to non-purine-like XOI, which inhibit both isoforms of the enzyme, resulting theoretically in a more extensive blockade of production of ROS [1]. There are studies suggesting benefits of febuxostat on endothelial function [7] and even better improvement in CV parameters in patients treated with febuxostat in comparison to allopurinol [33, 34]. There is a RCT under way that may help to solve this question [35].

We observed significant heterogeneity in the analysis of total CV events, serious CV events, and heart failure associated with the use of purine-like XOI, and meta-regression analysis suggested that there is a positive dose-effect relationship with worse outcomes. Higher doses of allopurinol (> 300 mg) did not significantly reduce the incidence of heart failure and total and serious CV events, while low and standard doses were protective for these outcomes. These results were driven mostly by a RCT in established heart failure [18] and, despite the evidence from observational studies [5, 36, 37], it is possible that XOI are specifically ineffective in this setting [38]. Removing heart failure studies from our meta-analysis would actually eliminate heterogeneity and reinforce the protective CV effect of XOI. However, we propose an alternative explanation in which higher doses of allopurinol may lead to loss of cardiovascular protection, considering that higher oxypurinol concentrations may actually promote oxidative stress, as suggested by the study of Stamp et al. [39]. They observed that higher concentrations of oxypurinol promote a switch from an antioxidant to a pro-oxidant state, given that oxypurinol is a good substrate for myeloperoxidase (released by neutrophils in inflammatory states, such as gout and hyperuricemia), generating radicals capable of oxidizing urate and promoting deleterious effects [39]. The use of furosemide, which is highly prevalent in heart failure studies [18, 19], increases significantly (≥ 50%) the levels of oxypurinol [40, 41], and so may further contribute to increased oxidative stress. Better effects with lower doses were already observed in a meta-analysis evaluating the effects of allopurinol on blood pressure [30]. Lower doses are also probably safer, especially in patients with renal dysfunction [21] and during start of treatment [42].

Among all studies included in the meta-analysis, only the OPT-CHF trial (Hare et al. [19]), a relatively large heart failure study, actually suggested increase in adverse CV events associated with the use of purine-like XOI. Instead of allopurinol, this study used treatment with oxypurinol (Cardiome Pharma Corporation, Vancouver, British Columbia, Canada), a preparation that has lower bioavailability but has not been compared directly with allopurinol in continuous use. The observation of results of the OPT-CHF [19] and the La Plata [43] studies, both using oxypurinol 600 mg a day, permit estimate that this dose produced a reduction of 20–25% in uric acid concentration in relation to placebo. The Exact-HF study [18], performed in a similar setting (heart failure, but with hyperuricemia) utilizing allopurinol 600 mg/day as treatment, observed a reduction of approximately 33% in uric acid level in relation to placebo. So, the hypouricemiant effects of both drugs are comparable, suggesting that repeated oral administration of oxypurinol may also produce relatively high serum concentrations of oxypurinol, especially if used along with furosemide, leading potentially to a pro-oxidant state. Another possible problem of the OPT-CHF trial may be related to a random imbalance in baseline prognostic factors. Ejection fraction tended to be lower (25.3 ± 13.1 versus 27.7 ± 13.4 in placebo, *P* = 0.069) and BNP levels were numerically higher in the oxypurinol group, possibly indicating worse prognosis in this group.

Our study was several strengths. We performed an extensive literature search and used no language restriction, failing to obtain only a small number of articles. Most studies (79%) included predominantly individuals at increased risk of CV events. Only RCTs were included, excluding observational and non-randomized studies, reducing the risk of selection and confounding bias. We also removed all studies where any kind of co-intervention differed between the study groups to avoid bias. The results were consistent across several sensitivity and subgroup analyses, and funnel plot analysis did not in general suggest high risk of publication bias. All analyses presented in this article were described a priori in the study protocol published in PROSPERO. We excluded data of follow-up periods off-treatment given that our objective was to evaluate patients on continuous treatment, reflecting better the way these medications are used in medical practice.

The present study also has limitations. One of these is that cardiovascular and other adverse events were not the primary outcomes of most studies included in the meta-analysis. Therefore, the cardiovascular events of interest had in general not clearly pre-defined diagnostic criteria and were not centrally adjudicated. There was a significant heterogeneity among the studies included in this meta-analysis in terms of study duration, primary outcomes, and patients features. Although this is not ideal, meta-analysis of rare events is expected to include studies that are heterogeneous in various aspects, and this may be an advantage regarding the generalizability of results [27]. However, we are aware that our results must be considered exploratory and hypothesis generating [44], and definite conclusions must await the results of high-quality RCTs.

A significant proportion of the studies in this meta-analysis were classified as presenting high-risk of bias, a category that includes all non-blinded and/or non-placebo-controlled studies. As we can see in Additional file 1: Table S2, studies at high-risk of bias tended to be more optimistic in relation to the protective CV effects of XOI. It is difficult to define clearly how the absence of blinding may influence our results, but clinically significant adverse events are difficult to overlook in a reasonably conducted RCT. There was a significant association of high-risk of bias with use of lower doses of allopurinol, which in turn were related to better protective CV effect, creating room for confounding. However, sensitivity analyses considering the risk of bias still pointed to a cardiovascular protective effect of allopurinol at lower doses.

Despite our efforts to obtain all possible published and unpublished studies, a few could not be obtained in full-text, and a significant number of articles (50 studies, approximately 1200 patients/years of follow-up) had no adequate information on adverse events. The missing studies, as well as those with no extractable data, were in general older or smaller studies published only in the form of abstracts, or written in non-English language. Cross-over studies frequently did not report adverse events, or reported them in a manner that we could not define if it occurred before or after crossing over. However, funnel plot analysis did not suggest that publication bias played an important role in most outcomes.

We excluded from the analysis studies in which the patients used XOI for a short period of time (less than 4 weeks) and also studies whose follow-up period was longer than the duration of active treatment. Therefore, we did not evaluate the possibility of long-lasting effects of use of XOI for short periods after acute cardiovascular events. However the long-term follow-up of Goicoechea et al. [45], as well as the studies by Xiao et al. [46] and Huang et al. [47], suggest that the CV benefits of the use of XOI may persist after the medication is interrupted. The minimum duration of study of 4 weeks was chosen because we considered less likely a protective effect before this time interval [48] and the fact that most severe skin reactions occur around 1 month of use of allopurinol [49].

Some studies (21%) included in the meta-analysis included predominantly individuals without cardiovascular disease or risk factors, and several studies were small and followed-up the subjects for a relatively short period of time. Consequently, the number of MACE was relatively small, and the confidence intervals were wide. However, exclusion of studies whose individuals where not necessarily at high risk for CV events was associated with even better protective effect of XOI for CV events (see Results). In addition, removing studies lasting less than 90 days and 180 days did not significantly reduce (and actually improved) estimates of the effects of purine-like XOI (see Additional file 1: Supplementary Text 7 and Figures S22 and S23).

## Conclusions

Our results, based on low- to moderate quality of evidence, suggest that purine-like XOI reduce of incidence of adverse CV events (including acute ischemic coronary events and hypertension), especially among high-risk patients. However, the use of high doses of allopurinol (> 300 mg/day), especially with furosemide, may be associated with loss of CV protection.

## Additional files

Additional file 1: Supplementary file containing additional information on Methods and Results. (PDF 4649 kb)

Additional file 2: Supplementary file containing description of studies included in the meta-analysis. (PDF 703 kb)

Additional file 3: Supplementary file containing description of studies excluded from the meta-analysis after evaluation in full-text format. (PDF 410 kb)

Additional file 4: Study protocol. Study protocol registered in PROSPERO. (PDF 100 kb)

Additional file 5: Data bank. Shortened version of the data bank in CSV (comma-separated values) format. (CSV 25 kb)

## Abbreviations

CV: Cardiovascular
MACE: Major adverse cardiovascular events
OR: Odds ratio
PRISMA: Preferred Reporting Items for Systematic Reviews and Meta-Analyses
PROSPERO: International Prospective Register of Systematic Reviews
RCT: Randomized controlled trial
ROS: Reactive oxygen species
TCE: Total cardiovascular events
XOI: Xanthine oxidase inhibitors
