# Supplementary material for: Xanthine oxidase inhibitors for prevention of cardiovascular events: a systematic review and meta-analysis of randomized controlled trials
Source: BMC Cardiovasc Disord. 2018 Feb 7;18:24. doi: 10.1186/s12872-018-0757-9 (PMC5804046; doi:10.1186/s12872-018-0757-9)
Supplement: Supplementary file 1 — Supplementary file containing additional information on Methods and Results. (PDF 4649 kb) [file 12872_2018_757_MOESM1_ESM.pdf]

ADDITIONAL FILE 1

Xanthine Oxidase Inhibitors for Prevention of Cardiovascular Events: A Systematic Review and Meta-Analysis of Randomized Controlled Trials

Markus Bredemeier, Lediane Moreira Lopes, Matheus Augusto Eisenreich, Sheila Hickmann, Guilherme Kopik Bongiorno, Rui d'Avila, André Luis Bittencourt Morsch, Fernando da Silva Stein, Guilherme Gomes Dias Campos.

BMC Cardiovascular Disorders

INDEX

Supplementary Text 1: PUBMED search strategy …………………...………...……….. 4

Supplementary Text 2: EMBASE search strategy……………………………....………..6

Supplementary Text 3: Web of Science search strategy….……………………………………8

Supplementary Text 4: Lilacs search strategy………………………………………………9

Supplementary Text 5: Additional information on Methods…………………………..…..10

Supplementary Text 6: Interrater reliability and validation of use of Google translate™ ..13

Figure S1: Summary of initial evidence search and selection of the systematic review…..16

Figure S2: Summary of evidence search and selection to update the systematic review up to January 11, 2016………..……………………………………………….….……….17

Figure S3: Summary of evidence search and selection and of hand search process to update the systematic review up to December 30, 2016….…………..…………..……...18

Table S1: Evaluation of risk of bias……………………………………………….…..19

Figure S4: Forest plot comparing the risk of death between xanthine oxidase inhibitors and control…………………………………………….……………………………22

Figure S5: Forest plot comparing the risk of MACE between xanthine oxidase inhibitors and control in patients with previous transient ischemic attacks, stroke, unstable angina or myocardial infarction…………………………………...…………………………..23

Figure S6: Forest plot comparing the risk of myocardial infarction or urgent revascularization between xanthine oxidase inhibitors and control…………………….…...24

Figure S7: Forest plot comparing the risk of new/worsening hypertension between xanthine oxidase inhibitors and control……………………………….……………….…….….….…25

Figure S8: Forest plot comparing the risk of new/worsening heart failure between xanthine oxidase inhibitors and control………………………………………………...………...26

Figure S9: Forest plot comparing the risk of total cardiovascular events between xanthine oxidase inhibitors and control ….……………………………………….………..…....27

Figure S10: Forest plot comparing the risk of serious cardiovascular events between xanthine oxidase inhibitors and control ………..……………………..………………..28

Figure S11: Forest plot comparing the risk of myocardial infarction/urgent revascularizatiom between allopurinol/oxypurinol and control………………………...….29

Figure S12: Forest plot comparing the risk of new/worsening hypertension between allopurinol/oxypurinol and control ………..…………………………..……………..30

Figure S13: Forest plot comparing the risk of new/worsening heart failure between allopurinol/oxypurinol and control…………………………………..…………….…..….31

Figure S14: Forest plot comparing the risk of total cardiovascular events between allopurinol/oxypurinol and control …………………………..……………………...….32

Figure S15: Forest plot comparing the risk of serious cardiovascular events between allopurinol/oxypurinol and control …………………….…………..………………….33

Figure S16: Forest Plot comparing the risk of total cardiovascular events of low-dose, standard-dose, and high-dose of allopurinol versus control ……………..…………….…...34

Figure S17: Forest Plot comparing the risk of serious cardiovascular events of low-dose, standard-dose, and high-dose of allopurinol versus control …………………..……...35

Figure S18: Forest Plot comparing the risk of new/worsening heart failure of low-dose, standard-dose, and high-dose of allopurinol versus control…………………..……....36

Table S2: Subgroup analyses of MACE and secondary outcomes according to the risk of bias …………………………………………….……..……………………….…...37

Table S3: Sensitivity analyses excluding from analyses studies at high risk of bias ……..39

Supplementary text 7: Additional information on sensitivity analysis…………………...41

Figure S19: Random effects meta-regression analyses of dose of allopurinol and log odds ratio of new/worsening heart failure (A), total cardiovascular events (B), and serious cardiovascular events (C) among studies at low or unknown risk of bias……………..….44

Figure S20: Funnel plot analyses of the outcomes major cardiovascular events (A), serious cardiovascular events (B), total cardiovascular events (C), new/worsening hypertension (D), and new/worsening heart failure (E)…………………………….……….45

Figure S21: Funnel plot analysis of the outcome new/worsening hypertension in allopurinol/oxypurinol studies…………………………………...………………..…47

Figure S22: Forest plot comparing the risk of total cardiovascular events of allopurinol/oxypurinol versus control among studies lasting 90 days or longer……....….48

Figure S23: Forest plot comparing the risk of MACE of allopurinol/oxypurinol versus control among studies lasting 180 days or longer……………………………………..….49

Supplementary Text 1: PUBMED search strategy (similar to the strategy used in Cochrane Library).

#7. #1 and (#2 or #3 or #4 or #5 or #6)

#6. Alopurinol or Allopurinol [Mesh] or Zyloprim or Wellcome Brand of Allopurinol or Allopurinol Wellcome Brand or Zyloric or Glaxo Wellcome Brand of Allopurinol or Allopurin or Bicther Brand of Allopurinol or Allopurinol Bicther Brand or Allorin or Douglas Brand of Allopurinol or Allopurinol Douglas Brand or Allpargin or Merz Brand of Allopurinol or Allopurinol Merz Brand or Allural or Pan Quimica or Quimica, Pan or Apulonga or Dorsch Brand of Allopurinol or Allopurinol Dorsch Brand or Apurin or Multipharma Brand of Allopurinol or Allopurinol Multipharma Brand or Atisuril or Byk Gulden Brand of Allopurinol or Bleminol or gepepharm Brand of Allopurinol or Allopurinol gepepharm Brand or Caplenal or Rhône-Poulenc Rorer Brand of Allopurinol or Rhône Poulenc Rorer Brand of Allopurinol or APS Brand of Allopurinol or Allopurinol APS Brand or Capurate or Fawns and McAllan Brand of Allopurinol or Cellidrin or Hennig Brand of Allopurinol or Allopurinol Hennig Brand or Embarin or Suspendol or Merckle Brand of Allopurinol or Allopurinol Merckle Brand or Foligan or Henning Berlin Brand of Allopurinol or Hamarin or Nicholas Brand of Allopurinol or Allopurinol Nicholas Brand or Jenapurinol or Jenapharm Brand of Allopurinol or Allopurinol Jenapharm Brand or Lopurin or Boots Brand of Allopurinol or Allopurinol Boots Brand or Lysuron or Boehringer Mannheim Brand of Allopurinol or Milurit or Thiemann Brand of Allopurinol or Allopurinol Thiemann Brand or Milurite or Novopurol or Novopharm Brand of Allopurinol or Allopurinol Novopharm Brand or Progout or Protea Brand of Allopurinol or Allopurinol Protea Brand or Alphapharm Brand of Allopurinol or Allopurinol Alphapharm Brand or Pureduct or Rosen Brand of Allopurinol or Allopurinol Rosen Brand or Purinol or Pinewood Brand of Allopurinol or Allopurinol Pinewood Brand or Horner Brand of Allopurinol or Allopurinol Horner Brand or Remid or TAD Brand of Allopurinol or Allopurinol TAD Brand or Rimapurinol or Rima Brand of Allopurinol or Allopurinol Rima Brand or Roucol or Rougier Brand of Allopurinol or Allopurinol Rougier Brand or Tipuric or Clonmel Brand of Allopurinol or Allopurinol Clonmel Brand or Uribenz or R.A.N. Brand of Allopurinol or Allopurinol R.A.N. Brand or Uridocid or Reig Jofre Brand of Allopurinol or Uripurinol or Azupharma Brand of Allopurinol or Allopurinol Azupharma Brand or Urosin or Roche Brand of Allopurinol or Allopurinol Roche Brand or Urtias or BASF Brand of Allopurinol or Allopurinol BASF Brand or Xanthomax or Ashbourne Brand of Allopurinol or Allopurinol Ashbourne Brand or Xanturic or Pharmafarm Brand of Allopurinol or Allopurinol Pharmafarm Brand or Zygout or Amrad Brand of Allopurinol or Allopurinol Amrad Brand or Allohexal or Hexal Brand of Allopurinol or Allopurinol Hexal Brand or Allohexan or Alloprin or ICN Brand of Allopurinol or Allopurinol ICN Brand

#5. febuxostat or "febuxostat" [Supplementary Concept] or “Uloric” or “2-(3-cyano-4-isobutoxyphenyl)-4-methyl-5-thiazolecarboxylic acid” or “TEI 6720” or “TEI-6720” or TEI6720

#4. "Oxypurinol"[Mesh] or Oxipurinol or Alloxanthine or tisopurine or "tisopurine" [Supplementary Concept] or "thiopurinol" or "4-mercaptopyrazolo(3,4-d)pyrimidine" or topiroxostat or Uriadec or topiloric

#3. allosig or puricos or Ossipurinolo or Oxoallopurinol or Oxypurinolum or Alloxanthin or DHPP or Adenuric

#2. Xanthine Oxidase [Mesh] or Oxidase, Xanthine or Hypoxanthine Oxidase or Oxidase, Hypoxanthine or Purine-Xanthine Oxidase or Oxidase, Purine-Xanthine or Purine Xanthine Oxidase or Hypoxanthine Dehydrogenase or Dehydrogenase, Hypoxanthine or Hypoxanthine-Xanthine Oxidase or Hypoxanthine Xanthine Oxidase or Oxidase, Hypoxanthine-Xanthine or Xanthine Dehydrogenase [Mesh] or Dehydrogenase, Xanthine or Purine Hydroxylase I or Xanthine Oxidoreductase or Oxidoreductase, Xanthine

#1. (randomized controlled trial[pt] OR controlled clinical trial[pt] OR randomized controlled trials[mh] OR random allocation[mh] OR double-blind method[mh] OR single-blind method[mh] OR clinical trial[pt] OR clinical trials[mh] OR ("clinical trial"[tw]) OR ((singl*[tw] OR doubl*[tw] OR trebl*[tw] OR tripl*[tw]) AND (mask*[tw] OR blind*[tw])) OR ("latin square"[tw]) OR placebos[mh] OR placebo*[tw] OR random*[tw] OR research design[mh:noexp] OR follow-up studies[mh] OR prospective studies[mh] OR cross-over studies[mh] OR control*[tw] OR prospectiv*[tw] OR volunteer*[tw]) NOT (animal[mh] NOT human[mh])

Supplementary Text 2: EMBASE search strategy.

#10

#7 AND #8 AND [6-1-2016]/sd NOT [31-12-2016]/sd

363

#9

#7 AND #8

4,532

#8

#1 OR #2 OR #3 OR #4 OR #5 OR #6

30,030

#7

((**'crossover procedure'**/exp OR **'crossover procedure'**) AND [embase]/lim OR ((**'prospective study'**/exp OR **'prospective study'**) AND [embase]/lim) OR ((**'follow up'**/exp OR **'follow up'**) AND [embase]/lim) OR ((**'placebo'**/exp OR **'placebo'**) AND [embase]/lim) OR ((**'clinical trial'**/exp OR **'clinical trial'**) AND [embase]/lim) OR ((**'single blind procedure'**/exp OR **'single blind procedure'**) AND [embase]/lim) OR ((**'double blind procedure'**/exp OR **'double blind procedure'**) AND [embase]/lim) OR ((**'triple blind procedure'**/exp OR **'triple blind procedure'**) AND [embase]/lim) OR ((**'randomization'**/exp OR **'randomization'**) AND [embase]/lim) OR ((**'controlled clinical trial'**/exp OR **'controlled clinical trial'**) AND [embase]/lim) OR ((**'randomized controlled trial'**/exp OR **'randomized controlled trial'**) AND [embase]/lim)) NOT ((**'animals'**/exp OR **'animals'**) NOT (**'humans'**/exp OR **'humans'**))

2,763,792

#6

(**'allopurinol'**/exp OR **'allopurinol'**) AND [embase]/lim

19,773

#5

(**'xanthine oxidase'**/exp OR **'xanthine oxidase'**) AND [embase]/lim

12,790

#4

(**'allosig'** OR **'allosig'**/exp OR **allosig** OR **'puricos'** OR **'puricos'**/exp OR **puricos** OR **ossipurinolo** OR **oxoallopurinol** OR **oxypurinolum** OR **'alloxanthin'** OR **'alloxanthin'**/exp OR **alloxanthin** OR **dhpp** OR **'adenuric'** OR **'adenuric'**/exp OR **adenuric**) AND [embase]/lim

19,811

#3

(**'oxipurinol'**/exp OR **'oxipurinol'** OR **'oxypurinol'**/exp OR **oxypurinol**) AND [embase]/lim

1,192

#2

(**'topiroxostat'**/exp OR **'topiroxostat'**) AND [embase]/lim

41

#1

(**'febuxostat'**/exp OR **'febuxostat'**) AND [embase]/lim

1,455

Supplementary Text 3: Web of Science search strategy.

(clinical trial*) OR (research design) OR (comparative stud*) OR (evaluation stud*) OR (controlled trial*) OR (follow-up stud*) OR (prospective stud*) OR (random*) OR (placebo*) OR (single blind*) OR (double blind*) or (triple blind*) or (double dummy) or (allocation) ➔ TOPIC

AND

alopurinol or allopurinol or Zyloprim or Zyloric or Allopurin or Allorin or Allpargin or Allural or "Pan Quimica" or "Quimica, Pan" or Apulonga or Apurin or Atisuril or Bleminol or Caplenal or Capurate or Cellidrin or Embarin or Suspendol or Foligan or Hamarin or Jenapurinol or Lopurin or Lysuron or Milurit or Milurite or Novopurol or Progout or Pureduct or Purinol or Remid or Rimapurinol or Roucol or Tipuric or Uribenz or Uridocid or Uripurinol or Urosin or Urtias or Xanthomax or Xanturic or Zygout or Allohexal or Allohexan or Alloprin or febuxostate or febuxostat or Uloric or “2-3-cyano-4-isobutoxyphenyl-4-methyl-5-thiazolecarboxylic acid” or “TEI 6720” or “TEI-6720” or TEI6720 or Oxypurinol or Oxipurinol or Alloxanthine or tisopurine or tisopurine or thiopurinol or "4-mercaptopyrazolo(3,4-d)pyrimidine" or topiroxostat or Uriadec or topiloric or allosig or puricos or Ossipurinolo or Oxoallopurinol or Oxypurinolum or Alloxanthin or DHPP or Adenuric or (Xanthine and oxidase) or (hypoxantine and oxidase) or (xanthine and dehydrogenase) or (hypoxanthine and dehydrogenase) or (xantina and oxidase) or (hipoxantina and oxidase) or (xantina and desidrogenase) or (hipoxantina and desidrogenase) or (xantina and oxidasa) or (hipoxantina and oxidasa) or (xanthine and oxidoreductase) or (hypoxanthine and oxidoreductase) or (xantina and oxiredutase) or (hipoxantina and oxiredutase) or (purine and hydroxylase)→ TOPIC

Supplementary Text 4: Lilacs search strategy.

((PT:"randomized controlled trial**" OR PT:"**controlled clinical trial**" OR PT:"**multicenter study**" OR MH:"**randomized controlled trials as topic**" OR MH:"**controlled clinical trials as topic**" OR MH:"**multicenter study as topic**" OR MH:"**random allocation**" OR MH:"**double-blind method" **OR MH:"**single-blind method"**) OR ((**ensaio**$ OR** ensayo**$ OR** trial**$) AND (**azar **OR** acaso **OR** placebo **OR** control**$ OR** aleat**$ OR** random**$ OR** enmascarado**$ OR** simpleciego **OR ((**simple**$ OR** single **OR** duplo**$ OR** doble**$ OR** double**$) AND (**cego **OR** ciego **OR** blind **OR** mask**))) AND** clinic**$)) AND NOT ((MH:**animals **OR MH:**rabbits **OR MH:**rats **OR MH:**primates **OR MH:**dogs **OR MH:**cats **OR MH:**swine **OR PT:"**in vitro**") AND NOT MH:humans)**

AND

alopurinol or allopurinol or Zyloprim or Zyloric or Allopurin or Allorin or Allpargin or Allural or "Pan Quimica" or "Quimica, Pan" or Apulonga or Apurin or Atisuril or Bleminol or Caplenal or Capurate or Cellidrin or Embarin or Suspendol or Foligan or Hamarin or Jenapurinol or Lopurin or Lysuron or Milurit or Milurite or Novopurol or Progout or Pureduct or Purinol or Remid or Rimapurinol or Roucol or Tipuric or Uribenz or Uridocid or Uripurinol or Urosin or Urtias or Xanthomax or Xanturic or Zygout or Allohexal or Allohexan or Alloprin or febuxostate or febuxostat or Uloric or “2-3-cyano-4-isobutoxyphenyl-4-methyl-5-thiazolecarboxylic acid” or “TEI 6720” or “TEI-6720” or TEI6720 or Oxypurinol or Oxipurinol or Alloxanthine or tisopurine or tisopurine or thiopurinol or "4-mercaptopyrazolo(3,4-d)pyrimidine" or topiroxostat or Uriadec or topiloric or allosig or puricos or Ossipurinolo or Oxoallopurinol or Oxypurinolum or Alloxanthin or DHPP or Adenuric or (Xanthine and oxidase) or (hypoxantine and oxidase) or (xanthine and dehydrogenase) or (hypoxanthine and dehydrogenase) or (xantina and oxidase) or (hipoxantina and oxidase) or (xantina and desidrogenase) or (hipoxantina and desidrogenase) or (xantina and oxidasa) or (hipoxantina and oxidasa) or (xanthine and oxidoreductase) or (hypoxanthine and oxidoreductase) or (xantina and oxiredutase) or (hipoxantina and oxiredutase) or (purine and hydroxylase)

Supplementary Text 5: Additional information on Methods.

Data Sources and Searches (complementary information).

Hand searching was performed scrutinizing the reference lists of systematic reviews and original articles, searching the ClinicalTrials.gov, Clinicaltrialsregister.eu, and Google™ web sites, searching the abstracts of the American College of Rheumatology meetings from 1989 to 2000 and from 2009 to 2016 (abstracts from 2001 to 2008 are not available online), and searching EULAR abstracts archive from 2002 to 2016. A PubMed and Web of Science "second look" search using a limited strategy ([allopurinol or febuxostat or tisopurine or topiroxostat or oxypurinol] and [random* or "clinical trial" or placebo]) was made and updated to December 30, 2016 (beginning from inception in PubMed and from Jan 1, 2016 in Web of Science) to identify studies previously missed. A similar strategy was used to search articles published in Chinese using the China/Asia on Demand ™ (CAOD, Oriprobe Information Services) site up to December 29, 2016.

Articles in languages other than English, German, Portuguese, or Spanish (which would be able to be read and interpreted by the authors or close contacts) were translated using Google Translate™ whenever possible. Part of the articles in Chinese (6 in total) were translated to Portuguese by a specialized professional, but other 26 articles (of which 10 were included in the final analysis, but only 3 of them contributed with events) were translated using Google Translate™. We compared data extracted using both methods in a blinded fashion, and there was excellent agreement (See Supplementary Text 6 in Additional file 1).

Data Extraction and Quality Assessment (complementary information).

In case of cross-over randomized controlled trials, only data on the adverse events observed in the first period of the study (before crossing-over) were considered. If the adverse events (or absence of it) could not be clearly ascertained to the period before crossing-over, the study was excluded.

In case of studies presenting more than one arm using different XOI (one arm receiving allopurinol and other receiving febuxostat, for example), data of these arms were extracted separately. When diverse doses of the same XOI were used in different arms of a given study, we pooled the data in a single group considering the mean dose received by the patients for analytical purposes. If the dose of the medications received by the patients (within a given study arm) were adjusted according to renal function, we considered the mean and standard deviation of creatinine level or glomerular filtration rate (whichever was reported by the authors), and then used the Z scores to estimate the prevalence of each dosing, and finally estimated the average dose using the weighted mean formula.

The Cochrane method for evaluation of bias describes seven components: random sequence generation, allocation concealment, blinding of participants and personnel, blinding of outcome assessors, incomplete outcome data, selective reporting, and other possible sources of bias. The risk of bias in the domains related to blinding was considered high for non-placebo controlled studies, and unknown for studies not describing clearly the blinding procedures. Studies not describing appropriately the cardiovascular events of interest we classified of high risk of bias for the component selective reporting. The study was judged to be at low risk of bias if the first 4 components and selective reporting were all low risk of bias, with no other component at high risk of bias; other studies were

deemed to be at unknown risk of bias, or high risk of bias if some component was at high-risk.

Supplementary Text 6: Interrater reliability and validation of use of Google translate^{TM}.

We performed the translations from Chinese using the latest version of Google Translate ^{TM} during the months of January to March 2017. This version utilizes a new system of translation called GNMT (Google Neural Machine Translation system), which has shown to have near human accuracy in translation from Chinese to English (Wu Y, Schuster M, Chen Z, Le QV, Norouzi M, Macherey W, et al. Google's neural machine translation system: Bridging the gap between human and machine translation. arXiv preprint arXiv:1609.08144, 2016). This system also enables 'zero-shot' translations, allowing direct translation between pairs of languages that the system has not been trained on (Johnson M, Schuster M, Le QV, Krikun M, Wu Y, Chen Z, et al. Google's multilingual neural machine translation system: Enabling zero-shot translation. arXiv preprint arXiv:1611.04558, 2016).

Considering the use of Arabic numbers and the relatively straightforward structure of articles in Chinese, we selected and transferred to Google Translate^{TM} all parts (separately) of the articles using the copy/paste functions. There was generally no necessity of translating the abstract and references, which were most frequently written in English. When a translated sentence had not a coherent structure or significance, we selected isolated parts of the phrase or even smaller conjuncts of characters to be translated separately. Some phrases had to be translated word by word to ensure clarity of the information. The candidate articles were translated to English or Portuguese to permit data extraction; sometimes, the language that the sentences were translated to was changed to obtain better clarity of the information. All translations were reassessed for coherence of

the information at least once again (using only translation from Chinese to English) by the main investigator during the process data bank cleaning.

To evaluate reliability of the translation of articles in Chinese, we compared (in a blinded fashion) data extracted by a couple of researchers (MAE and LML) who used articles translated by a professional Chinese translator with data extracted by another couple (GKB and SH) using versions of the same articles translated using Google translate^{TM}. Reliability for qualitative variables was tested using the Kappa statistic, and intraclass correlation coefficient (ICC) was used to assess agreement between the couples on numerical variables. ICC was tested using IBM SPSS Statistics^{TM} version 20 (model: two-way mixed; type: absolute agreement; single measures). For this analysis, we considered the qualitative and quantitative variables that could be extracted data from 6 articles (translated using both methods) and results are presented without considering the clustering represented by each article.

Qualitative variables — medication, comparison group (placebo or no treatment), target population:

Bootstrap for Symmetric Measures

		Value	Bootstrap[a]			
			Bias	Std. Error	95% Confidence Interval	
					Lower	Upper
Measure of Agreement	Kappa	1,000	,000	,000	1,000	1,000
N of Valid Cases		24	0	0	24	24

a. Unless otherwise noted, bootstrap results are based on 1000 bootstrap samples

Quantitative variables — age, prevalence of male gender, prevalence of comorbidities, mean creatinine clearance, study duration, allopurinol dose, mean baseline uric acid, prevalence of specific medications, number of participants, number of cardiovascular events, adverse reactions, loss of follow-up*:

* Not all articles presented extractable data for all variables listed above.

Intraclass Correlation Coefficient

	Intraclass Correlation[b]	95% Confidence Interval		F Test with True Value 0			
		Lower Bound	Upper Bound	Value	df1	df2	Sig
Single Measures	,995[a]	,991	,997	379,749	55	55	,000
Average Measures	,997[c]	,996	,998	379,749	55	55	,000

Two-way mixed effects model where people effects are random and measures effects are fixed.

a. The estimator is the same, whether the interaction effect is present or not.

b. Type A intraclass correlation coefficients using an absolute agreement definition.

c. This estimate is computed assuming the interaction effect is absent, because it is not estimable otherwise.

The results suggest excellent agreement between data extracted from articles translated by a human translator and by GNMT. We verified the differences between data obtained by both methods of translation, and all were actually problems caused by errors in extraction or typing of data (not in translation). After that, all data extraction and typing were checked again by the main investigator, and eventual mistakes were corrected.

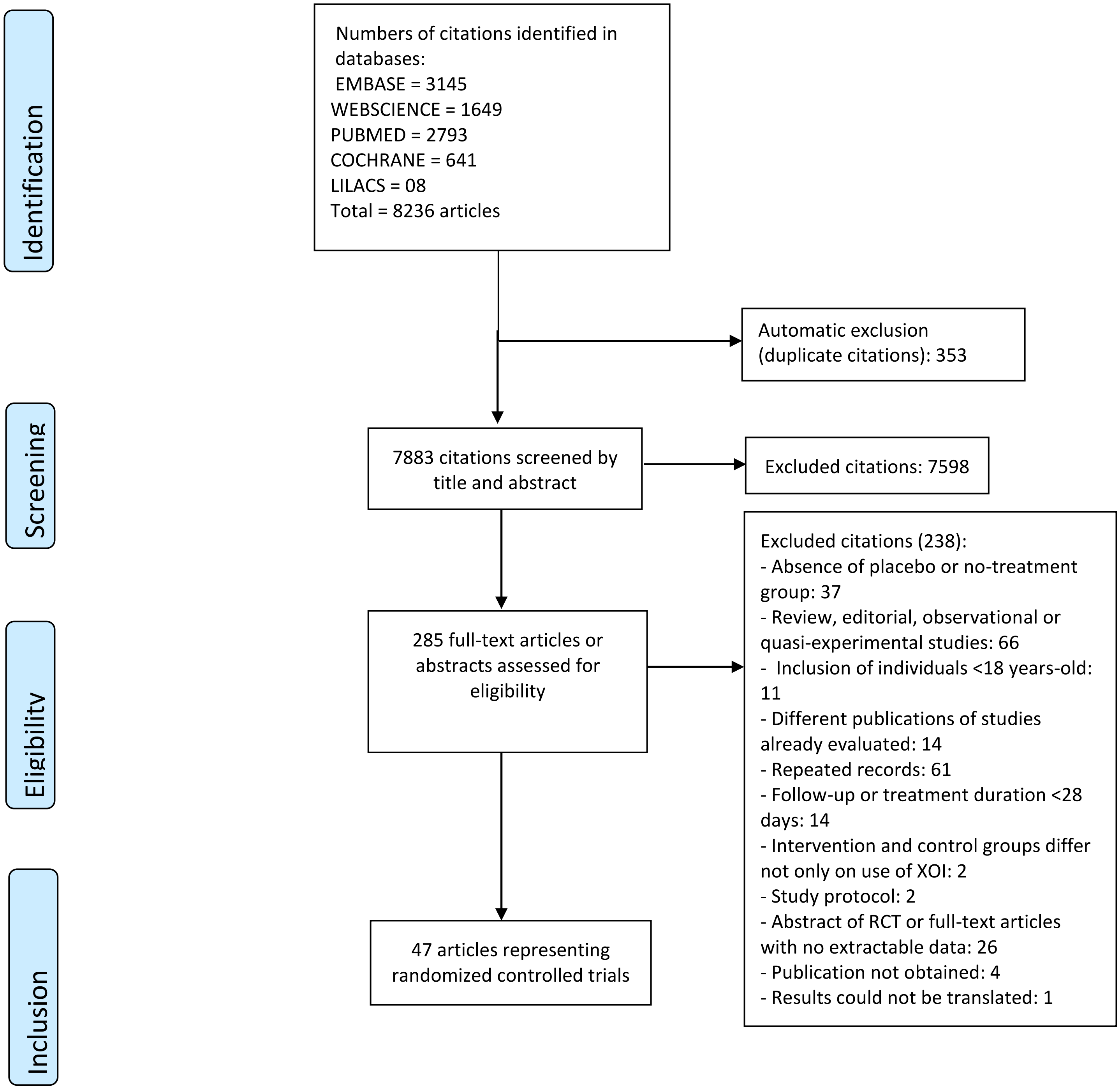

Figure S1: Summary of initial evidence search and selection of the systematic review (September 29, 2014).

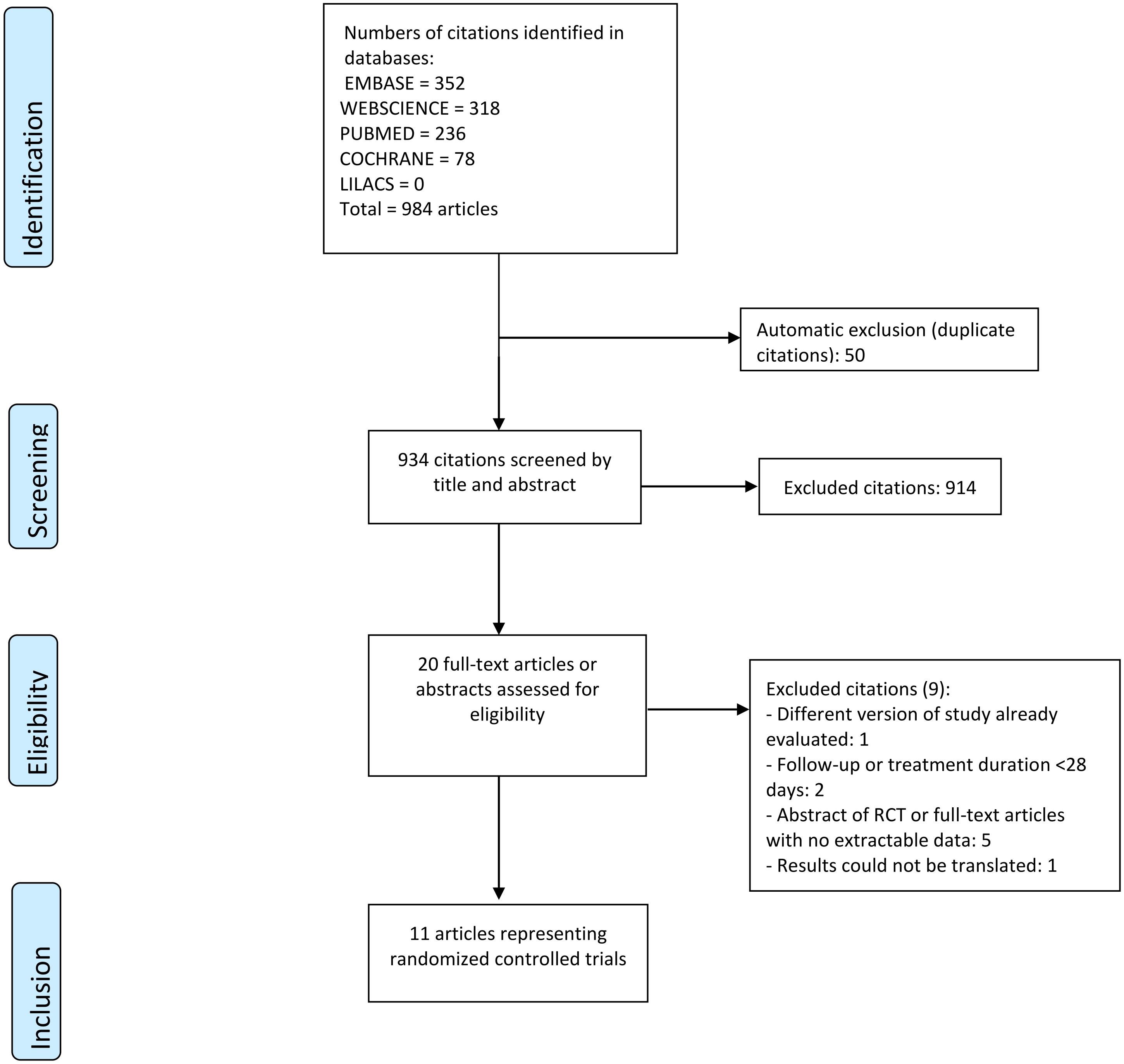

Figure S2: Summary of evidence search and selection to update the systematic review (from September 29, 2014 to January 11, 2016).

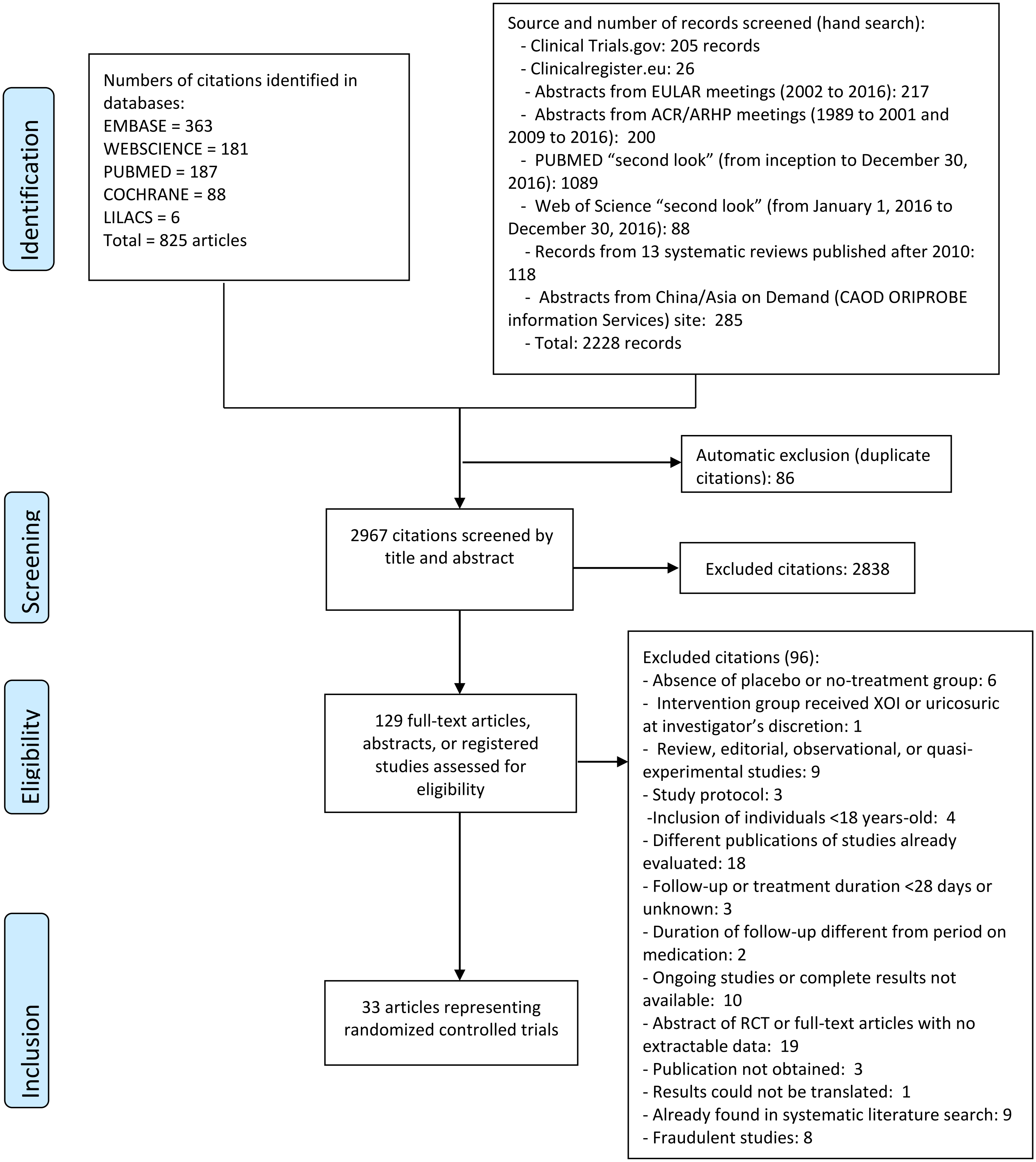

Figure S3: Summary of evidence search and selection and of hand search process to update the systematic review from January 11, 2016 to December 30, 2016.

Table S1: Evaluation of risk of bias.

Study/Year	RANDOM SEQUENCE GENERATION	ALLOCATION CONCEALMENT	BLINDING OF PARTICIPANTS AND PERSONNEL	BLINDING OF OUTCOME ASSESSMENT	INCOMPLETE OUTCOME DATA	SELECTIVE REPORTING	OTHER BIAS	OVERALL RISK OF BIAS
Akhondzadeh 2005	L	U	L	L	L	L	L	U
Akhondzadeh 2006	U	U	U	U	L	L	L	U
Becker 2005	U	U	U	U	L	L	L	U
Borgi 2017	L	L	L	L	L	L	L	L
Bowden 2013	U	U	U	U	L	U	L	U
Brunstein 2005	U	U	U	U	L	L	L	U
Chen 2009	U	U	H	H	L	U	U	H
Chen 2014	U	U	H	H	L	U	U	H
Cingolani 2006	U	U	L	L	L	L	L	U
Dawson 2009	L	L	L	L	U	L	U	L
Deng 2010	U	U	H	H	U	U	H	H
Dickerson 2009	U	U	U	U	L	L	L	U
Dogan 2011	U	U	H	H	L	U	L	H
Eddeland 1983	L	U	U	U	L	L	L	U
Ettinger 1986	U	U	U	U	L	H	L	H
Fan 2012	U	U	U	U	L	U	L	U
Feuerman 1973	U	U	L	L	L	U	U	U
George 2006	U	U	U	U	L	U	L	U
Gibson 1982	U	U	H	H	L	H	U	H
Givertz 2015	L	L	L	L	L	L	L	L
Goicoechea 2010	L	U	H	H	L	L	L	H
Goldfarb 2013	U	U	L	L	L	L	L	U
Greig 2011	L	U	U	U	L	L	L	U
Guo 2015	L	U	H	H	L	U	U	H
Guo 2016	U	U	H	H	U	U	U	H
Hare 2008	U	U	U	U	L	L	L	U
Higgins 2014	L	L	L	L	L	L	L	L
Hill 2015	L	L	L	L	L	L	L	L
Hosoya 2014	U	U	U	U	L	L	L	U
Hosoya 2016 fase 2a	L	L	L	L	L	L	L	L
Hosoya 2016 fase 2b	L	L	L	L	L	L	L	L
Jahangard 2014	L	L	L	L	L	U	L	L
Jalal 2016	L	L	U	U	L	L	L	U
Jalalzadeh 2012	U	U	H	H	U	U	L	H
Jarnerot 2000	U	U	U	U	L	L	L	U

Jitapunkul 1991	U	U	U	U	L	U	L	U
Joelsson 2001	U	U	U	U	L	U	L	U
Kamatani 2011 late phase 2	L	L	U	U	L	L	L	U
Kamatani 2011 phase 3	L	L	L	L	L	L	L	L
Kanbay 2011	L	U	H	H	L	U	L	H
Kao 2011	U	U	U	U	L	L	L	U
Khan 2008	U	U	L	L	L	L	L	U
Lei 2009	U	U	H	H	H	L	U	H
Liu 2007	U	U	H	H	U	U	H	H
Liu 2015	L	U	H	H	H	L	L	H
Machado Vieira 2008	U	U	L	L	L	L	L	U
Madero 2015	L	L	L	L	L	L	L	L
Mao 2015	L	U	U	U	U	U	U	U
Modabber 2009	U	U	U	U	L	L	L	U
Momeni 2010	U	U	U	U	L	L	L	U
Muir 2008	L	L	L	L	L	L	U	L
NCT01078389 2014	U	U	L	L	L	L	L	U
NCT01350388 2016	U	U	L	L	L	L	L	U
NCT01496469 2015	U	U	L	L	L	L	L	U
NCT02128490 / Gunawardhana 2016	U	U	L	L	L	L	L	U
NCT02139046 / Saag 2016	U	U	L	L	L	L	L	U
Noman 2010	L	L	L	L	L	L	L	L
Parmley 1992	U	U	U	U	H	H	L	H
Poiley 2016	U	U	L	L	L	L	L	U
Puntoni 2013	L	L	L	L	L	L	L	L
Rassi 2007	L	L	L	L	L	L	L	L
Rekhraj 2013	L	U	U	L	L	U	L	U
Rentoukas 2010	U	U	U	U	L	L	L	U
Robertson 2015	L	L	L	L	L	L	L	L
Rosenfeld 1974	U	U	H	H	L	H	U	H
Saag 2016	U	U	L	L	L	L	U	U
Sarris 2007	U	U	H	H	L	H	U	H
Schumacher 2008	U	U	L	L	L	L	L	U
Segal 2015	U	U	L	L	L	L	L	U
Separham 2016	L	U	U	L	L	L	L	U
Shen 2010	U	U	H	H	U	U	H	H
Shi 2012	L	L	H	H	L	L	L	H
Sircar 2015	L	L	L	L	L	L	L	L
Siu 2006	L	U	H	H	L	U	L	H
Szwejkowski 2013	L	U	L	L	L	U	L	U
Taheraghdam 2014	L	L	L	L	L	L	L	L
Takir 2015	U	H	H	H	L	U	U	H
Tan 2014	U	U	H	H	L	L	U	H
Tanaka 2015	L	L	H	H	L	U	L	H
Tani 2015	U	L	H	H	L	L	L	H

Togha 2007	L	L	L	L	U	L	U	L
Tsuruta 2015	L	U	H	H	L	L	L	H
Usharani 2016	L	U	U	U	L	L	L	U
Wang 2012	L	U	H	H	U	L	U	H
Wang 2015	L	U	H	H	L	U	U	H
Weiser 2012	L	L	L	L	L	L	L	L
Weiser 2014	L	L	L	L	L	L	L	L
Yin 2015	U	U	H	H	L	L	U	H
Zhang 2012	U	U	H	H	L	L	U	H
Zhou 2009	U	U	H	H	U	H	H	H
Ziaee 2006	U	U	U	U	L	U	L	U

Studies	Estimate (95% C.I.)	Ev/Trt	Ev/Ctrl
Akhondzadeh 2005	1.000 (0.000, Inf)	0/23	0/23
Akhondzadeh 2006	1.000 (0.000, Inf)	0/41	0/41
Becker 2005	0.262 (0.000, Inf)	0/115	0/38
Borgi 2017	1.081 (0.000, Inf)	0/49	0/53
Bowden 2013	1.000 (0.000, Inf)	0/12	0/12
Brunstein 2005	0.920 (0.000, Inf)	0/12	0/11
Chen 2009	1.000 (0.000, Inf)	0/40	0/40
Chen 2014	0.135 (0.003, 6.820)	0/30	1/30
Cingolani 2006	1.000 (0.000, Inf)	0/30	0/30
Dawson 2009	1.085 (0.000, Inf)	0/23	0/25
Deng 2010	1.060 (0.000, Inf)	0/33	0/35
Dickerson 2009	0.905 (0.000, Inf)	0/31	0/28
Dogan 2011	1.000 (0.000, Inf)	0/50	0/50
Eddeland 1983	1.364 (0.206, 9.052)	3/16	2/14
Ettinger 1986	1.000 (0.000, Inf)	0/36	0/36
Fan 2012	0.920 (0.000, Inf)	0/12	0/11
Feuerman 1973	1.154 (0.000, Inf)	0/6	0/7
George 2006	0.484 (0.000, Inf)	0/20	0/10
Gibson 1982	1.267 (0.000, Inf)	0/26	0/33
Givertz 2015	1.123 (0.396, 3.182)	8/128	7/125
Goicoechea 2010	0.131 (0.008, 2.113)	0/57	2/56
Goldfarb 2013	0.476 (0.000, Inf)	0/66	0/33
Greig 2011	0.948 (0.000, Inf)	0/38	0/36
Guo 2015	0.132 (0.008, 2.147)	0/40	2/40
Guo 2016	2.385 (0.536, 10.605)	5/215	2/215
Hare 2008	1.672 (0.616, 4.540)	10/203	6/202
Higgins 2014	0.975 (0.000, Inf)	0/40	0/39
Hill 2013	1.183 (0.000, Inf)	0/16	0/19
Hosoya 2014	0.984 (0.000, Inf)	0/62	0/61
Hosoya 2016 Phase 2a	0.140 (0.000, Inf)	0/151	0/36
Hosoya 2016 Phase 2b	3.783 (0.041, 353.019)	1/118	0/39
Jahangard 2014	0.901 (0.000, Inf)	0/30	0/27
Jalal 2016	7.778 (0.154, 392.460)	1/39	0/41
Jalalzadeh 2012	0.965 (0.000, Inf)	0/28	0/27
Jarnerot 2000	0.970 (0.000, Inf)	0/98	0/95
Jitapunkul 1991	1.000 (0.000, Inf)	0/26	0/26
Joelsson 2001	7.081 (0.140, 357.202)	1/94	0/90
Kamatani 2011 late phase 2	0.131 (0.000, Inf)	0/164	0/38
Kamatani 2011 phase 3	0.450 (0.000, Inf)	0/69	0/33
Kanbay 2011	1.248 (0.000, Inf)	0/32	0/40
Kao 2011	0.140 (0.003, 7.048)	0/30	1/31
Khan 2008	1.000 (0.000, Inf)	0/15	0/15
Lei 2009	0.966 (0.000, Inf)	0/29	0/28
Liu 2007	0.959 (0.000, Inf)	0/24	0/23
Liu 2015	1.000 (0.000, Inf)	0/88	0/88
Machado Vieira 2008	1.000 (0.000, Inf)	0/60	0/60
Madero 2015	1.000 (0.000, Inf)	0/36	0/36
Mao 2015	0.135 (0.003, 6.820)	0/366	1/366
Modabber 2009	0.905 (0.000, Inf)	0/10	0/9
Momeni 2010	1.000 (0.000, Inf)	0/20	0/20
Muir 2008	0.445 (0.000, Inf)	0/34	0/16
NCT01078389 2014	1.000 (0.062, 16.059)	1/157	1/157
NCT01350388 2016	1.000 (0.000, Inf)	0/40	0/40
NCT01496469 2015	0.984 (0.000, Inf)	0/61	0/60
NCT02128490 / Gunawardhana 2016	3.496 (0.026, 464.931)	1/151	0/38
NCT02139046 / Saag 2016	0.433 (0.025, 7.352)	2/1427	1/356
Noman 2010	1.095 (0.000, Inf)	0/31	0/34
Parmley 1992	1.000 (0.308, 3.249)	6/71	6/71
Poiley 2016	0.487 (0.000, Inf)	0/55	0/28
Puntoni 2013	0.465 (0.000, Inf)	0/49	0/24
Rassi 2007	0.508 (0.000, Inf)	0/23	0/12
Rekhraj 2013	1.000 (0.000, Inf)	0/33	0/33
Robertson 2015	1.000 (0.000, Inf)	0/25	0/25
Rosenfeld 1974	1.000 (0.195, 5.136)	3/58	3/58
Saag 2016	0.216 (0.019, 2.445)	1/64	2/32
Sarris 2007	1.000 (0.000, Inf)	0/18	0/18
Schumacher 2008	0.033 (0.000, Inf)	0/938	0/134
Segal 2015	1.014 (0.000, Inf)	0/69	0/70
Separham 2016	0.501 (0.098, 2.556)	2/70	4/70
Shen 2010	1.000 (0.000, Inf)	0/26	0/26
Shi 2012	0.907 (0.000, Inf)	0/21	0/19
Sircar 2015	1.000 (0.000, Inf)	0/54	0/54
Siu 2006	1.076 (0.000, Inf)	0/26	0/28
Szwejkowski 2013	1.000 (0.000, Inf)	0/33	0/33
Taheraghdam 2014	0.470 (0.117, 1.888)	3/35	6/35
Takir 2015	0.826 (0.000, Inf)	0/40	0/33
Tan 2014	0.983 (0.000, Inf)	0/57	0/56
Tanaka 2015	0.803 (0.000, Inf)	0/25	0/20
Tani 2015	1.000 (0.000, Inf)	0/30	0/30
Togha 2007	1.108 (0.000, Inf)	0/18	0/20
Tsuruta 2015	1.000 (0.000, Inf)	0/27	0/27
Usharani 2016	0.946 (0.000, Inf)	0/18	0/17
Wang 2012	1.034 (0.000, Inf)	0/29	0/30
Wang 2015	0.977 (0.000, Inf)	0/43	0/42
Weiser 2012	1.016 (0.000, Inf)	0/123	0/125
Weiser 2014	1.000 (0.000, Inf)	0/90	0/90
Yin 2015	0.928 (0.000, Inf)	0/41	0/38
Zhang 2012	0.491 (0.094, 2.565)	2/40	4/40
Zhou 2009	0.922 (0.000, Inf)	0/51	0/47
Ziaee 2006	0.932 (0.000, Inf)	0/29	0/27
**Overall**	0.890 (0.594, 1.334)	50/7227	51/4634

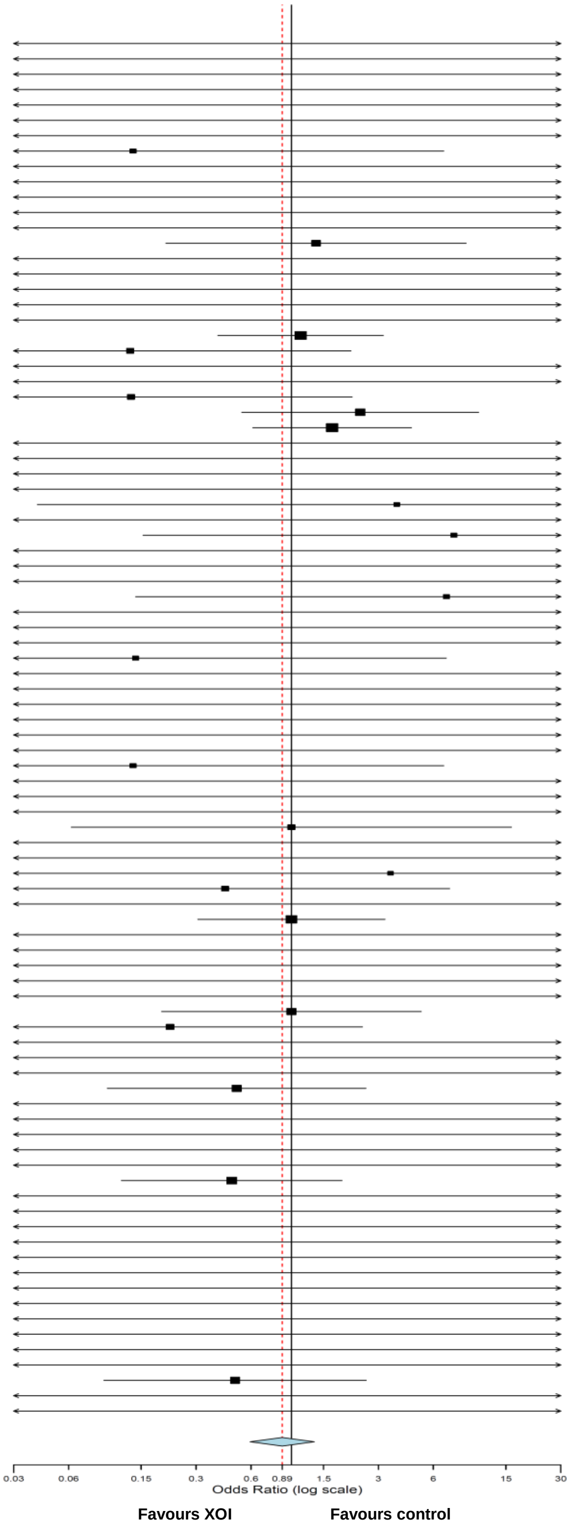

Figure S4: Forest plot comparing the risk of death between xanthine oxidase inhibitors and control. Numbers are Peto odds ratio and 95% CI. Heterogeneity: I^2= 0%, Cochran´s Q test: P=0.704.

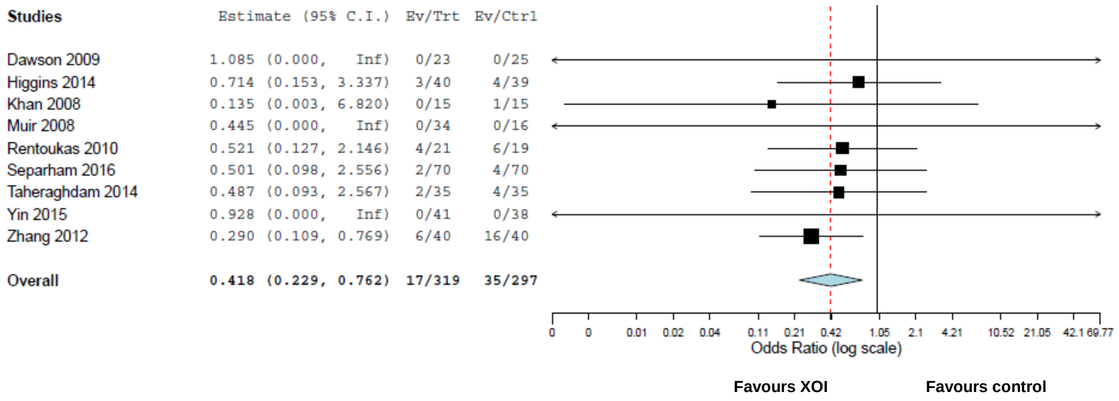

Figure S5: Forest plot comparing the risk of MACE between xanthine oxidase inhibitors and control in patients with previous transient ischemic attacks, stroke, unstable angina or myocardial infarction. Numbers are Peto odds ratio and 95% CI. Heterogeneity: I^2=0%, Cochran´s Q test: P=0.914.

Studies	Estimate (95% C.I.)	Ev/Trt	Ev/Ctrl
Akhondzadeh 2005	1.000 (0.000, Inf)	0/23	0/23
Akhondzadeh 2006	1.000 (0.000, Inf)	0/41	0/41
Becker 2005	0.262 (0.000, Inf)	0/115	0/38
Borgi 2017	1.081 (0.000, Inf)	0/49	0/53
Bowden 2013	1.000 (0.000, Inf)	0/12	0/12
Brunstein 2005	0.920 (0.000, Inf)	0/12	0/11
Chen 2009	1.000 (0.000, Inf)	0/40	0/40
Cingolani 2006	1.000 (0.000, Inf)	0/30	0/30
Dawson 2009	1.085 (0.000, Inf)	0/23	0/25
Deng 2010	1.060 (0.000, Inf)	0/33	0/35
Dickerson 2009	6.707 (0.132, 339.757)	1/31	0/28
Dogan 2011	1.000 (0.000, Inf)	0/50	0/50
Fan 2012	0.920 (0.000, Inf)	0/12	0/11
Feuerman 1973	1.154 (0.000, Inf)	0/6	0/7
George 2006	0.484 (0.000, Inf)	0/20	0/10
Gibson 1982	1.267 (0.000, Inf)	0/26	0/33
Givertz 2015	0.977 (0.000, Inf)	0/128	0/125
Goldfarb 2013	0.476 (0.000, Inf)	0/66	0/33
Greig 2011	0.948 (0.000, Inf)	0/38	0/36
Higgins 2014	0.975 (0.060, 15.868)	1/40	1/39
Hill 2013	1.183 (0.000, Inf)	0/16	0/19
Hosoya 2014	0.984 (0.000, Inf)	0/62	0/61
Hosoya 2016 Phase 2a	0.140 (0.000, Inf)	0/151	0/36
Hosoya 2016 Phase 2b	0.262 (0.000, Inf)	0/118	0/39
Jahangard 2014	0.901 (0.000, Inf)	0/30	0/27
Jalalzadeh 2012	0.965 (0.000, Inf)	0/28	0/27
Jarnerot 2000	0.970 (0.000, Inf)	0/98	0/95
Jitapunkul 1991	1.000 (0.000, Inf)	0/26	0/26
Joelsson 2001	0.958 (0.000, Inf)	0/94	0/90
Kamatani 2011 late phase 2	0.131 (0.000, Inf)	0/164	0/38
Kamatani 2011 phase 3	0.450 (0.000, Inf)	0/69	0/33
Kanbay 2011	1.248 (0.000, Inf)	0/32	0/40
Khan 2008	1.000 (0.000, Inf)	0/15	0/15
Lei 2009	0.966 (0.000, Inf)	0/29	0/28
Liu 2007	0.959 (0.000, Inf)	0/24	0/23
Liu 2015	1.000 (0.000, Inf)	0/88	0/88
Machado Vieira 2008	1.000 (0.000, Inf)	0/60	0/60
Madero 2015	1.000 (0.000, Inf)	0/36	0/36
Mao 2015	0.135 (0.003, 6.820)	0/366	1/366
Modabber 2009	0.905 (0.000, Inf)	0/10	0/9
Momeni 2010	1.000 (0.000, Inf)	0/20	0/20
Muir 2008	0.445 (0.000, Inf)	0/34	0/16
NCT01078389 2014	7.437 (0.463, 119.421)	2/157	0/157
NCT01350388 2016	1.000 (0.000, Inf)	0/40	0/40
NCT01496469 2015	7.269 (0.144, 366.379)	1/61	0/60
NCT02128490 / Gunawardhana 2016	3.496 (0.026, 464.931)	1/151	0/38
NCT02139046 / Saag 2016	3.489 (0.026, 469.882)	1/1427	0/356
Noman 2010	1.095 (0.000, Inf)	0/31	0/34
Parmley 1992	0.360 (0.050, 2.610)	1/71	3/71
Poiley 2016	0.487 (0.000, Inf)	0/55	0/28
Puntoni 2013	0.465 (0.000, Inf)	0/49	0/24
Rassi 2007	0.508 (0.000, Inf)	0/23	0/12
Rekhraj 2013	1.000 (0.000, Inf)	0/33	0/33
Robertson 2015	1.000 (0.000, Inf)	0/25	0/25
Saag 2016	0.216 (0.019, 2.445)	1/64	2/32
Sarris 2007	1.000 (0.000, Inf)	0/18	0/18
Schumacher 2008	3.142 (0.102, 96.524)	3/938	0/134
Segal 2015	1.014 (0.000, Inf)	0/69	0/70
Shen 2010	1.000 (0.000, Inf)	0/26	0/26
Shi 2012	0.907 (0.000, Inf)	0/21	0/19
Sircar 2015	1.000 (0.000, Inf)	0/54	0/54
Siu 2006	1.076 (0.000, Inf)	0/26	0/28
Szwejkowski 2013	1.000 (0.000, Inf)	0/33	0/33
Taheraghdam 2014	0.352 (0.047, 2.608)	1/35	3/35
Takir 2015	0.826 (0.000, Inf)	0/40	0/33
Tanaka 2015	0.803 (0.000, Inf)	0/25	0/20
Tani 2015	1.000 (0.000, Inf)	0/30	0/30
Togha 2007	1.108 (0.000, Inf)	0/18	0/20
Tsuruta 2015	1.000 (0.000, Inf)	0/27	0/27
Usharani 2016	0.946 (0.000, Inf)	0/18	0/17
Wang 2012	1.034 (0.000, Inf)	0/29	0/30
Wang 2015	0.977 (0.000, Inf)	0/43	0/42
Weiser 2012	1.016 (0.000, Inf)	0/123	0/125
Weiser 2014	1.000 (0.000, Inf)	0/90	0/90
Yin 2015	0.928 (0.000, Inf)	0/41	0/38
Zhang 2012	0.291 (0.098, 0.865)	4/40	12/40
Zhou 2009	0.922 (0.000, Inf)	0/51	0/47
Ziaee 2006	0.932 (0.000, Inf)	0/29	0/27
**Overall**	**0.560 (0.283, 1.109)**	**17/6376**	**22/3785**

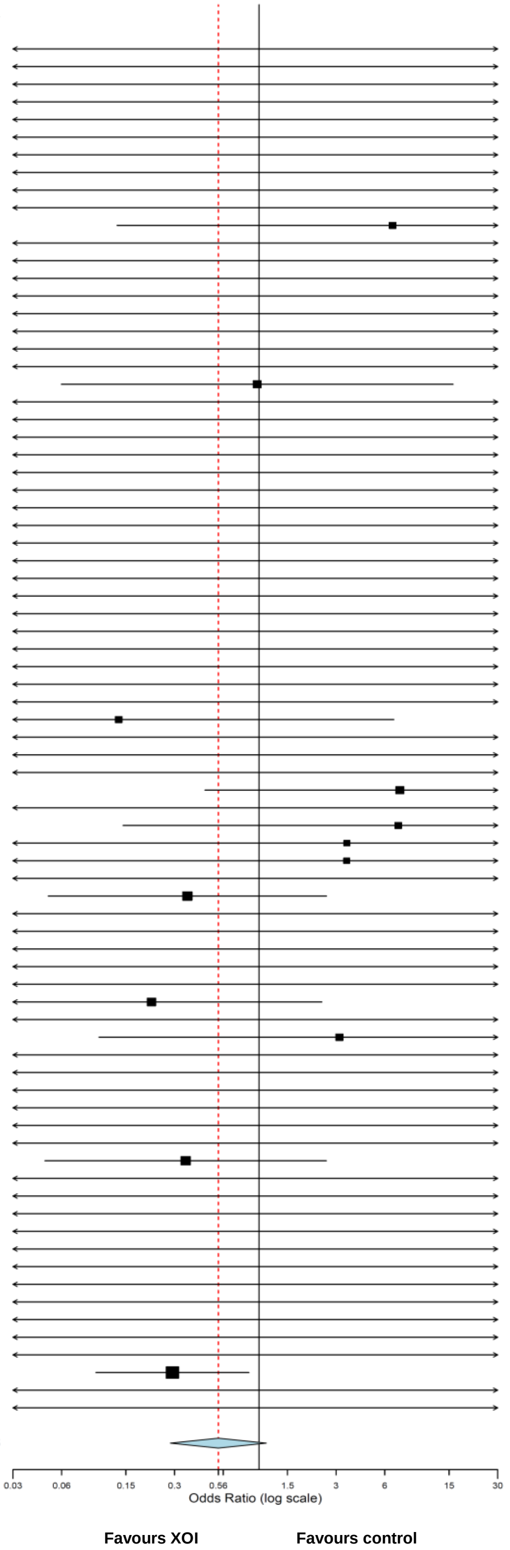

Figure S6: Forest plot comparing the risk of myocardial infarction or urgent revascularization between xanthine oxidase inhibitors and control. Numbers are Peto odds ratio and 95% CI. Heterogeneity: I^2= 5%, Cochran´s Q test: P=0.395.

Studies	Estimate (95% C.I.)	Ev/Trt	Ev/Ctrl
Akhondzadeh 2005	1.000 (0.000, Inf)	0/23	0/23
Akhondzadeh 2006	1.000 (0.000, Inf)	0/41	0/41
Becker 2005	0.262 (0.000, Inf)	0/115	0/38
Borgi 2017	1.081 (0.000, Inf)	0/49	0/53
Bowden 2013	1.000 (0.000, Inf)	0/12	0/12
Brunstein 2005	0.920 (0.000, Inf)	0/12	0/11
Chen 2009	1.000 (0.000, Inf)	0/40	0/40
Cingolani 2006	1.000 (0.000, Inf)	0/30	0/30
Dawson 2009	1.085 (0.000, Inf)	0/23	0/25
Deng 2010	1.060 (0.000, Inf)	0/33	0/35
Dickerson 2009	0.905 (0.000, Inf)	0/31	0/28
Dogan 2011	1.000 (0.000, Inf)	0/50	0/50
Fan 2012	0.920 (0.000, Inf)	0/12	0/11
Feuerman 1973	1.154 (0.000, Inf)	0/6	0/7
George 2006	0.484 (0.000, Inf)	0/20	0/10
Gibson 1982	1.267 (0.000, Inf)	0/26	0/33
Givertz 2015	0.650 (0.111, 3.806)	2/128	3/125
Goldfarb 2013	0.476 (0.000, Inf)	0/66	0/33
Greig 2011	0.948 (0.000, Inf)	0/38	0/36
Higgins 2014	0.975 (0.000, Inf)	0/40	0/39
Hill 2013	1.183 (0.000, Inf)	0/16	0/19
Hosoya 2014	0.984 (0.000, Inf)	0/62	0/61
Hosoya 2016 Phase 2a	0.140 (0.000, Inf)	0/151	0/36
Hosoya 2016 Phase 2b	0.262 (0.000, Inf)	0/118	0/39
Jahangard 2014	0.901 (0.000, Inf)	0/30	0/27
Jarnerot 2000	0.970 (0.000, Inf)	0/98	0/95
Jitapunkul 1991	1.000 (0.000, Inf)	0/26	0/26
Joelsson 2001	0.958 (0.000, Inf)	0/94	0/90
Kamatani 2011 late phase 2	0.042 (0.002, 0.775)	1/164	2/38
Kamatani 2011 phase 3	0.450 (0.000, Inf)	0/69	0/33
Kanbay 2011	1.248 (0.000, Inf)	0/32	0/40
Kao 2011	0.400 (0.084, 1.907)	2/30	5/31
Khan 2008	1.000 (0.000, Inf)	0/15	0/15
Lei 2009	0.966 (0.000, Inf)	0/29	0/28
Liu 2007	0.959 (0.000, Inf)	0/24	0/23
Liu 2015	0.478 (0.181, 1.264)	6/88	12/88
Machado Vieira 2008	1.000 (0.000, Inf)	0/60	0/60
Madero 2015	0.135 (0.003, 6.820)	0/36	1/36
Modabber 2009	0.905 (0.000, Inf)	0/10	0/9
Momeni 2010	1.000 (0.000, Inf)	0/20	0/20
Muir 2008	0.445 (0.000, Inf)	0/34	0/16
NCT01078389 2014	1.150 (0.408, 3.239)	8/157	7/157
NCT01350388 2016	1.000 (0.000, Inf)	0/40	0/40
NCT01496469 2015	0.133 (0.003, 6.709)	0/61	1/60
NCT02128490 / Gunawardhana 2016	1.454 (0.222, 9.515)	6/151	1/38
NCT02139046 / Saag 2016	0.782 (0.364, 1.681)	32/1427	10/356
Noman 2010	1.095 (0.000, Inf)	0/31	0/34
Poiley 2016	0.221 (0.020, 2.492)	1/55	2/28
Puntoni 2013	0.465 (0.000, Inf)	0/49	0/24
Rassi 2007	0.508 (0.000, Inf)	0/23	0/12
Rekhraj 2013	1.000 (0.000, Inf)	0/33	0/33
Robertson 2015	1.000 (0.000, Inf)	0/25	0/25
Saag 2016	0.216 (0.019, 2.445)	1/64	2/32
Sarris 2007	1.000 (0.000, Inf)	0/18	0/18
Schumacher 2008	0.399 (0.146, 1.089)	28/938	8/134
Shi 2012	0.109 (0.011, 1.113)	0/21	3/19
Sircar 2015	1.000 (0.000, Inf)	0/54	0/54
Siu 2006	1.076 (0.000, Inf)	0/26	0/28
Szwejkowski 2013	1.000 (0.000, Inf)	0/33	0/33
Takir 2015	0.826 (0.000, Inf)	0/40	0/33
Tanaka 2015	0.803 (0.000, Inf)	0/25	0/20
Tani 2015	1.000 (0.000, Inf)	0/30	0/30
Togha 2007	1.108 (0.000, Inf)	0/18	0/20
Tsuruta 2015	1.000 (0.000, Inf)	0/27	0/27
Usharani 2016	0.946 (0.000, Inf)	0/18	0/17
Wang 2012	1.034 (0.000, Inf)	0/29	0/30
Wang 2015	0.977 (0.000, Inf)	0/43	0/42
Weiser 2012	1.016 (0.000, Inf)	0/123	0/125
Weiser 2014	1.000 (0.000, Inf)	0/90	0/90
Yin 2015	0.928 (0.000, Inf)	0/41	0/38
Ziaee 2006	0.932 (0.000, Inf)	0/29	0/27
**Overall**	**0.543 (0.367, 0.805)**	**87/5720**	**57/3134**

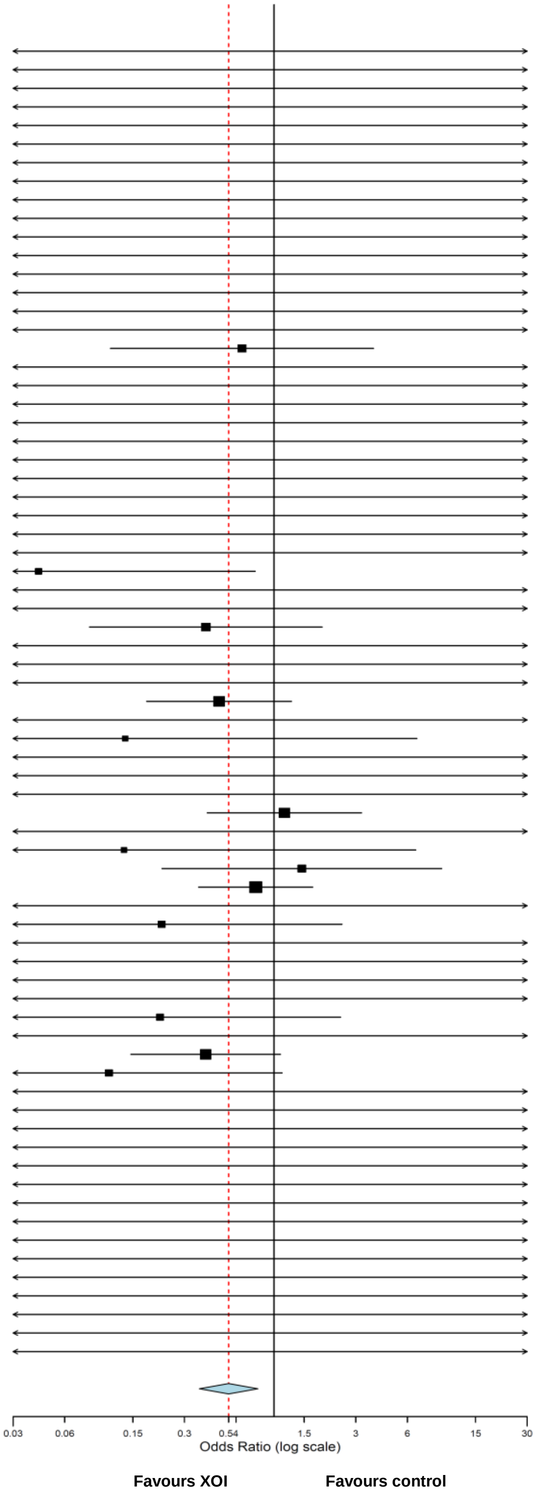

Figure S7: Forest plot comparing the risk of new/worsening hypertension between xanthine oxidase inhibitors and control. Numbers are Peto odds ratio and 95% CI. Heterogeneity: I^2= 0%, Cochran´s Q test: P=0.494.

Studies	Estimate (95% C.I.)	Ev/Trt	Ev/Ctrl
Akhondzadeh 2005	1.000 (0.000, Inf)	0/23	0/23
Akhondzadeh 2006	1.000 (0.000, Inf)	0/41	0/41
Becker 2005	0.262 (0.000, Inf)	0/115	0/38
Borgi 2017	1.081 (0.000, Inf)	0/49	0/53
Bowden 2013	1.000 (0.000, Inf)	0/12	0/12
Brunstein 2005	0.920 (0.000, Inf)	0/12	0/11
Chen 2009	1.000 (0.000, Inf)	0/40	0/40
Cingolani 2006	1.000 (0.000, Inf)	0/30	0/30
Dawson 2009	1.085 (0.000, Inf)	0/23	0/25
Deng 2010	1.060 (0.000, Inf)	0/33	0/35
Dickerson 2009	0.905 (0.000, Inf)	0/31	0/28
Dogan 2011	1.000 (0.000, Inf)	0/50	0/50
Fan 2012	0.920 (0.000, Inf)	0/12	0/11
Feuerman 1973	1.154 (0.000, Inf)	0/6	0/7
George 2006	0.484 (0.000, Inf)	0/20	0/10
Gibson 1982	1.267 (0.000, Inf)	0/26	0/33
Givertz 2015	0.957 (0.584, 1.568)	58/128	58/125
Goldfarb 2013	0.476 (0.000, Inf)	0/66	0/33
Greig 2011	0.948 (0.000, Inf)	0/38	0/36
Hare 2008	1.465 (0.890, 2.411)	44/203	32/202
Higgins 2014	0.975 (0.000, Inf)	0/40	0/39
Hill 2013	1.183 (0.000, Inf)	0/16	0/19
Hosoya 2014	0.984 (0.000, Inf)	0/62	0/61
Hosoya 2016 Phase 2a	0.140 (0.000, Inf)	0/151	0/36
Hosoya 2016 Phase 2b	0.262 (0.000, Inf)	0/118	0/39
Jahangard 2014	0.901 (0.000, Inf)	0/30	0/27
Jalalzadeh 2012	0.965 (0.000, Inf)	0/28	0/27
Jarnerot 2000	0.970 (0.000, Inf)	0/98	0/95
Jitapunkul 1991	1.000 (0.000, Inf)	0/26	0/26
Joelsson 2001	0.958 (0.000, Inf)	0/94	0/90
Kamatani 2011 late phase 2	0.131 (0.000, Inf)	0/164	0/38
Kamatani 2011 phase 3	0.450 (0.000, Inf)	0/69	0/33
Kanbay 2011	1.248 (0.000, Inf)	0/32	0/40
Kao 2011	1.033 (0.000, Inf)	0/30	0/31
Khan 2008	1.000 (0.000, Inf)	0/15	0/15
Lei 2009	0.966 (0.000, Inf)	0/29	0/28
Liu 2007	0.959 (0.000, Inf)	0/24	0/23
Liu 2015	1.000 (0.000, Inf)	0/88	0/88
Machado Vieira 2008	1.000 (0.000, Inf)	0/60	0/60
Madero 2015	1.000 (0.000, Inf)	0/36	0/36
Modabber 2009	0.905 (0.000, Inf)	0/10	0/9
Momeni 2010	1.000 (0.000, Inf)	0/20	0/20
Muir 2008	0.445 (0.000, Inf)	0/34	0/16
NCT01078389 2014	1.000 (0.062, 16.059)	1/157	1/157
NCT01350388 2016	1.000 (0.000, Inf)	0/40	0/40
NCT01496469 2015	0.984 (0.000, Inf)	0/61	0/60
NCT02128490 / Gunawardhana 2016	0.157 (0.000, Inf)	0/151	0/38
NCT02139046 / Saag 2016	3.493 (0.206, 59.336)	3/1427	0/356
Noman 2010	1.095 (0.000, Inf)	0/31	0/34
Poiley 2016	0.487 (0.000, Inf)	0/55	0/28
Puntoni 2013	0.465 (0.000, Inf)	0/49	0/24
Rassi 2007	0.508 (0.000, Inf)	0/23	0/12
Rekhraj 2013	1.000 (0.000, Inf)	0/33	0/33
Robertson 2015	1.000 (0.000, Inf)	0/25	0/25
Saag 2016	4.629 (0.409, 52.373)	3/64	0/32
Sarris 2007	1.000 (0.000, Inf)	0/18	0/18
Separham 2016	0.254 (0.074, 0.865)	2/70	9/70
Shi 2012	0.907 (0.000, Inf)	0/21	0/19
Sircar 2015	1.000 (0.000, Inf)	0/54	0/54
Siu 2006	1.076 (0.000, Inf)	0/26	0/28
Szwejkowski 2013	1.000 (0.000, Inf)	0/33	0/33
Taheraghdam 2014	0.135 (0.003, 6.820)	0/35	1/35
Takir 2015	0.826 (0.000, Inf)	0/40	0/33
Tanaka 2015	0.803 (0.000, Inf)	0/25	0/20
Tani 2015	1.000 (0.000, Inf)	0/30	0/30
Togha 2007	1.108 (0.000, Inf)	0/18	0/20
Tsuruta 2015	0.135 (0.003, 6.820)	0/27	1/27
Usharani 2016	0.946 (0.000, Inf)	0/18	0/17
Wang 2012	1.034 (0.000, Inf)	0/29	0/30
Wang 2015	0.977 (0.000, Inf)	0/43	0/42
Weiser 2012	1.016 (0.000, Inf)	0/123	0/125
Weiser 2014	1.000 (0.000, Inf)	0/90	0/90
Yin 2015	0.928 (0.000, Inf)	0/41	0/38
Zhang 2012	0.268 (0.098, 0.733)	5/40	15/40
Ziaee 2006	0.932 (0.000, Inf)	0/29	0/27
**Overall**	**0.935 (0.685, 1.276)**	**116/5158**	**117/3374**

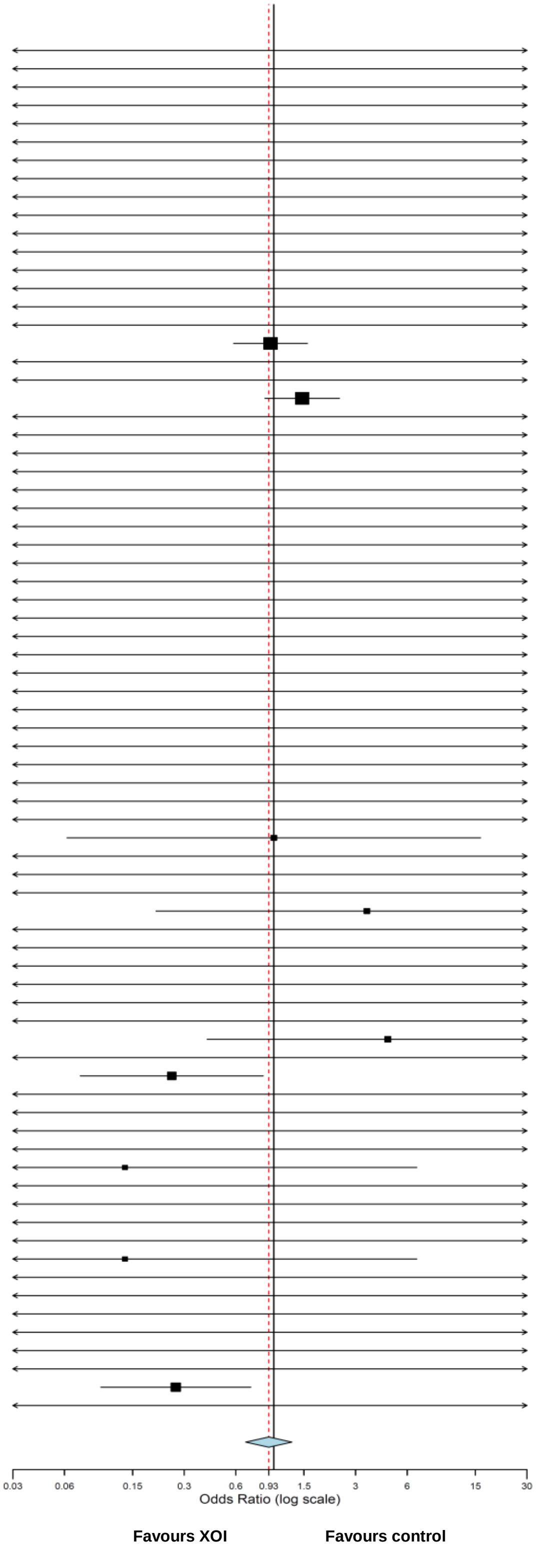

Figure S8: Forest plot comparing the risk of new/worsening heart failure between xanthine oxidase inhibitors and control. Numbers are Peto odds ratio and 95% CI. Heterogeneity: I^2=55%, Cochran´s Q test: P=0.023.

Studies	Estimate (95% C.I.)	Ev/Trt	Ev/Ctrl
Akhondzadeh 2005	1.000 (0.000, Inf)	0/23	0/23
Akhondzadeh 2006	1.000 (0.000, Inf)	0/41	0/41
Becker 2005	0.262 (0.000, Inf)	0/115	0/38
Borgi 2017	1.081 (0.000, Inf)	0/49	0/53
Bowden 2013	1.000 (0.000, Inf)	0/12	0/12
Brunstein 2005	0.920 (0.000, Inf)	0/12	0/11
Chen 2009	1.000 (0.000, Inf)	0/40	0/40
Cingolani 2006	1.000 (0.000, Inf)	0/30	0/30
Dawson 2009	1.085 (0.000, Inf)	0/23	0/25
Deng 2010	1.060 (0.000, Inf)	0/33	0/35
Dickerson 2009	6.707 (0.132, 339.757)	1/31	0/28
Dogan 2011	1.000 (0.000, Inf)	0/50	0/50
Fan 2012	0.920 (0.000, Inf)	0/12	0/11
Feuerman 1973	1.154 (0.000, Inf)	0/6	0/7
George 2006	0.484 (0.000, Inf)	0/20	0/10
Gibson 1982	1.267 (0.000, Inf)	0/26	0/33
Givertz 2015	0.957 (0.584, 1.568)	58/128	58/125
Goicoechea 2010	0.400 (0.158, 1.010)	7/57	15/56
Goldfarb 2013	0.476 (0.000, Inf)	0/66	0/33
Greig 2011	0.948 (0.000, Inf)	0/38	0/36
Hare 2008	1.465 (0.890, 2.411)	44/203	32/202
Higgins 2014	0.714 (0.153, 3.337)	3/40	4/39
Hill 2013	1.183 (0.000, Inf)	0/16	0/19
Hosoya 2014	0.984 (0.000, Inf)	0/62	0/61
Hosoya 2016 Phase 2a	0.140 (0.000, Inf)	0/151	0/36
Hosoya 2016 Phase 2b	0.262 (0.000, Inf)	0/118	0/39
Jahangard 2014	0.901 (0.000, Inf)	0/30	0/27
Jalal 2016	7.778 (0.154, 392.460)	1/39	0/41
Jalalzadeh 2012	0.965 (0.000, Inf)	0/28	0/27
Jarnerot 2000	7.241 (0.449, 116.640)	2/98	0/95
Jitapunkul 1991	1.000 (0.000, Inf)	0/26	0/26
Joelsson 2001	0.958 (0.000, Inf)	0/94	0/90
Kamatani 2011 late phase 2	0.126 (0.010, 1.574)	2/164	2/38
Kamatani 2011 phase 3	0.450 (0.000, Inf)	0/69	0/33
Kanbay 2011	1.248 (0.000, Inf)	0/32	0/40
Khan 2008	0.135 (0.003, 6.820)	0/15	1/15
Lei 2009	0.966 (0.000, Inf)	0/29	0/28
Liu 2007	0.959 (0.000, Inf)	0/24	0/23
Liu 2015	0.478 (0.181, 1.264)	6/88	12/88
Machado Vieira 2008	1.000 (0.000, Inf)	0/60	0/60
Madero 2015	0.135 (0.003, 6.820)	0/36	1/36
Mao 2015	0.327 (0.188, 0.569)	13/366	41/366
Modabber 2009	0.905 (0.000, Inf)	0/10	0/9
Momeni 2010	1.000 (0.000, Inf)	0/20	0/20
Muir 2008	0.445 (0.000, Inf)	0/34	0/16
NCT01078389 2014	1.000 (0.449, 2.229)	13/157	13/157
NCT01350388 2016	0.505 (0.051, 4.995)	1/40	2/40
NCT01496469 2015	0.983 (0.061, 15.909)	1/61	1/60
NCT02128490 / Gunawardhana 2016	2.178 (0.508, 9.331)	11/151	1/38
NCT02139046 / Saag 2016	0.998 (0.510, 1.953)	44/1427	11/356
Noman 2010	1.095 (0.000, Inf)	0/31	0/34
Poiley 2016	0.221 (0.020, 2.492)	1/55	2/28
Puntoni 2013	0.465 (0.000, Inf)	0/49	0/24
Rassi 2007	0.508 (0.000, Inf)	0/23	0/12
Rekhraj 2013	7.389 (0.147, 372.385)	1/33	0/33
Rentoukas 2010	0.521 (0.127, 2.146)	4/21	6/19
Robertson 2015	1.000 (0.000, Inf)	0/25	0/25
Saag 2016	0.800 (0.258, 2.484)	10/64	6/32
Sarris 2007	1.000 (0.000, Inf)	0/18	0/18
Schumacher 2008	0.570 (0.240, 1.356)	40/938	9/134
Segal 2015	0.137 (0.003, 6.919)	0/69	1/70
Separham 2016	0.235 (0.072, 0.764)	2/70	10/70
Shi 2012	0.907 (0.000, Inf)	0/21	0/19
Sircar 2015	1.000 (0.000, Inf)	0/54	0/54
Siu 2006	1.076 (0.000, Inf)	0/26	0/28
Szwejkowski 2013	0.135 (0.003, 6.820)	0/33	1/33
Taheraghdam 2014	0.487 (0.093, 2.567)	2/35	4/35
Takir 2015	0.826 (0.000, Inf)	0/40	0/33
Tan 2014	0.400 (0.158, 1.010)	7/57	15/56
Tanaka 2015	0.803 (0.000, Inf)	0/25	0/20
Tani 2015	1.000 (0.000, Inf)	0/30	0/30
Togha 2007	1.108 (0.000, Inf)	0/18	0/20
Tsuruta 2015	0.135 (0.003, 6.820)	0/27	1/27
Usharani 2016	0.946 (0.000, Inf)	0/18	0/17
Wang 2012	1.034 (0.000, Inf)	0/29	0/30
Wang 2015	0.977 (0.000, Inf)	0/43	0/42
Weiser 2012	1.016 (0.000, Inf)	0/123	0/125
Weiser 2014	1.000 (0.000, Inf)	0/90	0/90
Yin 2015	0.928 (0.000, Inf)	0/41	0/38
Zhang 2012	0.138 (0.058, 0.330)	11/40	31/40
Ziaee 2006	0.932 (0.000, Inf)	0/29	0/27
**Overall**	**0.656 (0.539, 0.799)**	**285/6675**	**280/4085**

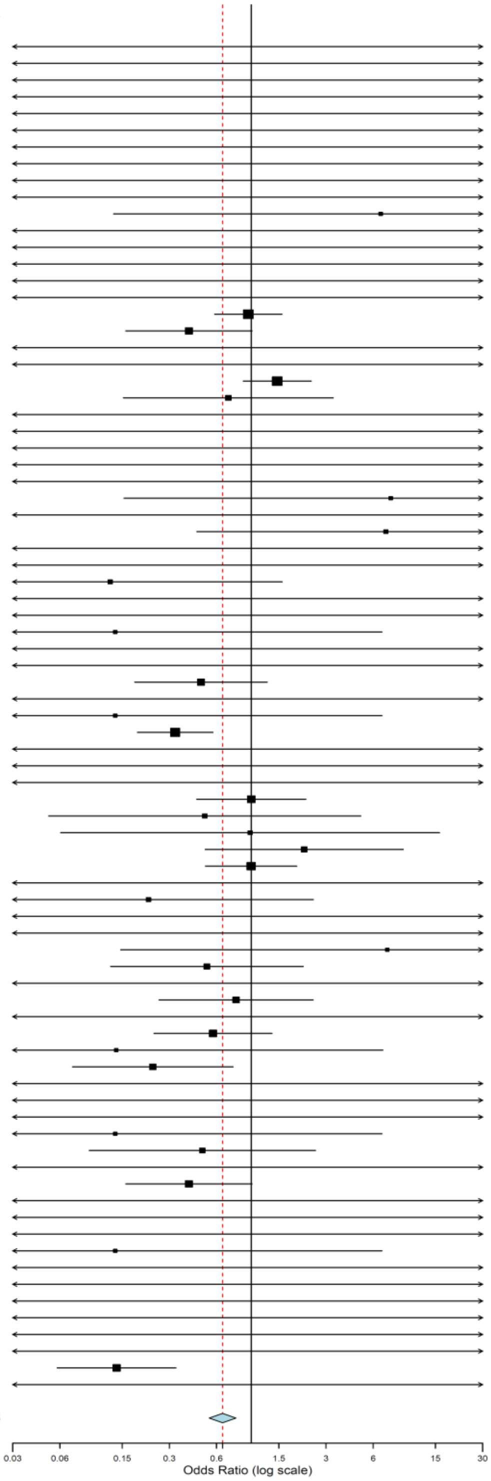

Favours XOI **Favours control**

Figure S9: Forest plot comparing the risk of total cardiovascular events between xanthine oxidase inhibitors and control. Numbers are Peto odds ratio and 95% CI. Heterogeneity: I^2=49%, Cochran´s Q test: P=0.002.

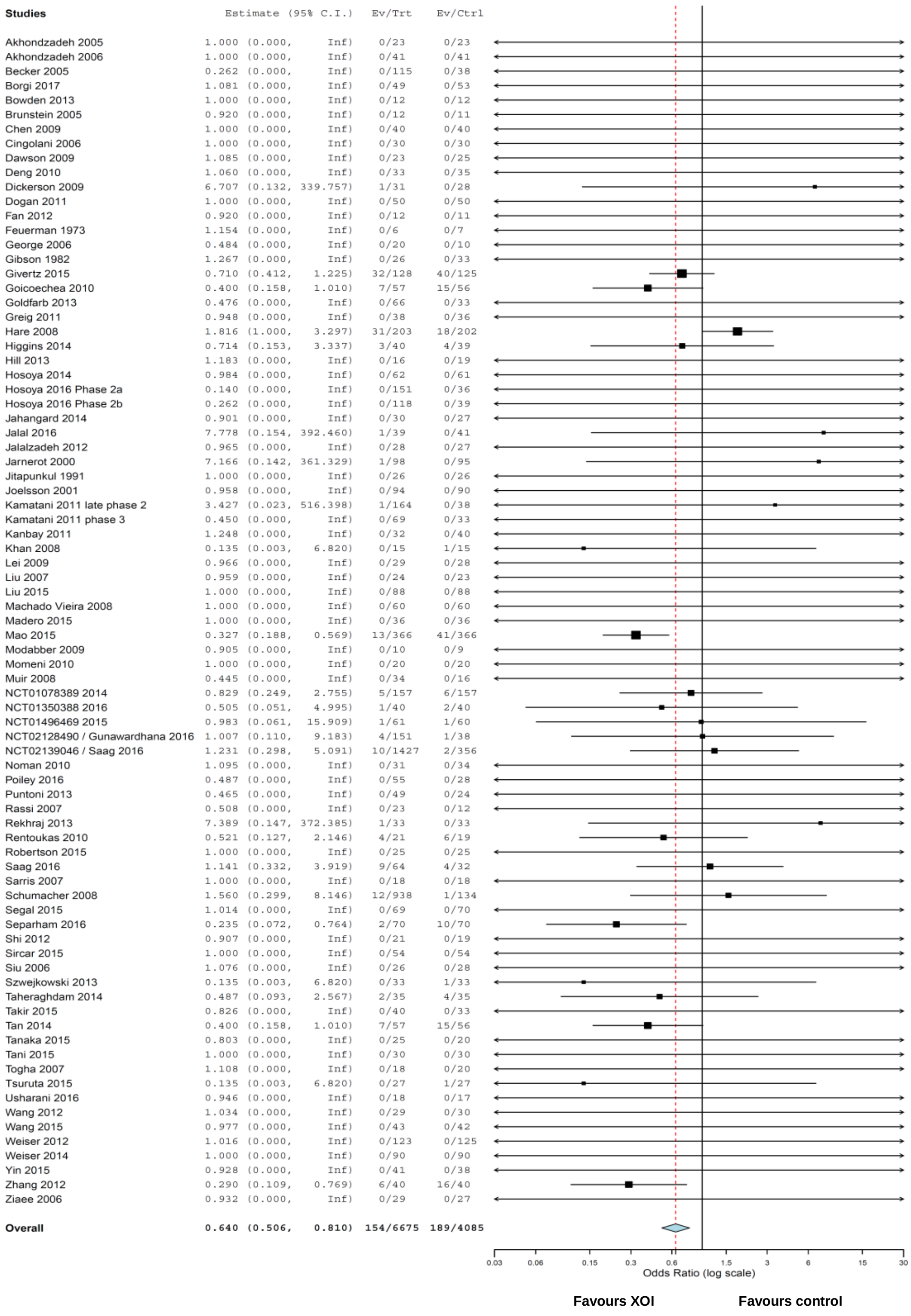

Figure S10: Forest plot comparing the risk of serious cardiovascular events between xanthine oxidase inhibitors and control. Numbers are Peto odds ratio and 95% CI. Heterogeneity: I^2=34%, Cochran´s Q test: P=0.050.

Studies	Estimate (95% C.I.)			Ev/Trt	Ev/Ctrl
Akhondzadeh 2005	1.000	(0.000,	Inf)	0/23	0/23
Akhondzadeh 2006	1.000	(0.000,	Inf)	0/41	0/41
Borgi 2017	1.081	(0.000,	Inf)	0/49	0/53
Bowden 2013	1.000	(0.000,	Inf)	0/12	0/12
Brunstein 2005	0.920	(0.000,	Inf)	0/12	0/11
Chen 2009	1.000	(0.000,	Inf)	0/40	0/40
Cingolani 2006	1.000	(0.000,	Inf)	0/30	0/30
Dawson 2009	1.085	(0.000,	Inf)	0/23	0/25
Deng 2010	1.060	(0.000,	Inf)	0/33	0/35
Dickerson 2009	6.707	(0.132,	339.757)	1/31	0/28
Dogan 2011	1.000	(0.000,	Inf)	0/50	0/50
Fan 2012	0.920	(0.000,	Inf)	0/12	0/11
Feuerman 1973	1.154	(0.000,	Inf)	0/6	0/7
George 2006	0.484	(0.000,	Inf)	0/20	0/10
Gibson 1982	1.267	(0.000,	Inf)	0/26	0/33
Givertz 2015	0.977	(0.000,	Inf)	0/128	0/125
Goldfarb 2013	1.000	(0.000,	Inf)	0/33	0/33
Greig 2011	0.948	(0.000,	Inf)	0/38	0/36
Higgins 2014	0.975	(0.060,	15.868)	1/40	1/39
Hill 2013	1.183	(0.000,	Inf)	0/16	0/19
Hosoya 2016 Phase 2b	1.000	(0.000,	Inf)	0/39	0/39
Jahangard 2014	0.901	(0.000,	Inf)	0/30	0/27
Jalalzadeh 2012	0.965	(0.000,	Inf)	0/28	0/27
Jarnerot 2000	0.970	(0.000,	Inf)	0/98	0/95
Jitapunkul 1991	1.000	(0.000,	Inf)	0/26	0/26
Joelsson 2001	0.958	(0.000,	Inf)	0/94	0/90
Kanbay 2011	1.248	(0.000,	Inf)	0/32	0/40
Khan 2008	1.000	(0.000,	Inf)	0/15	0/15
Lei 2009	0.966	(0.000,	Inf)	0/29	0/28
Liu 2007	0.959	(0.000,	Inf)	0/24	0/23
Liu 2015	1.000	(0.000,	Inf)	0/88	0/88
Machado Vieira 2008	1.000	(0.000,	Inf)	0/60	0/60
Madero 2015	1.000	(0.000,	Inf)	0/36	0/36
Mao 2015	0.135	(0.003,	6.820)	0/366	1/366
Modabber 2009	0.905	(0.000,	Inf)	0/10	0/9
Momeni 2010	1.000	(0.000,	Inf)	0/20	0/20
Muir 2008	0.445	(0.000,	Inf)	0/34	0/16
Noman 2010	1.095	(0.000,	Inf)	0/31	0/34
Parmley 1992	0.360	(0.050,	2.610)	1/71	3/71
Poiley 2016	0.487	(0.000,	Inf)	0/55	0/28
Puntoni 2013	0.465	(0.000,	Inf)	0/49	0/24
Rassi 2007	0.508	(0.000,	Inf)	0/23	0/12
Rekhraj 2013	1.000	(0.000,	Inf)	0/33	0/33
Robertson 2015	1.000	(0.000,	Inf)	0/25	0/25
Sarris 2007	1.000	(0.000,	Inf)	0/18	0/18
Schumacher 2008	0.473	(0.000,	Inf)	0/268	0/134
Segal 2015	1.014	(0.000,	Inf)	0/69	0/70
Shen 2010	1.000	(0.000,	Inf)	0/26	0/26
Shi 2012	0.907	(0.000,	Inf)	0/21	0/19
Siu 2006	1.076	(0.000,	Inf)	0/26	0/28
Szwejkowski 2013	1.000	(0.000,	Inf)	0/33	0/33
Taheraghdam 2014	0.352	(0.047,	2.608)	1/35	3/35
Takir 2015	0.826	(0.000,	Inf)	0/40	0/33
Togha 2007	1.108	(0.000,	Inf)	0/18	0/20
Wang 2012	1.034	(0.000,	Inf)	0/29	0/30
Wang 2015	0.977	(0.000,	Inf)	0/43	0/42
Weiser 2012	1.016	(0.000,	Inf)	0/123	0/125
Weiser 2014	1.000	(0.000,	Inf)	0/90	0/90
Yin 2015	0.928	(0.000,	Inf)	0/41	0/38
Zhang 2012	0.291	(0.098,	0.865)	4/40	12/40
Zhou 2009	0.922	(0.000,	Inf)	0/51	0/47
Ziaee 2006	0.932	(0.000,	Inf)	0/29	0/27
**Overall**	**0.376**	**(0.171,**	**0.827)**	**8/2979**	**20/2748**

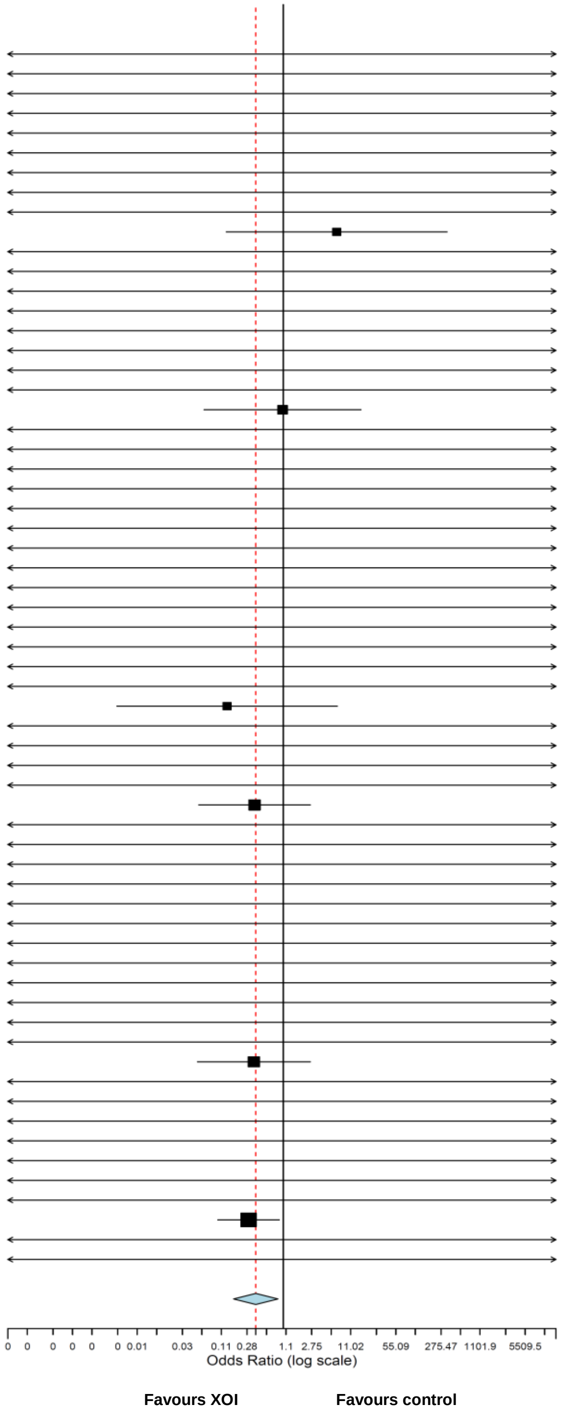

Figure S11: Forest plot comparing the risk of myocardial infarction/urgent revascularizatiom between allopurinol/oxypurinol and control. Numbers are Peto odds ratio and 95% CI. Heterogeneity: I^2=0%, Cochran's Q test: P=0.700.

Studies	Estimate (95% C.I.)	Ev/Trt	Ev/Ctrl
Akhondzadeh 2005	1.000 (0.000, Inf)	0/23	0/23
Akhondzadeh 2006	1.000 (0.000, Inf)	0/41	0/41
Borgi 2017	1.081 (0.000, Inf)	0/49	0/53
Bowden 2013	1.000 (0.000, Inf)	0/12	0/12
Brunstein 2005	0.920 (0.000, Inf)	0/12	0/11
Chen 2009	1.000 (0.000, Inf)	0/40	0/40
Cingolani 2006	1.000 (0.000, Inf)	0/30	0/30
Dawson 2009	1.085 (0.000, Inf)	0/23	0/25
Deng 2010	1.060 (0.000, Inf)	0/33	0/35
Dickerson 2009	0.905 (0.000, Inf)	0/31	0/28
Dogan 2011	1.000 (0.000, Inf)	0/50	0/50
Fan 2012	0.920 (0.000, Inf)	0/12	0/11
Feuerman 1973	1.154 (0.000, Inf)	0/6	0/7
George 2006	0.484 (0.000, Inf)	0/20	0/10
Gibson 1982	1.267 (0.000, Inf)	0/26	0/33
Givertz 2015	0.650 (0.111, 3.806)	2/128	3/125
Goldfarb 2013	1.000 (0.000, Inf)	0/33	0/33
Greig 2011	0.948 (0.000, Inf)	0/38	0/36
Higgins 2014	0.975 (0.000, Inf)	0/40	0/39
Hill 2013	1.183 (0.000, Inf)	0/16	0/19
Hosoya 2016 Phase 2b	1.000 (0.000, Inf)	0/39	0/39
Jahangard 2014	0.901 (0.000, Inf)	0/30	0/27
Jarnerot 2000	0.970 (0.000, Inf)	0/98	0/95
Jitapunkul 1991	1.000 (0.000, Inf)	0/26	0/26
Joelsson 2001	0.958 (0.000, Inf)	0/94	0/90
Kanbay 2011	1.248 (0.000, Inf)	0/32	0/40
Kao 2011	0.400 (0.084, 1.907)	2/30	5/31
Khan 2008	1.000 (0.000, Inf)	0/15	0/15
Lei 2009	0.966 (0.000, Inf)	0/29	0/28
Liu 2007	0.959 (0.000, Inf)	0/24	0/23
Liu 2015	0.478 (0.181, 1.264)	6/88	12/88
Machado Vieira 2008	1.000 (0.000, Inf)	0/60	0/60
Madero 2015	0.135 (0.003, 6.820)	0/36	1/36
Modabber 2009	0.905 (0.000, Inf)	0/10	0/9
Momeni 2010	1.000 (0.000, Inf)	0/20	0/20
Muir 2008	0.445 (0.000, Inf)	0/34	0/16
Noman 2010	1.095 (0.000, Inf)	0/31	0/34
Poiley 2016	0.221 (0.020, 2.492)	1/55	2/28
Puntoni 2013	0.465 (0.000, Inf)	0/49	0/24
Rassi 2007	0.508 (0.000, Inf)	0/23	0/12
Rekhraj 2013	1.000 (0.000, Inf)	0/33	0/33
Robertson 2015	1.000 (0.000, Inf)	0/25	0/25
Sarris 2007	1.000 (0.000, Inf)	0/18	0/18
Schumacher 2008	0.162 (0.046, 0.578)	3/268	8/134
Shi 2012	0.109 (0.011, 1.113)	0/21	3/19
Siu 2006	1.076 (0.000, Inf)	0/26	0/28
Szwejkowski 2013	1.000 (0.000, Inf)	0/33	0/33
Takir 2015	0.826 (0.000, Inf)	0/40	0/33
Togha 2007	1.108 (0.000, Inf)	0/18	0/20
Wang 2012	1.034 (0.000, Inf)	0/29	0/30
Wang 2015	0.977 (0.000, Inf)	0/43	0/42
Weiser 2012	1.016 (0.000, Inf)	0/123	0/125
Weiser 2014	1.000 (0.000, Inf)	0/90	0/90
Yin 2015	0.928 (0.000, Inf)	0/41	0/38
Ziaee 2006	0.932 (0.000, Inf)	0/29	0/27
**Overall**	**0.320 (0.177, 0.581)**	**14/2323**	**34/2097**

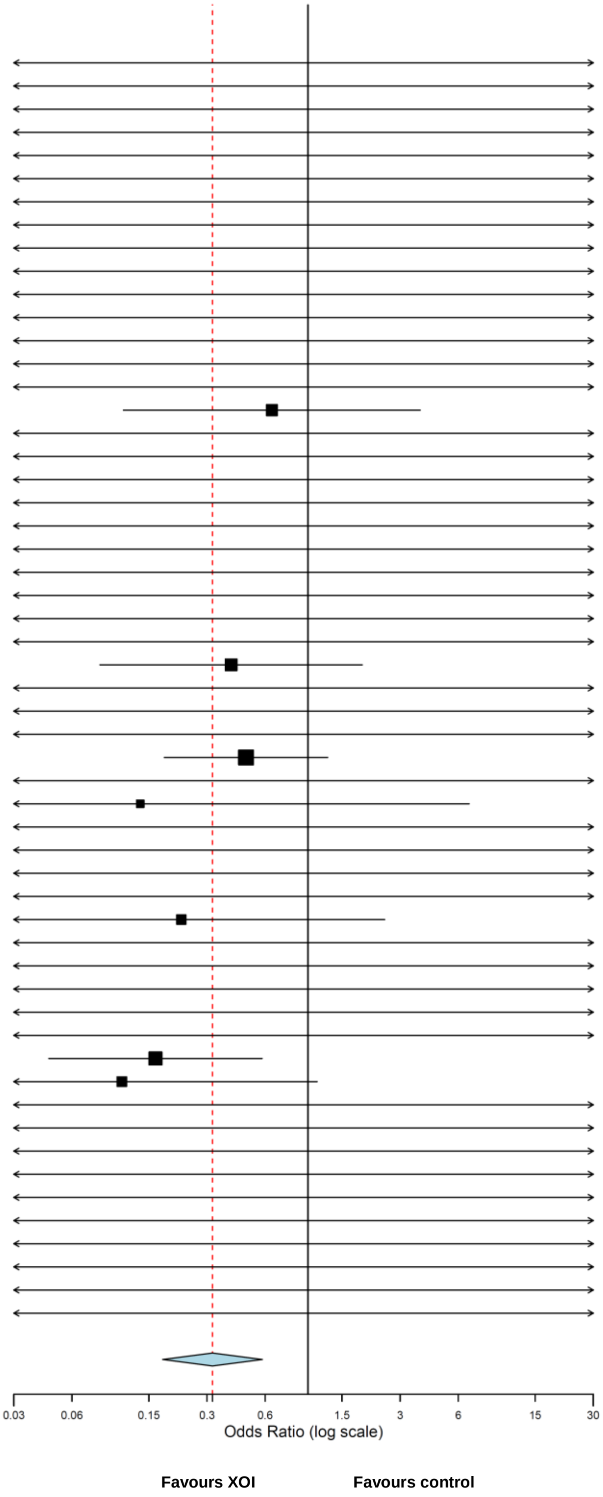

Figure S12: Forest plot comparing the risk of new/worsening hypertension between allopurinol/oxypurinol and control. Numbers are Peto odds ratio and 95% CI. Heterogeneity: I^2= 0%, Cochran´s Q test: P=0.737

Studies	Estimate (95% C.I.)	Ev/Trt	Ev/Ctrl
Akhondzadeh 2005	1.000 (0.000, Inf)	0/23	0/23
Akhondzadeh 2006	1.000 (0.000, Inf)	0/41	0/41
Borgi 2017	1.081 (0.000, Inf)	0/49	0/53
Bowden 2013	1.000 (0.000, Inf)	0/12	0/12
Brunstein 2005	0.920 (0.000, Inf)	0/12	0/11
Chen 2009	1.000 (0.000, Inf)	0/40	0/40
Cingolani 2006	1.000 (0.000, Inf)	0/30	0/30
Dawson 2009	1.085 (0.000, Inf)	0/23	0/25
Deng 2010	1.060 (0.000, Inf)	0/33	0/35
Dickerson 2009	0.905 (0.000, Inf)	0/31	0/28
Dogan 2011	1.000 (0.000, Inf)	0/50	0/50
Fan 2012	0.920 (0.000, Inf)	0/12	0/11
Feuerman 1973	1.154 (0.000, Inf)	0/6	0/7
George 2006	0.484 (0.000, Inf)	0/20	0/10
Gibson 1982	1.267 (0.000, Inf)	0/26	0/33
Givertz 2015	0.957 (0.584, 1.568)	58/128	58/125
Goldfarb 2013	1.000 (0.000, Inf)	0/33	0/33
Greig 2011	0.948 (0.000, Inf)	0/38	0/36
Hare 2008	1.465 (0.890, 2.411)	44/203	32/202
Higgins 2014	0.975 (0.000, Inf)	0/40	0/39
Hill 2013	1.183 (0.000, Inf)	0/16	0/19
Hosoya 2016 Phase 2b	1.000 (0.000, Inf)	0/39	0/39
Jahangard 2014	0.901 (0.000, Inf)	0/30	0/27
Jalalzadeh 2012	0.965 (0.000, Inf)	0/28	0/27
Jarnerot 2000	0.970 (0.000, Inf)	0/98	0/95
Jitapunkul 1991	1.000 (0.000, Inf)	0/26	0/26
Joelsson 2001	0.958 (0.000, Inf)	0/94	0/90
Kanbay 2011	1.248 (0.000, Inf)	0/32	0/40
Kao 2011	1.033 (0.000, Inf)	0/30	0/31
Khan 2008	1.000 (0.000, Inf)	0/15	0/15
Lei 2009	0.966 (0.000, Inf)	0/29	0/28
Liu 2007	0.959 (0.000, Inf)	0/24	0/23
Liu 2015	1.000 (0.000, Inf)	0/88	0/88
Machado Vieira 2008	1.000 (0.000, Inf)	0/60	0/60
Madero 2015	1.000 (0.000, Inf)	0/36	0/36
Modabber 2009	0.905 (0.000, Inf)	0/10	0/9
Momeni 2010	1.000 (0.000, Inf)	0/20	0/20
Muir 2008	0.445 (0.000, Inf)	0/34	0/16
Noman 2010	1.095 (0.000, Inf)	0/31	0/34
Poiley 2016	0.487 (0.000, Inf)	0/55	0/28
Puntoni 2013	0.465 (0.000, Inf)	0/49	0/24
Rassi 2007	0.508 (0.000, Inf)	0/23	0/12
Rekhraj 2013	1.000 (0.000, Inf)	0/33	0/33
Robertson 2015	1.000 (0.000, Inf)	0/25	0/25
Sarris 2007	1.000 (0.000, Inf)	0/18	0/18
Separham 2016	0.254 (0.074, 0.865)	2/70	9/70
Shi 2012	0.907 (0.000, Inf)	0/21	0/19
Siu 2006	1.076 (0.000, Inf)	0/26	0/28
Szwejkowski 2013	1.000 (0.000, Inf)	0/33	0/33
Taheraghdam 2014	0.135 (0.003, 6.820)	0/35	1/35
Takir 2015	0.826 (0.000, Inf)	0/40	0/33
Togha 2007	1.108 (0.000, Inf)	0/18	0/20
Wang 2012	1.034 (0.000, Inf)	0/29	0/30
Wang 2015	0.977 (0.000, Inf)	0/43	0/42
Weiser 2012	1.016 (0.000, Inf)	0/123	0/125
Weiser 2014	1.000 (0.000, Inf)	0/90	0/90
Yin 2015	0.928 (0.000, Inf)	0/41	0/38
Zhang 2012	0.268 (0.098, 0.733)	5/40	15/40
Ziaee 2006	0.932 (0.000, Inf)	0/29	0/27
**Overall**	**0.905 (0.658, 1.244)**	**109/2431**	**115/2337**

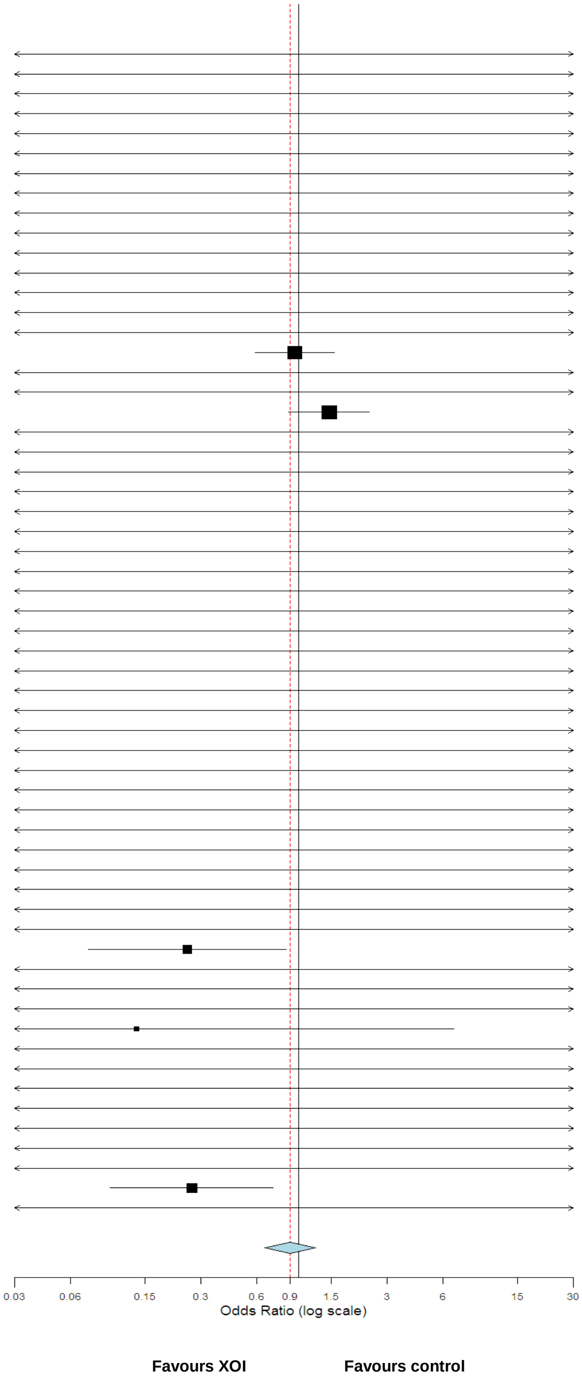

Figure S13: Forest plot comparing the risk of new/worsening heart failure between allopurinol/oxypurinol and control. Numbers are Peto odds ratio and 95% CI. Heterogeneity: I^2= 72%, Cochran´s Q test: P=0.006.

Studies	Estimate (95% C.I.)	Ev/Trt	Ev/Ctrl
Akhondzadeh 2005	1.000 (0.000, Inf)	0/23	0/23
Akhondzadeh 2006	1.000 (0.000, Inf)	0/41	0/41
Borgi 2017	1.081 (0.000, Inf)	0/49	0/53
Bowden 2013	1.000 (0.000, Inf)	0/12	0/12
Brunstein 2005	0.920 (0.000, Inf)	0/12	0/11
Chen 2009	1.000 (0.000, Inf)	0/40	0/40
Cingolani 2006	1.000 (0.000, Inf)	0/30	0/30
Dawson 2009	1.085 (0.000, Inf)	0/23	0/25
Deng 2010	1.060 (0.000, Inf)	0/33	0/35
Dickerson 2009	6.707 (0.132, 339.757)	1/31	0/28
Dogan 2011	1.000 (0.000, Inf)	0/50	0/50
Fan 2012	0.920 (0.000, Inf)	0/12	0/11
Feuerman 1973	1.154 (0.000, Inf)	0/6	0/7
George 2006	0.484 (0.000, Inf)	0/20	0/10
Gibson 1982	1.267 (0.000, Inf)	0/26	0/33
Givertz 2015	0.957 (0.584, 1.568)	58/128	58/125
Goicoechea 2010	0.400 (0.158, 1.010)	7/57	15/56
Goldfarb 2013	1.000 (0.000, Inf)	0/33	0/33
Greig 2011	0.948 (0.000, Inf)	0/38	0/36
Hare 2008	1.465 (0.890, 2.411)	44/203	32/202
Higgins 2014	0.714 (0.153, 3.337)	3/40	4/39
Hill 2013	1.183 (0.000, Inf)	0/16	0/19
Hosoya 2016 Phase 2b	1.000 (0.000, Inf)	0/39	0/39
Jahangard 2014	0.901 (0.000, Inf)	0/30	0/27
Jalal 2016	7.778 (0.154, 392.460)	1/39	0/41
Jalalzadeh 2012	0.965 (0.000, Inf)	0/28	0/27
Jarnerot 2000	7.241 (0.449, 116.640)	2/98	0/95
Jitapunkul 1991	1.000 (0.000, Inf)	0/26	0/26
Joelsson 2001	0.958 (0.000, Inf)	0/94	0/90
Kanbay 2011	1.248 (0.000, Inf)	0/32	0/40
Khan 2008	0.135 (0.003, 6.820)	0/15	1/15
Lei 2009	0.966 (0.000, Inf)	0/29	0/28
Liu 2007	0.959 (0.000, Inf)	0/24	0/23
Liu 2015	0.478 (0.181, 1.264)	6/88	12/88
Machado Vieira 2008	1.000 (0.000, Inf)	0/60	0/60
Madero 2015	0.135 (0.003, 6.820)	0/36	1/36
Mao 2015	0.327 (0.188, 0.569)	13/366	41/366
Modabber 2009	0.905 (0.000, Inf)	0/10	0/9
Momeni 2010	1.000 (0.000, Inf)	0/20	0/20
Muir 2008	0.445 (0.000, Inf)	0/34	0/16
Noman 2010	1.095 (0.000, Inf)	0/31	0/34
Poiley 2016	0.221 (0.020, 2.492)	1/55	2/28
Puntoni 2013	0.465 (0.000, Inf)	0/49	0/24
Rassi 2007	0.508 (0.000, Inf)	0/23	0/12
Rekhraj 2013	7.389 (0.147, 372.385)	1/33	0/33
Rentoukas 2010	0.521 (0.127, 2.146)	4/21	6/19
Robertson 2015	1.000 (0.000, Inf)	0/25	0/25
Sarris 2007	1.000 (0.000, Inf)	0/18	0/18
Schumacher 2008	0.189 (0.059, 0.610)	4/268	9/134
Segal 2015	0.137 (0.003, 6.919)	0/69	1/70
Separham 2016	0.235 (0.072, 0.764)	2/70	10/70
Shi 2012	0.907 (0.000, Inf)	0/21	0/19
Siu 2006	1.076 (0.000, Inf)	0/26	0/28
Szwejkowski 2013	0.135 (0.003, 6.820)	0/33	1/33
Taheraghdam 2014	0.487 (0.093, 2.567)	2/35	4/35
Takir 2015	0.826 (0.000, Inf)	0/40	0/33
Tan 2014	0.400 (0.158, 1.010)	7/57	15/56
Togha 2007	1.108 (0.000, Inf)	0/18	0/20
Wang 2012	1.034 (0.000, Inf)	0/29	0/30
Wang 2015	0.977 (0.000, Inf)	0/43	0/42
Weiser 2012	1.016 (0.000, Inf)	0/123	0/125
Weiser 2014	1.000 (0.000, Inf)	0/90	0/90
Yin 2015	0.928 (0.000, Inf)	0/41	0/38
Zhang 2012	0.138 (0.058, 0.330)	11/40	31/40
Ziaee 2006	0.932 (0.000, Inf)	0/29	0/27
**Overall**	**0.572 (0.457, 0.718)**	**167/3278**	**243/3048**

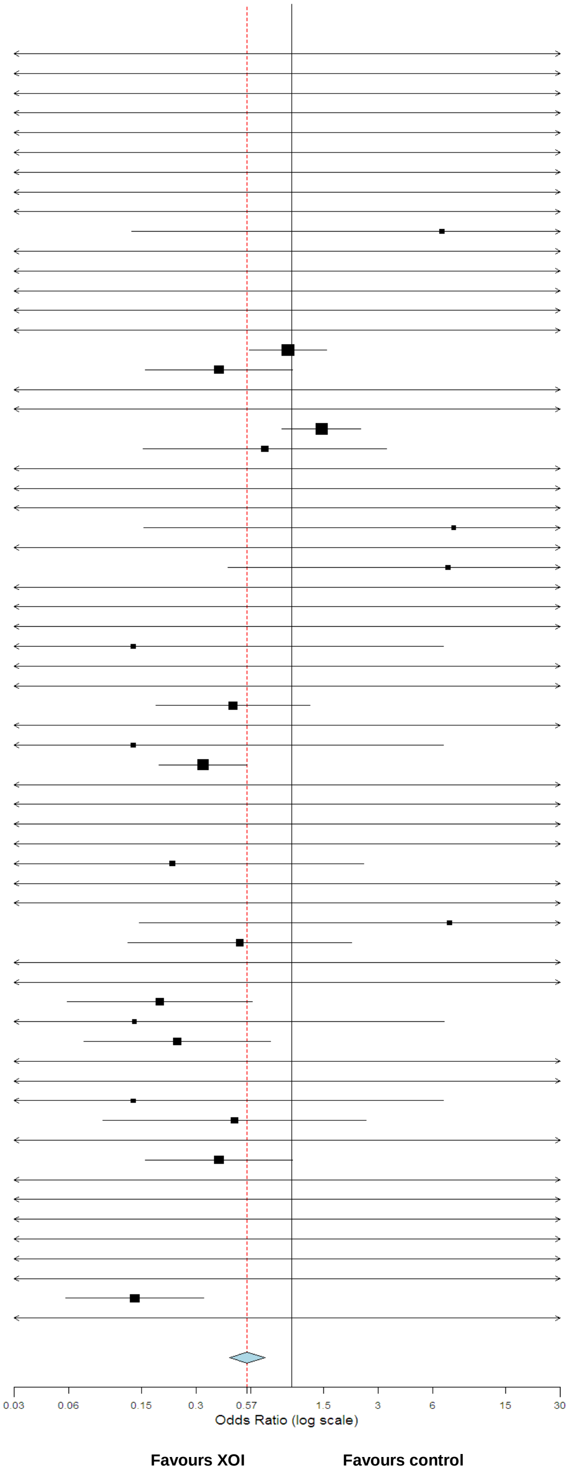

Figure S14: Forest plot comparing the risk of total cardiovascular events between allopurinol/oxypurinol and control. Numbers are Peto odds ratio and 95% CI. Heterogeneity: I^2= 60%, Cochran´s Q test: P<0.001.

Studies	Estimate (95% C.I.)	Ev/Trt	Ev/Ctrl
Akhondzadeh 2005	1.000 (0.000, Inf)	0/23	0/23
Akhondzadeh 2006	1.000 (0.000, Inf)	0/41	0/41
Borgi 2017	1.081 (0.000, Inf)	0/49	0/53
Bowden 2013	1.000 (0.000, Inf)	0/12	0/12
Brunstein 2005	0.920 (0.000, Inf)	0/12	0/11
Chen 2009	1.000 (0.000, Inf)	0/40	0/40
Cingolani 2006	1.000 (0.000, Inf)	0/30	0/30
Dawson 2009	1.085 (0.000, Inf)	0/23	0/25
Deng 2010	1.060 (0.000, Inf)	0/33	0/35
Dickerson 2009	6.707 (0.132, 339.757)	1/31	0/28
Dogan 2011	1.000 (0.000, Inf)	0/50	0/50
Fan 2012	0.920 (0.000, Inf)	0/12	0/11
Feuerman 1973	1.154 (0.000, Inf)	0/6	0/7
George 2006	0.484 (0.000, Inf)	0/20	0/10
Gibson 1982	1.267 (0.000, Inf)	0/26	0/33
Givertz 2015	0.710 (0.412, 1.225)	32/128	40/125
Goicoechea 2010	0.400 (0.158, 1.010)	7/57	15/56
Goldfarb 2013	1.000 (0.000, Inf)	0/33	0/33
Greig 2011	0.948 (0.000, Inf)	0/38	0/36
Hare 2008	1.816 (1.000, 3.297)	31/203	18/202
Higgins 2014	0.714 (0.153, 3.337)	3/40	4/39
Hill 2013	1.183 (0.000, Inf)	0/16	0/19
Hosoya 2016 Phase 2b	1.000 (0.000, Inf)	0/39	0/39
Jahangard 2014	0.901 (0.000, Inf)	0/30	0/27
Jalal 2016	7.778 (0.154, 392.460)	1/39	0/41
Jalalzadeh 2012	0.965 (0.000, Inf)	0/28	0/27
Jarnerot 2000	7.166 (0.142, 361.329)	1/98	0/95
Jitapunkul 1991	1.000 (0.000, Inf)	0/26	0/26
Joelsson 2001	0.958 (0.000, Inf)	0/94	0/90
Kanbay 2011	1.248 (0.000, Inf)	0/32	0/40
Khan 2008	0.135 (0.003, 6.820)	0/15	1/15
Lei 2009	0.966 (0.000, Inf)	0/29	0/28
Liu 2007	0.959 (0.000, Inf)	0/24	0/23
Liu 2015	1.000 (0.000, Inf)	0/88	0/88
Machado Vieira 2008	1.000 (0.000, Inf)	0/60	0/60
Madero 2015	1.000 (0.000, Inf)	0/36	0/36
Mao 2015	0.327 (0.188, 0.569)	13/366	41/366
Modabber 2009	0.905 (0.000, Inf)	0/10	0/9
Momeni 2010	1.000 (0.000, Inf)	0/20	0/20
Muir 2008	0.445 (0.000, Inf)	0/34	0/16
Noman 2010	1.095 (0.000, Inf)	0/31	0/34
Poiley 2016	0.487 (0.000, Inf)	0/55	0/28
Puntoni 2013	0.465 (0.000, Inf)	0/49	0/24
Rassi 2007	0.508 (0.000, Inf)	0/23	0/12
Rekhraj 2013	7.389 (0.147, 372.385)	1/33	0/33
Rentoukas 2010	0.521 (0.127, 2.146)	4/21	6/19
Robertson 2015	1.000 (0.000, Inf)	0/25	0/25
Sarris 2007	1.000 (0.000, Inf)	0/18	0/18
Schumacher 2008	0.471 (0.025, 8.951)	1/268	1/134
Segal 2015	1.014 (0.000, Inf)	0/69	0/70
Separham 2016	0.235 (0.072, 0.764)	2/70	10/70
Shi 2012	0.907 (0.000, Inf)	0/21	0/19
Siu 2006	1.076 (0.000, Inf)	0/26	0/28
Szwejkowski 2013	0.135 (0.003, 6.820)	0/33	1/33
Taheraghdam 2014	0.487 (0.093, 2.567)	2/35	4/35
Takir 2015	0.826 (0.000, Inf)	0/40	0/33
Tan 2014	0.400 (0.158, 1.010)	7/57	15/56
Togha 2007	1.108 (0.000, Inf)	0/18	0/20
Wang 2012	1.034 (0.000, Inf)	0/29	0/30
Wang 2015	0.977 (0.000, Inf)	0/43	0/42
Weiser 2012	1.016 (0.000, Inf)	0/123	0/125
Weiser 2014	1.000 (0.000, Inf)	0/90	0/90
Yin 2015	0.928 (0.000, Inf)	0/41	0/38
Zhang 2012	0.290 (0.109, 0.769)	6/40	16/40
Ziaee 2006	0.932 (0.000, Inf)	0/29	0/27
**Overall**	**0.587 (0.455, 0.758)**	**112/3278**	**172/3048**

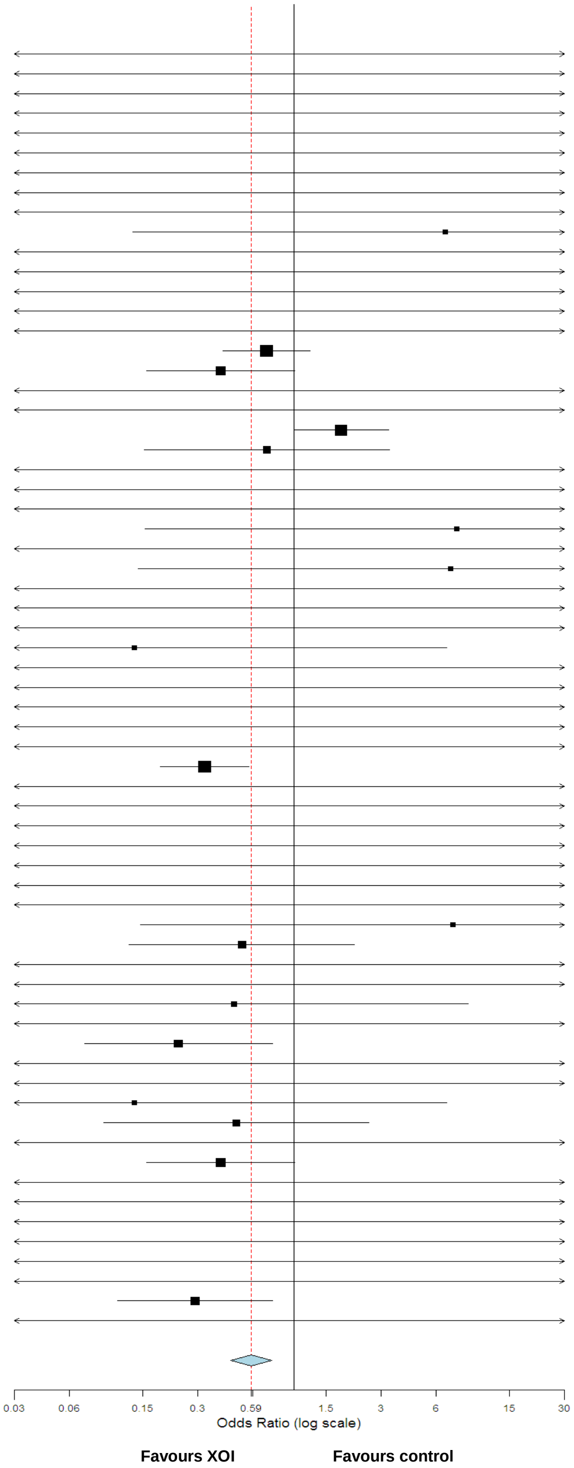

Figure S15: Forest plot comparing the risk of serious cardiovascular events between allopurinol/oxypurinol and control. Numbers are Peto odds ratio and 95% CI. Heterogeneity: I^2= 50%, Cochran´s Q test: P=0.011.

	Studies	Estimate (95% C.I.)	Ev/Trt	Ev/Ctrl
A	Gibson 1982	1.264 (0.024, 65.845)	0/26	0/33
	Goicoechea 2010	0.383 (0.143, 1.027)	7/57	15/56
	Hill 2015	1.182 (0.022, 62.893)	0/16	0/19
	Jalalzadeh 2012	0.965 (0.018, 50.353)	0/28	0/27
	Jarnerot 2000	4.948 (0.234, 104.432)	2/98	0/95
	Joelsson 2001	0.958 (0.019, 48.777)	0/94	0/90
	Momeni 2010	1.000 (0.019, 52.849)	0/20	0/20
	Muir 2008	0.478 (0.009, 25.178)	0/34	0/16
	Puntoni 2013	0.495 (0.010, 25.698)	0/49	0/24
	Rentoukas 2010	0.510 (0.119, 2.188)	4/21	6/19
	Shi 2012	0.907 (0.017, 47.938)	0/21	0/19
	Siu 2006	1.075 (0.021, 56.160)	0/26	0/28
	Taheraghdam 2014	0.470 (0.080, 2.749)	2/35	4/35
	Liu 2015	0.463 (0.166, 1.296)	6/88	12/88
	Deng 2010	1.060 (0.020, 54.940)	0/33	0/35
	Hosoya 2016 Phase 2b	1.000 (0.019, 51.659)	0/39	0/39
	Liu 2007	0.959 (0.018, 50.345)	0/24	0/23
	Sarris 2007	1.000 (0.019, 53.120)	0/18	0/18
	Separham 2016	0.176 (0.037, 0.838)	2/70	10/70
	Mao 2015	0.292 (0.154, 0.555)	13/366	41/366
	Lei 2009	0.966 (0.019, 50.355)	0/29	0/28
	Tan 2014	0.383 (0.143, 1.027)	7/57	15/56
	**Subgroup 1**	**0.387 (0.274, 0.547)**	**43/1249**	**103/1204**
B	Akhondzadeh 2005	1.000 (0.019, 52.531)	0/23	0/23
	Akhondzadeh 2006	1.000 (0.019, 51.597)	0/41	0/41
	Bowden 2013	1.000 (0.018, 54.465)	0/12	0/12
	Dawson 2009	1.085 (0.021, 56.910)	0/23	0/25
	Goldfarb 2013	1.000 (0.019, 51.887)	0/33	0/33
	Greig 2011	0.948 (0.018, 49.041)	0/38	0/36
	Higgins 2014	0.709 (0.148, 3.399)	3/40	4/39
	Jitapunkul 1991	1.000 (0.019, 52.286)	0/26	0/26
	Kanbay 2011	1.246 (0.024, 64.526)	0/32	0/40
	Khan 2008	0.312 (0.012, 8.285)	0/15	1/15
	Schumacher 2008	0.210 (0.064, 0.696)	4/268	9/134
	Weiser 2014	1.000 (0.020, 50.945)	0/90	0/90
	Ziaee 2006	0.932 (0.018, 48.618)	0/29	0/27
	Madero 2015	0.324 (0.013, 8.227)	0/36	1/36
	Takir 2015	0.827 (0.016, 42.811)	0/40	0/33
	Jalal 2016	3.234 (0.128, 81.791)	1/39	0/41
	Poiley 2016	0.241 (0.021, 2.778)	1/55	2/28
	Togha 2007	1.108 (0.021, 58.712)	0/18	0/20
	Wang 2012	1.034 (0.020, 53.829)	0/29	0/30
	Chen 2009	1.000 (0.019, 51.627)	0/40	0/40
	Zhang 2012	0.110 (0.040, 0.304)	11/40	31/40
	Wang 2015	0.977 (0.019, 50.370)	0/43	0/42
	**Subgroup 2**	**0.216 (0.119, 0.390)**	**20/1010**	**48/851**
C	Brunstein 2005	0.920 (0.017, 50.275)	0/12	0/11
	Dickerson 2009	2.803 (0.110, 71.656)	1/31	0/28
	Dogan 2011	1.000 (0.019, 51.382)	0/50	0/50
	Fan 2012	0.920 (0.017, 50.275)	0/12	0/11
	Feuerman 1973	1.154 (0.020, 66.773)	0/6	0/7
	Jahangard 2014	0.902 (0.017, 46.999)	0/30	0/27
	Machado Vieira 2008	1.000 (0.020, 51.218)	0/60	0/60
	Modabber 2009	0.905 (0.016, 50.244)	0/10	0/9
	Noman 2010	1.095 (0.021, 56.857)	0/31	0/34
	Rassi 2007	0.532 (0.010, 28.453)	0/23	0/12
	Rekhraj 2013	3.092 (0.121, 78.704)	1/33	0/33
	Szwejkowski 2013	0.323 (0.013, 8.231)	0/33	1/33
	Weiser 2012	1.016 (0.020, 51.617)	0/123	0/125
	Givertz 2015	0.957 (0.584, 1.570)	58/128	58/125
	Robertson 2015	1.000 (0.019, 52.362)	0/25	0/25
	Segal 2015	0.333 (0.013, 8.325)	0/69	1/70
	Borgi 2017	1.081 (0.021, 55.515)	0/49	0/53
	Yin 2015	0.928 (0.018, 47.911)	0/41	0/38
	George 2006	0.512 (0.009, 27.683)	0/20	0/10
	**Subgroup 3**	**0.959 (0.594, 1.547)**	**60/786**	**60/761**
	**Overall**	**0.449 (0.348, 0.578)**	**123/3045**	**211/2816**

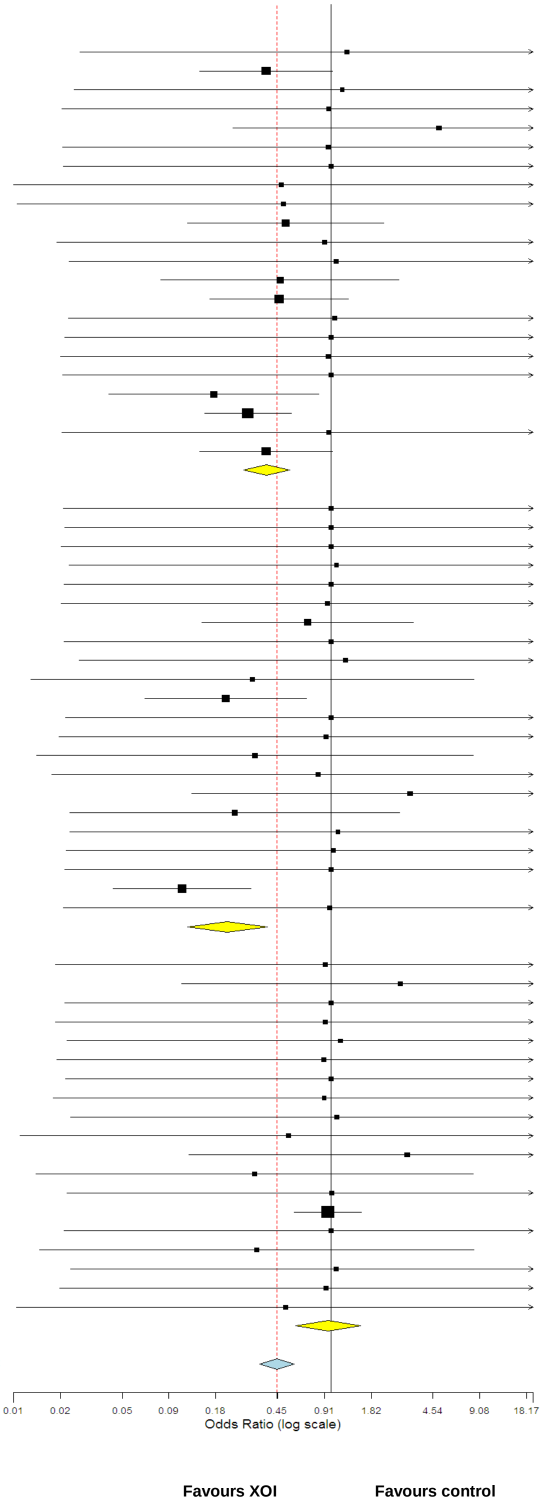

Figure S16: Forest plot comparing the risk of total cardiovascular events of low-dose (A), standard-dose (B), and high-dose of allopurinol (C) versus control.

	Studies	Estimate (95% C.I.)	Ev/Trt	Ev/Ctrl
	Gibson 1982	1.264 (0.024, 65.845)	0/26	0/33
	Goicoechea 2010	0.383 (0.143, 1.027)	7/57	15/56
A	Hill 2015	1.182 (0.022, 62.893)	0/16	0/19
	Jalalzadeh 2012	0.965 (0.018, 50.353)	0/28	0/27
	Jarnerot 2000	2.938 (0.118, 73.033)	1/98	0/95
	Joelsson 2001	0.958 (0.019, 48.777)	0/94	0/90
	Momeni 2010	1.000 (0.019, 52.849)	0/20	0/20
	Muir 2008	0.478 (0.009, 25.178)	0/34	0/16
	Puntoni 2013	0.495 (0.010, 25.698)	0/49	0/24
	Rentoukas 2010	0.510 (0.119, 2.188)	4/21	6/19
	Shi 2012	0.907 (0.017, 47.938)	0/21	0/19
	Siu 2006	1.075 (0.021, 56.160)	0/26	0/28
	Taheraghdam 2014	0.470 (0.080, 2.749)	2/35	4/35
	Liu 2015	1.000 (0.020, 50.957)	0/88	0/88
	Deng 2010	1.060 (0.020, 54.940)	0/33	0/35
	Hosoya 2016 Phase 2b	1.000 (0.019, 51.659)	0/39	0/39
	Liu 2007	0.959 (0.018, 50.345)	0/24	0/23
	Sarris 2007	1.000 (0.019, 53.120)	0/18	0/18
	Separham 2016	0.176 (0.037, 0.838)	2/70	10/70
	Mao 2015	0.292 (0.154, 0.555)	13/366	41/366
	Lei 2009	0.966 (0.019, 50.355)	0/29	0/28
	Tan 2014	0.383 (0.143, 1.027)	7/57	15/56
	**Subgroup 1**	**0.366 (0.252, 0.530)**	**36/1249**	**91/1204**
	Akhondzadeh 2005	1.000 (0.019, 52.531)	0/23	0/23
	Akhondzadeh 2006	1.000 (0.019, 51.597)	0/41	0/41
B	Bowden 2013	1.000 (0.018, 54.465)	0/12	0/12
	Dawson 2009	1.085 (0.021, 56.910)	0/23	0/25
	Goldfarb 2013	1.000 (0.019, 51.887)	0/33	0/33
	Greig 2011	0.948 (0.018, 49.041)	0/38	0/36
	Higgins 2014	0.709 (0.148, 3.399)	3/40	4/39
	Jitapunkul 1991	1.000 (0.019, 52.286)	0/26	0/26
	Kanbay 2011	1.246 (0.024, 64.526)	0/32	0/40
	Khan 2008	0.312 (0.012, 8.285)	0/15	1/15
	Schumacher 2008	0.498 (0.031, 8.026)	1/268	1/134
	Weiser 2014	1.000 (0.020, 50.945)	0/90	0/90
	Ziaee 2006	0.932 (0.018, 48.618)	0/29	0/27
	Madero 2015	1.000 (0.019, 51.764)	0/36	0/36
	Takir 2015	0.827 (0.016, 42.811)	0/40	0/33
	Jalal 2016	3.234 (0.128, 81.791)	1/39	0/41
	Poiley 2016	0.514 (0.010, 26.559)	0/55	0/28
	Togha 2007	1.108 (0.021, 58.712)	0/18	0/20
	Wang 2012	1.034 (0.020, 53.829)	0/29	0/30
	Chen 2009	1.000 (0.019, 51.627)	0/40	0/40
	Zhang 2012	0.265 (0.090, 0.775)	6/40	16/40
	Wang 2015	0.977 (0.019, 50.370)	0/43	0/42
	**Subgroup 2**	**0.411 (0.192, 0.882)**	**11/1010**	**22/851**
	Brunstein 2005	0.920 (0.017, 50.275)	0/12	0/11
	Dickerson 2009	2.803 (0.110, 71.656)	1/31	0/28
C	Dogan 2011	1.000 (0.019, 51.382)	0/50	0/50
	Fan 2012	0.920 (0.017, 50.275)	0/12	0/11
	Feuerman 1973	1.154 (0.020, 66.773)	0/6	0/7
	Jahangard 2014	0.902 (0.017, 46.999)	0/30	0/27
	Machado Vieira 2008	1.000 (0.020, 51.218)	0/60	0/60
	Modabber 2009	0.905 (0.016, 50.244)	0/10	0/9
	Noman 2010	1.095 (0.021, 56.857)	0/31	0/34
	Rassi 2007	0.532 (0.010, 28.453)	0/23	0/12
	Rekhraj 2013	3.092 (0.121, 78.704)	1/33	0/33
	Szwejkowski 2013	0.323 (0.013, 8.231)	0/33	1/33
	Weiser 2012	1.016 (0.020, 51.617)	0/123	0/125
	Givertz 2015	0.708 (0.409, 1.226)	32/128	40/125
	Robertson 2015	1.000 (0.019, 52.362)	0/25	0/25
	Segal 2015	1.014 (0.020, 51.841)	0/69	0/70
	Borgi 2017	1.081 (0.021, 55.515)	0/49	0/53
	Yin 2015	0.928 (0.018, 47.911)	0/41	0/38
	George 2006	0.512 (0.009, 27.683)	0/20	0/10
	**Subgroup 3**	**0.749 (0.441, 1.273)**	**34/786**	**41/761**
	**Overall**	**0.456 (0.343, 0.605)**	**81/3045**	**154/2816**

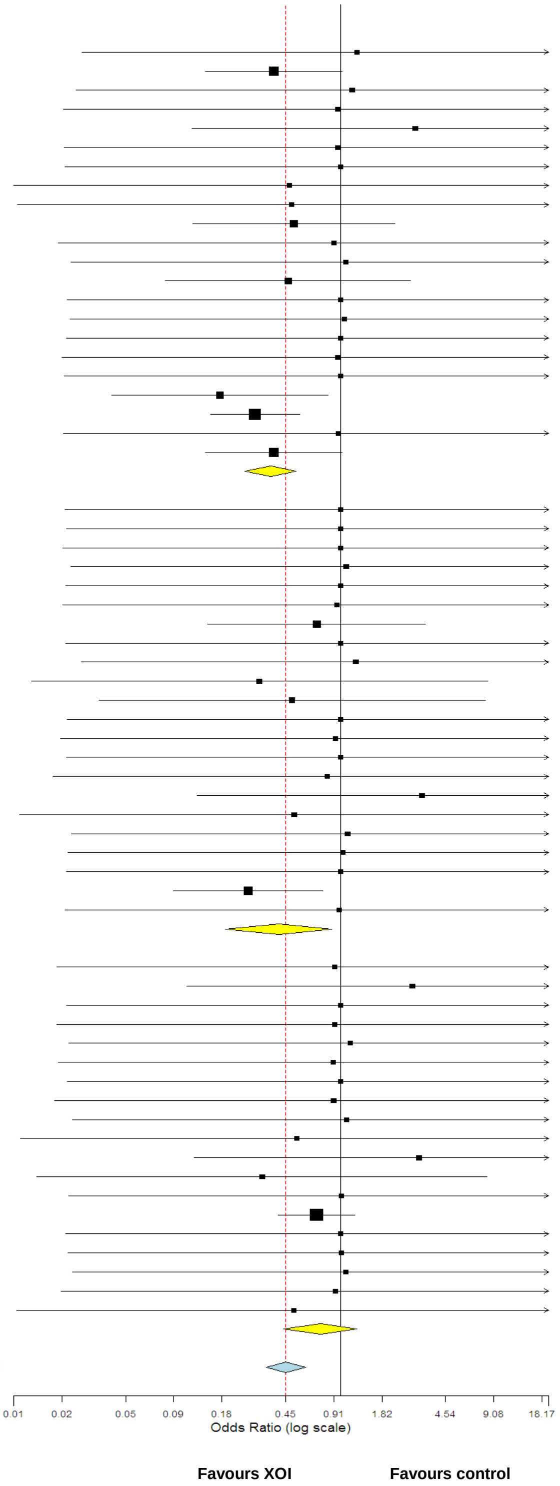

Figure S17: Forest plot comparing the risk of serious cardiovascular events of low-dose (A), standard-dose (B), and high-dose of allopurinol (C) versus control.

	Studies	Estimate (95% C.I.)	Ev/Trt	Ev/Ctrl
	Gibson 1982	1.264 (0.024, 65.845)	0/26	0/33
	Hill 2015	1.182 (0.022, 62.893)	0/16	0/19
	Jalalzadeh 2012	0.965 (0.018, 50.353)	0/28	0/27
A	Jarnerot 2000	0.970 (0.019, 49.358)	0/98	0/95
	Joelsson 2001	0.958 (0.019, 48.777)	0/94	0/90
	Momeni 2010	1.000 (0.019, 52.849)	0/20	0/20
	Muir 2008	0.478 (0.009, 25.178)	0/34	0/16
	Puntoni 2013	0.495 (0.010, 25.698)	0/49	0/24
	Shi 2012	0.907 (0.017, 47.938)	0/21	0/19
	Siu 2006	1.075 (0.021, 56.160)	0/26	0/28
	Taheraghdam 2014	0.324 (0.013, 8.228)	0/35	1/35
	Liu 2015	1.000 (0.020, 50.957)	0/88	0/88
	Deng 2010	1.060 (0.020, 54.940)	0/33	0/35
	Hosoya 2016 Phase 2b	1.000 (0.019, 51.659)	0/39	0/39
	Liu 2007	0.959 (0.018, 50.345)	0/24	0/23
	Sarris 2007	1.000 (0.019, 53.120)	0/18	0/18
	Separham 2016	0.199 (0.041, 0.959)	2/70	9/70
	Lei 2009	0.966 (0.019, 50.355)	0/29	0/28
	**Subgroup 1**	**0.240 (0.074, 0.774)**	**2/748**	**10/707**
	Akhondzadeh 2005	1.000 (0.019, 52.531)	0/23	0/23
	Akhondzadeh 2006	1.000 (0.019, 51.597)	0/41	0/41
	Bowden 2013	1.000 (0.018, 54.465)	0/12	0/12
B	Dawson 2009	1.085 (0.021, 56.910)	0/23	0/25
	Goldfarb 2013	1.000 (0.019, 51.887)	0/33	0/33
	Greig 2011	0.948 (0.018, 49.041)	0/38	0/36
	Higgins 2014	0.975 (0.019, 50.368)	0/40	0/39
	Jitapunkul 1991	1.000 (0.019, 52.286)	0/26	0/26
	Kanbay 2011	1.246 (0.024, 64.526)	0/32	0/40
	Kao 2011	1.033 (0.020, 53.714)	0/30	0/31
	Khan 2008	1.000 (0.019, 53.659)	0/15	0/15
	Weiser 2014	1.000 (0.020, 50.945)	0/90	0/90
	Ziaee 2006	0.932 (0.018, 48.618)	0/29	0/27
	Madero 2015	1.000 (0.019, 51.764)	0/36	0/36
	Takir 2015	0.827 (0.016, 42.811)	0/40	0/33
	Poiley 2016	0.514 (0.010, 26.559)	0/55	0/28
	Togha 2007	1.108 (0.021, 58.712)	0/18	0/20
	Wang 2012	1.034 (0.020, 53.829)	0/29	0/30
	Chen 2009	1.000 (0.019, 51.627)	0/40	0/40
	Zhang 2012	0.238 (0.077, 0.741)	5/40	15/40
	Wang 2015	0.977 (0.019, 50.370)	0/43	0/42
	**Subgroup 2**	**0.268 (0.098, 0.733)**	**5/733**	**15/707**
	Brunstein 2005	0.920 (0.017, 50.275)	0/12	0/11
	Dickerson 2009	0.905 (0.017, 47.109)	0/31	0/28
	Dogan 2011	1.000 (0.019, 51.382)	0/50	0/50
C	Fan 2012	0.920 (0.017, 50.275)	0/12	0/11
	Feuerman 1973	1.154 (0.020, 66.773)	0/6	0/7
	Jahangard 2014	0.902 (0.017, 46.999)	0/30	0/27
	Machado Vieira 2008	1.000 (0.020, 51.218)	0/60	0/60
	Modabber 2009	0.905 (0.016, 50.244)	0/10	0/9
	Noman 2010	1.095 (0.021, 56.857)	0/31	0/34
	Rassi 2007	0.532 (0.010, 28.453)	0/23	0/12
	Rekhraj 2013	1.000 (0.019, 51.887)	0/33	0/33
	Szwejkowski 2013	1.000 (0.019, 51.887)	0/33	0/33
	Weiser 2012	1.016 (0.020, 51.617)	0/123	0/125
	Givertz 2015	0.957 (0.584, 1.570)	58/128	58/125
	Robertson 2015	1.000 (0.019, 52.362)	0/25	0/25
	Borgi 2017	1.081 (0.021, 55.515)	0/49	0/53
	Yin 2015	0.928 (0.018, 47.911)	0/41	0/38
	George 2006	0.512 (0.009, 27.683)	0/20	0/10
	**Subgroup 3**	**0.957 (0.584, 1.568)**	**58/717**	**58/691**
	**Overall**	**0.648 (0.428, 0.981)**	**65/2198**	**83/2105**

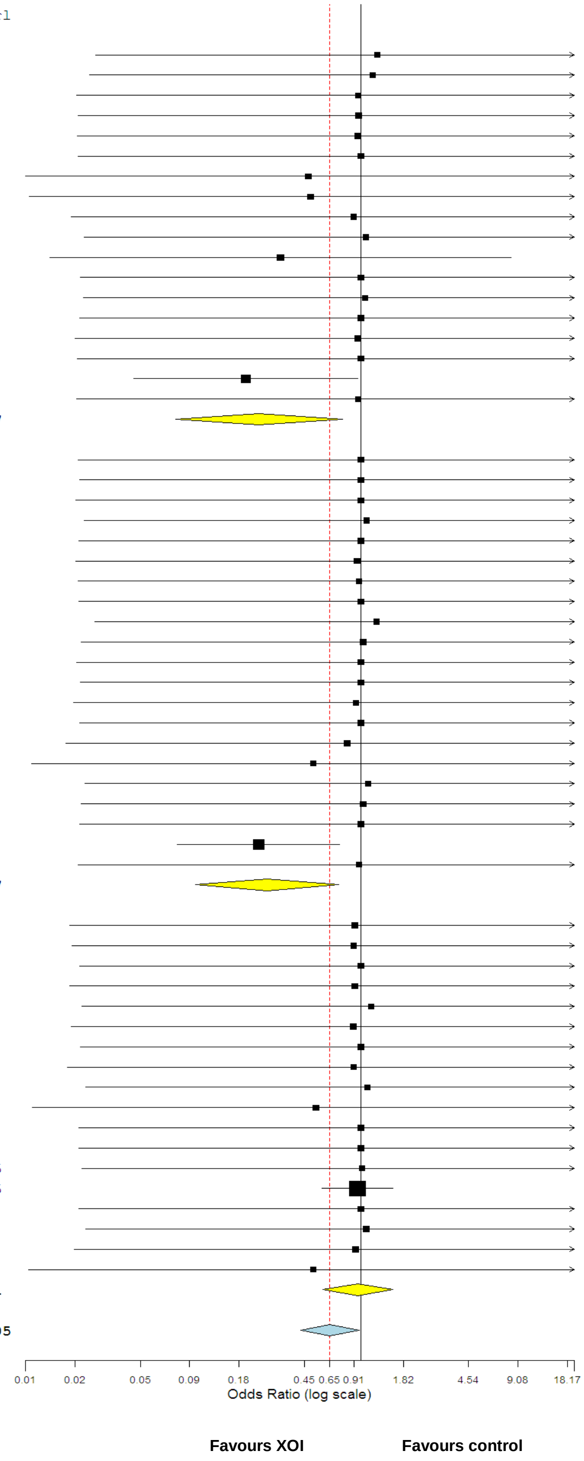

Figure S18: Forest plot comparing the risk of new/worsening heart failure of low-dose (A), standard-dose (B), and high-dose of allopurinol (C) versus control.

Table S2: Subgroup analyses of MACE and secondary outcomes according to the risk of bias.

**Outcomes**	Low risk of bias	Unknown risk of bias	High risk of bias
	OR_P (95% CI), number of studies*	OR_P (95% CI), number of studies*	OR_P (95% CI), number of studies*
**All studies**			
Major cardiovascular events	0.64 (0.28 to 1.44), 3 studies	1.05 (0.58 to 1.89), 16 studies	0.26 (0.09 to 0.78), 1 study
Hypertension	0.50 (0.10 to 2.50), 2 studies	0.60 (0.38 to 0.94), 9 studies	0.38 (0.16 to 0.94), 2 studies
Total cardiovascular outcomes	0.87 (0.55 to 1.36), 4 studies	0.76 (0.59 to 0.97), 20 studies	0.31 (0.19 to 0.48), 5 studies
Serious cardiovascular outcomes	0.69 (0.42 to 1.12), 3 studies	0.76 (0.55 to 1.03), 18 studies	0.36 (0.21 to 0.61), 4 studies
**Allopurinol/ oxypurinol studies**			
Major cardiovascular events	0.64 (0.28 to 1.44), 3 studies	1.03 (0.50 to 2.10), 8 studies	0.26 (0.09 to 0.78), 1 study
Myocardial infarction or urgent revascularization	0.50 (0.10 to 2.53), 2 studies	0.95 (0.06 to 15.22), 2 studies	0.31 (0.12 to 0.79), 2 studies
Hypertension	0.50 (0.10 to 2.50), 2 studies	0.23 (0.09 to 0.58), 3 studies	0.38 (0.16 to 0.94), 2 studies

Total cardiovascular outcomes	0.87 (0.55 to 1.36), 4 studies	0.62 (0.45 to 0.86), 13 studies	0.31 (0.20 to 0.49), 4 studies
Serious cardiovascular outcomes	0.69 (0.42 to 1.12), 3 studies	0.67 (0.47 to 0.95), 11 studies	0.36 (0.21 to 0.62), 3 studies
* Number of studies contributing with events. OR_P: Peto odds ratio; CI: confidence interval.			

**Table S3: Sensitivity analyses excluding from analyses studies at high risk of bias.**	
**Outcomes**	Low or unknown risk of bias
	OR_P (95% CI), P value, I^2 (P Value), number of studies
**All studies**	
Major adverse cardiovascular events (MACE)	0.88 (0.55 to 1.42), P=0.608, I^2 = 0% (P=0.509), 59 studies
Hypertension	0.59 (0.38 to 0.91), P=0.018, I^2 = 0% (P=0.497), 53 studies
Total cardiovascular outcomes	0.78 (0.63 to 0.97), P=0.026, I^2 = 37% (P=0.035), 59 studies. D-L: 0.75 (0.56 to 1.01), P=0.055, I^2=23% (P=0.155)
Serious cardiovascular events	0.74 (0.57 to 0.96), P=0.021, I^2=34% (P=0.067), 59 studies; D-L: 0.760 (0.53 to 1.09), P=0.141, I^2=22% (P=0.183)
**Allopurinol/ Oxypurinol studies**	
Major cardiovascular events	0.84 (0.49 to 1.43), P=0.512, I^2= 0% (P=0.565), 46 studies
Myocardial Infarction or urgent revascularization	0.59 (0.14 to 2.39), P=0.457, I^2=0% (0.663), 42 studies
Hypertension	0.28 (0.13 to 0.62), P=0.002, I^2=0% (0.745), 40 studies

Total cardiovascular outcomes	0.70 (0.54 to 0.90), P=0.006, I^2=56% (P=0.003), 46 studies; D-L: 0.60 (0.36 to 0.97), P=0.038, I^2=46% (P=0.019)
Serious cardiovascular outcomes	0.67 (0.50 to 0.90), P=0.007, I^2= 53% (P=0.011), 46 studies; D-L: 0.67 (0.39 to 1.14), P=0.141, I^2=45% (P=0.035)
OR_P: Peto odds ratio, except when indicted otherwise; CI: confidence interval; I^2: statistic of heterogeneity (P value of Cochran’s Q test); D-L: DerSimonian and Laird random effects odds ratio with zero-cell continuity correction.	

Supplementary text 7: Additional information on sensitivity analysis.

We also performed sensitivity analysis removing studies at high-risk of bias from the meta-regression analysis. There was still a statistical trend for association of increasing dose of allopurinol with higher risk of heart failure (P=0.051; Figure S19A, Additional file 1), and significant associations with total CV events (P=0.002; Figure S19B), and serious CV events (P=0.043; Figure S19C) were observed. Still analyzing only studies at low or unknown risk of bias, lower doses of allopurinol were associated with lower incidences of total CV events (low-dose: OR_P=0.36, 95% CI 0.23 to 0.57, P<0.001, I^2= 27% [Cochran's Q test, P=0.241]; standard dose: 0.32, 0.14 to 0.71, P=0.006, I^2=0% [P=0.439]; high-dose: 0.959, 0.594 to 1.55, P=0.862, I^2=0% [P=0.421]), serious CV events (low-dose: 0.35, 0.22 to 0.55, P<0.001, I^2=0% [P=0.520]; standard dose: 0.71, 0.21 to 2.43, P=589, I^2=0% [P=0.533]; high-dose: 0.75, 0.44 to 1.27, P=0.285, I^2=8% [P=0.351]), and also heart failure (low-dose: 0.22, 0.05 to 0.90, P=0.035; I^2= 0% [P=0.791]; standard dose: no studies; high-dose: 0.96, 0.58 to 1.57, P=0.862, I^2= not applicable).

We observed that studies at high-risk of bias used significantly lower doses than other studies (P<0.05 by Mann-Whitney test). Therefore, in an alternative sensitivity analysis, we included risk of bias (dichotomized as low/unknown and high) as a covariate in multivariate meta-regression testing the association of dose of allopurinol with CV outcomes. Dose maintained statistical trends for associations of with higher risk of total CV events (P=0.053), serious CV events (P=0.085), and heart failure (P=0.051), while risk of bias lost its significant association with these variables (P>0.400 in all tests).

We performed an additional sensitivity analysis on results for purine-like XOI (allopurinol and oxypurinol) excluding 15 studies published in non-English language. The results are the following:

- MACE: OR_P=0.86, 95% CI 0.50 to 1.49, P=0.600, I^2= 0% [P=0.554];
- myocardial infarction: 0.50, 0.13 to 1.87, P=0.302, I^2= 0% [P=0.386];
- hypertension: 0.32, 0.18 to 0.58, P<0.001, I^2= 0% [P=0.737];
- heart failure: 1.04, 0.74 to 1.45, P=0.835, I^2= 63% [P=0.045]; DerSimonian and Laird OR= 0.82, 0.41 to 1.65, P=0.587;
- total CV events: 0.77, 0.59 to 1.01, P=0.057, I^2= 44% [P=0.024]; DerSimonian and Laird OR= 0.60, 0.38 to 0.96, P=0.032;
- serious CV events: 0.80, 0.58 to 1.11, P=0.181, I^2= 38.1% [P=0.073]; DerSimonian and Laird OR= 0.71, 0.43 to 1.20, P=0.208.

Concerning meta-regression analyses of allopurinol dose versus effect, the results excluding non-English language studies are the following:

- heart failure: meta-regression coefficient= 0.003, 95% CI -0.000 to 0.007, P=0.051;
- total CV events: 0.002, 0.0005 to 0.004, P=0.010;
- serious CV events: 0.002, -0.0004 to 0.003, P=0.125.

So, including only studies published in English, there would still be significant protection for hypertension and total CV events, and the association of higher dose of allopurinol with risk of total CV events also kept statistical significance.

In another sensitivity analysis on the effects of purine-like XOI, we excluded studies whose duration was inferior to 90 days (the median duration of the studies included in the meta-analysis). The results for MACE (ORp=0.68, 95% CI 0.40 to 1.17, P=0.164, I^2=29.5%), myocardial infarction (ORp=0.30, 0.13 to 0.70, P=0.005, I^2=0%), total CV

events (ORp=0.60, 0.47 to 0.76, P<0.001, I^2=68.4%; DerSimonian and Laird [D-L] OR: 0.52, 0.31 to 0.86, P=0.011; see Figure S22), serious CV events (ORp=0.62, 0.47 to 0.80, P<0.001, I^2=55.9%; D-L OR= 0.61, 0.37 to 1.00, P=0.048), and hypertension (ORp=0.33, 95% CI 0.18 to 0.60, P<0.001, I^2=0%) were very similar to those obtained with the entire set of studies. However, excluding studies lasting less than 180 days, the results suggesting protection for MACE (OR_P=0.48, 0.25 to 0.92, P=0.027, I^2=0.0%; see Figure S23), myocardial infarction (ORp=0.27, 0.10 to 0.79, P=0.016, I^2=0%), total CV events (ORp =0.46, 0.35 to 0.60, P<0.001, I^2=62%; D-L OR= 0.43, 0.26 to 0.73, P=0.002), serious CV events (OR_P=0.46, 0.34 to 0.62, P<0.001, I^2=4.6%), and hypertension (OR_P=0.33, 0.18 to 0.62, P<0.001, I^2=0.0%) were even stronger.

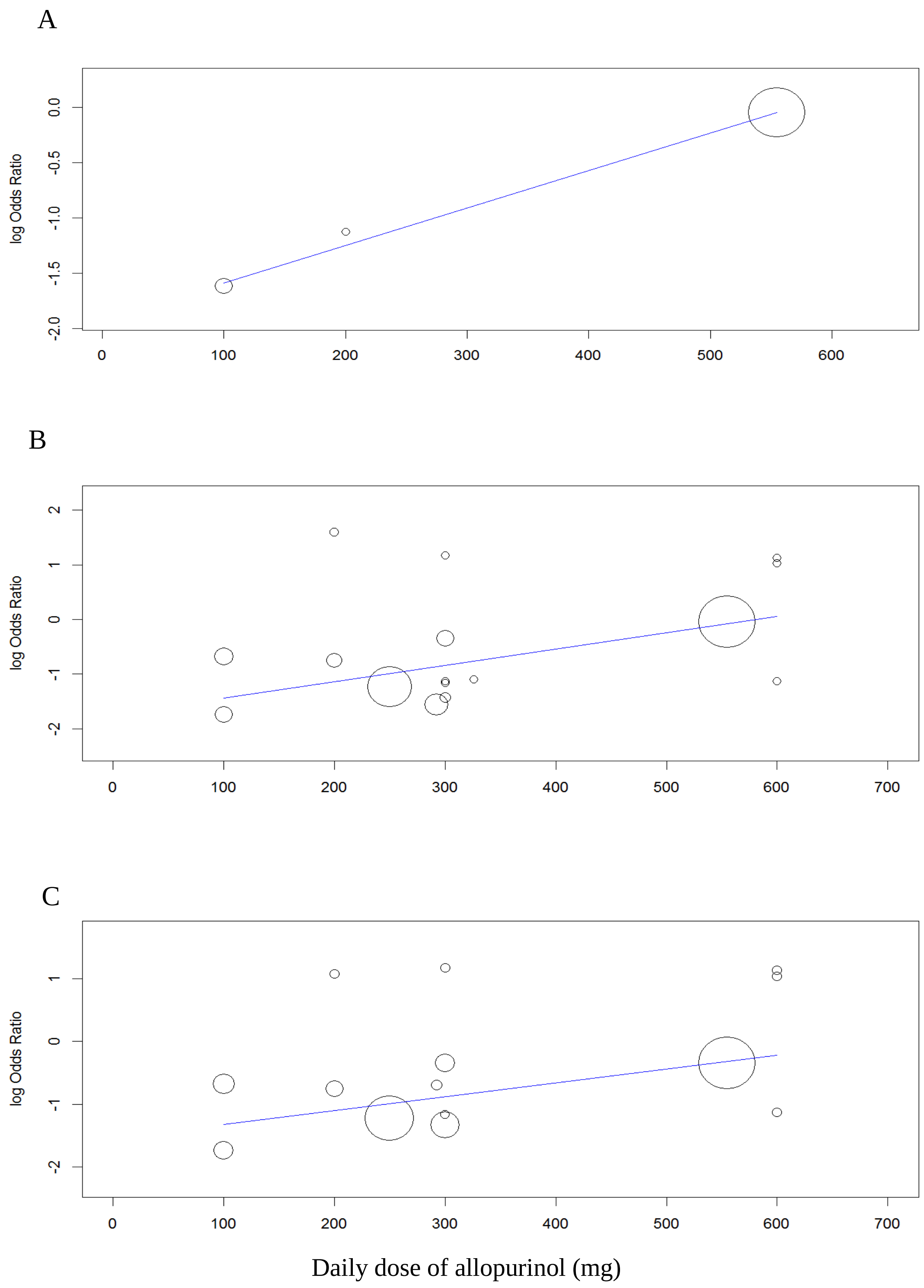

Figure S19: Random-effects meta-regression analyses of dose of allopurinol and log odds ratio of new/worsening heart failure (A), total cardiovascular events (B), and serious cardiovascular events (C) among studies at low or unknown risk of bias.

A

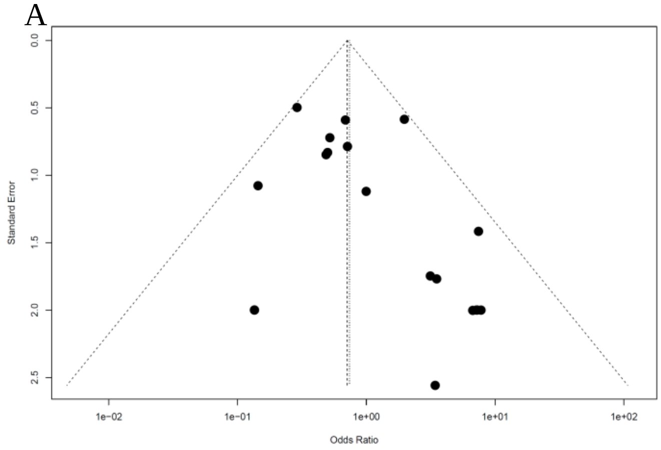

Standard Error
0.0
0.5
1.0
1.5
2.0
2.5
1e−02
1e−01
1e+00
1e+01
1e+02
Odds Ratio

B

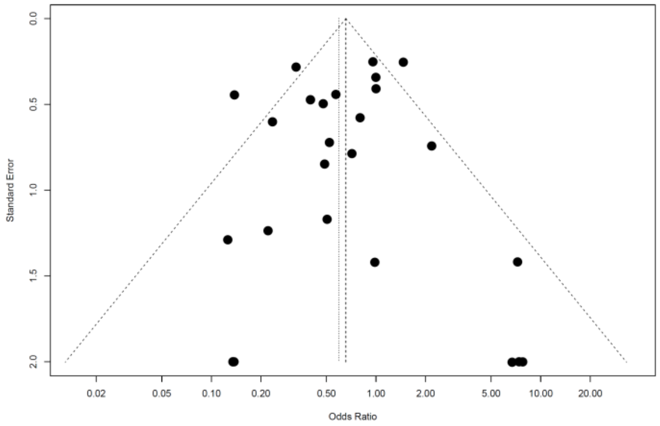

Standard Error
0.0
0.5
1.0
1.5
2.0
0.02
0.05
0.10
0.20
0.50
1.00
2.00
5.00
10.00
20.00
Odds Ratio

C

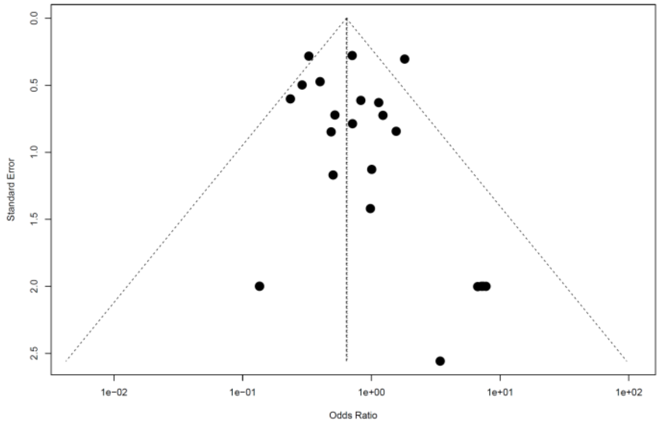

Standard Error
0.0
0.5
1.0
1.5
2.0
2.5
1e−02
1e−01
1e+00
1e+01
1e+02
Odds Ratio

D

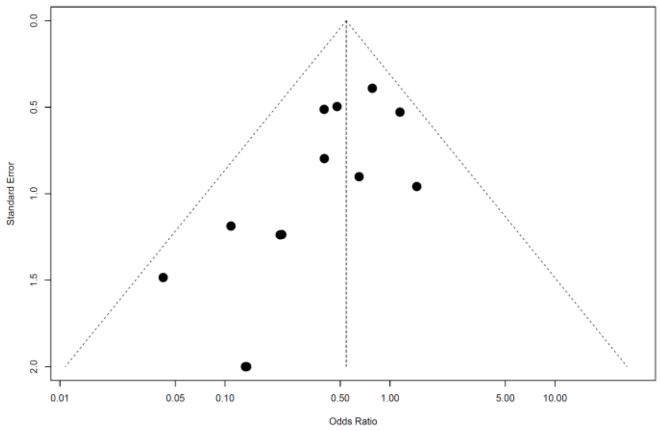

E

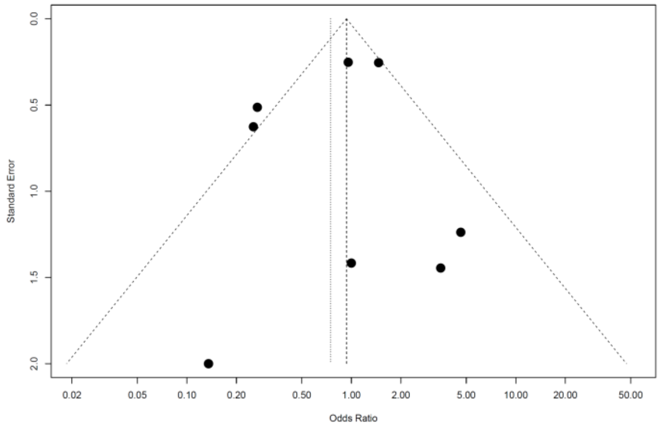

Figure S20: Funnel plot analyses of the outcomes major cardiovascular events (A; Egger's test, P=0.113), total cardiovascular events (B; P=0.473), serious cardiovascular events (C; P=0.452), new/worsening hypertension (D; P=0.018), and new/worsening heart failure (E; Egger's test not done).

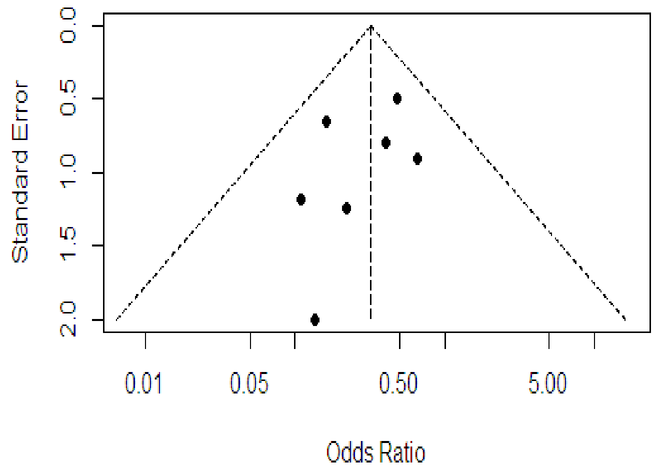

Figure S21: Funnel plot analysis of the outcome new/worsening hypertension in allopurinol/oxypurinol studies.

Studies	Estimate (95% C.I.)	Ev/Trt	Ev/Ctrl
Chen 2009	1.000 (0.000, Inf)	0/40	0/40
Dawson 2009	1.085 (0.000, Inf)	0/23	0/25
Deng 2010	1.060 (0.000, Inf)	0/33	0/35
Gibson 1982	1.267 (0.000, Inf)	0/26	0/33
Givertz 2015	0.957 (0.584, 1.568)	58/128	58/125
Goicoechea 2010	0.400 (0.158, 1.010)	7/57	15/56
Goldfarb 2013	1.000 (0.000, Inf)	0/33	0/33
Hare 2008	1.465 (0.890, 2.411)	44/203	32/202
Higgins 2014	0.714 (0.153, 3.337)	3/40	4/39
Hosoya 2016 Phase 2b	1.000 (0.000, Inf)	0/39	0/39
Jalal 2016	7.778 (0.154, 392.460)	1/39	0/41
Jalalzadeh 2012	0.965 (0.000, Inf)	0/28	0/27
Jarnerot 2000	7.241 (0.449, 116.640)	2/98	0/95
Joelsson 2001	0.958 (0.000, Inf)	0/94	0/90
Kanbay 2011	1.248 (0.000, Inf)	0/32	0/40
Lei 2009	0.966 (0.000, Inf)	0/29	0/28
Liu 2007	0.959 (0.000, Inf)	0/24	0/23
Liu 2015	0.478 (0.181, 1.264)	6/88	12/88
Mao 2015	0.327 (0.188, 0.569)	13/366	41/366
Modabber 2009	0.905 (0.000, Inf)	0/10	0/9
Momeni 2010	1.000 (0.000, Inf)	0/20	0/20
Poiley 2016	0.221 (0.020, 2.492)	1/55	2/28
Rekhraj 2013	7.389 (0.147, 372.385)	1/33	0/33
Robertson 2015	1.000 (0.000, Inf)	0/25	0/25
Sarris 2007	1.000 (0.000, Inf)	0/18	0/18
Schumacher 2008	0.189 (0.059, 0.610)	4/268	9/134
Shi 2012	0.907 (0.000, Inf)	0/21	0/19
Siu 2006	1.076 (0.000, Inf)	0/26	0/28
Szwejkowski 2013	0.135 (0.003, 6.820)	0/33	1/33
Taheraghdam 2014	0.487 (0.093, 2.567)	2/35	4/35
Takir 2015	0.826 (0.000, Inf)	0/40	0/33
Tan 2014	0.400 (0.158, 1.010)	7/57	15/56
Togha 2007	1.108 (0.000, Inf)	0/18	0/20
Wang 2012	1.034 (0.000, Inf)	0/29	0/30
Wang 2015	0.977 (0.000, Inf)	0/43	0/42
Yin 2015	0.928 (0.000, Inf)	0/41	0/38
Zhang 2012	0.138 (0.058, 0.330)	11/40	31/40
Ziaee 2006	0.932 (0.000, Inf)	0/29	0/27
**Overall**	**0.599 (0.473, 0.757)**	**160/2261**	**224/2093**

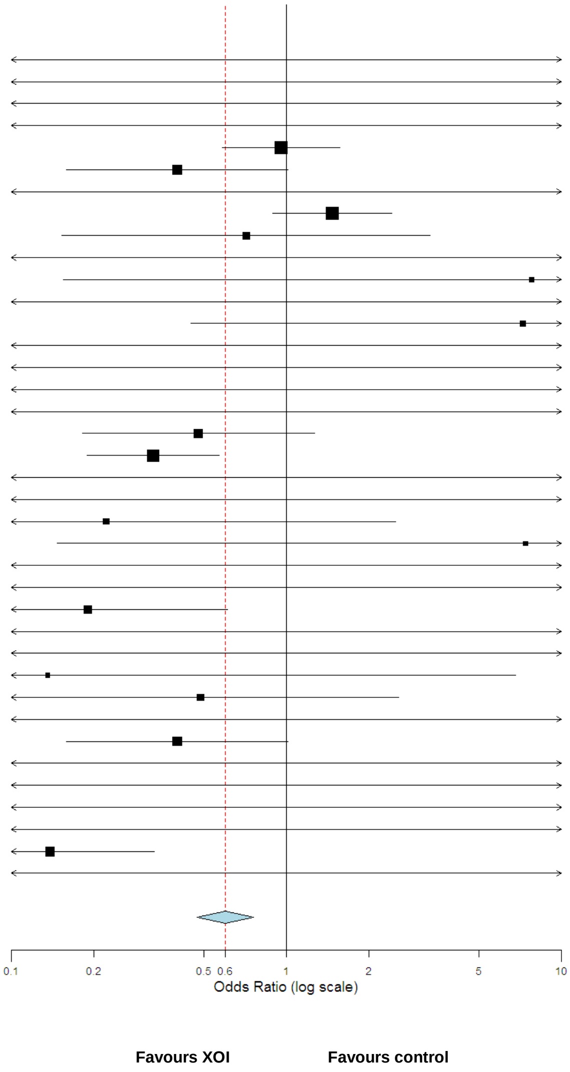

Figure S22: Forest plot comparing the risk of total cardiovascular events of allopurinol/oxypurinol versus control among studies lasting 90 days or longer.

Studies	Estimate (95% C.I.)	Ev/Trt	Ev/Ctrl
Deng 2010	1.060 (0.000, Inf)	0/33	0/35
Gibson 1982	1.267 (0.000, Inf)	0/26	0/33
Givertz 2015	0.688 (0.216, 2.190)	5/128	7/125
Goldfarb 2013	1.000 (0.000, Inf)	0/33	0/33
Higgins 2014	0.714 (0.153, 3.337)	3/40	4/39
Jarnerot 2000	7.166 (0.142, 361.329)	1/98	0/95
Joelsson 2001	0.958 (0.000, Inf)	0/94	0/90
Lei 2009	0.966 (0.000, Inf)	0/29	0/28
Liu 2007	0.959 (0.000, Inf)	0/24	0/23
Liu 2015	1.000 (0.000, Inf)	0/88	0/88
Mao 2015	0.135 (0.003, 6.820)	0/366	1/366
Modabber 2009	0.905 (0.000, Inf)	0/10	0/9
Rekhraj 2013	1.000 (0.000, Inf)	0/33	0/33
Robertson 2015	1.000 (0.000, Inf)	0/25	0/25
Sarris 2007	1.000 (0.000, Inf)	0/18	0/18
Schumacher 2008	0.473 (0.000, Inf)	0/268	0/134
Shen 2010	1.000 (0.000, Inf)	0/26	0/26
Shi 2012	0.907 (0.000, Inf)	0/21	0/19
Siu 2006	1.076 (0.000, Inf)	0/26	0/28
Szwejkowski 2013	1.000 (0.000, Inf)	0/33	0/33
Togha 2007	1.108 (0.000, Inf)	0/18	0/20
Zhang 2012	0.290 (0.109, 0.769)	6/40	16/40
Zhou 2009	0.922 (0.000, Inf)	0/51	0/47
**Overall**	**0.480 (0.250, 0.922)**	**15/1528**	**28/1387**

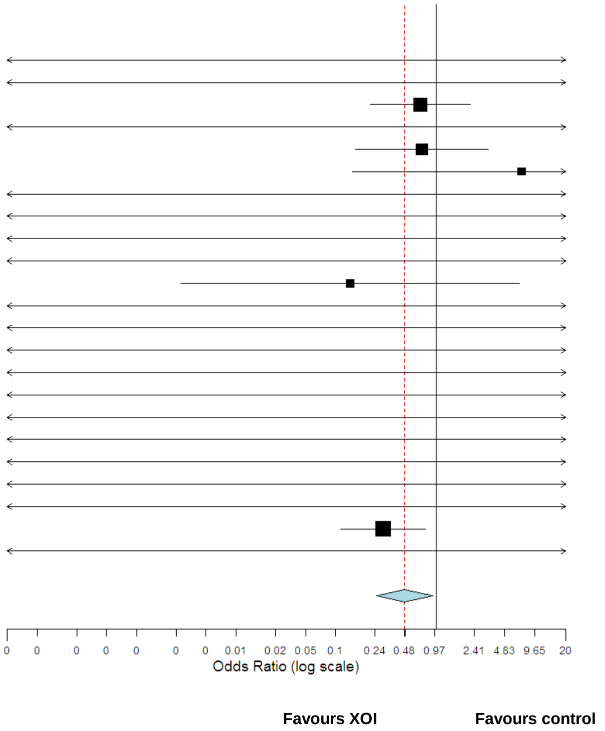

Figure S23: Forest plot comparing the risk of MACE of allopurinol/oxypurinol versus control among studies lasting 180 days or longer.
