# Supplementary material for: Xanthine oxidase inhibitors for prevention of cardiovascular events: a systematic review and meta-analysis of randomized controlled trials
Source: BMC Cardiovasc Disord. 2018 Feb 7;18:24. doi: 10.1186/s12872-018-0757-9 (PMC5804046; doi:10.1186/s12872-018-0757-9)
Supplement: Supplementary file 2 — Supplementary file containing description of studies included in the meta-analysis. (PDF 703 kb) [file 12872_2018_757_MOESM2_ESM.pdf]

ADDITIONAL FILE 2. Bredemeier et al. Xanthine Oxidase Inhibitors for Prevention of Cardiovascular Events: A Systematic Review and Meta-Analysis of Randomized Controlled Trials. BMC Cardiovascular Disorders.

Description of the studies included in the meta-analysis.

Study	Study Design	Publication format	Population	Intervention vs comparator	Age (mean)	Male (%)	Prevalence of gout (%)	Prevalence of risk factors or cardiovascular diseases (%)	Use of medications (%)	Duration of follow-up (days)	Industry Sponsoring
Akhondzadeh, 2005 (1)	Parallel	Full text	Schizophrenia	Allopurinol 300mg vs Placebo	34.4	71.7	NR	NR	NR	60	No
Akhondzadeh, 2006 (2)	Parallel	Full text	Manic episode	Allopurinol 300mg vs Placebo	26.2	51.2	NR	NR	NR	60	No
Becker, 2005 (3)	Parallel	Full text	Gout	Febuxostat 40-80-120mg vs Placebo	54	90.8	100	SAH 49, DM 13	SA 0	30	Yes (TAP Pharmaceutical Products)
Borgi, 2017 (4)	Parallel	Full text	Overweight/ obese, Hyperuricemic	Allopurinol 300-600mg vs Placebo	41	48.9	NR	Sm 8.1	NR	60	No (National Institutes of Health/National Heart, Lung, and Blood Institute)
Bowden, 2013(5)	Parallel	Full text	CKD	Allopurinol 300mg vs Placebo	60.8	58.3	NR	CKD 100, DM 54.2	NR	56	No
Brunstein, 2005 (6)	Cross - over	Full text	Schizophrenia	Allopurinol 600mg vs Placebo	38.6	60.9	0	HF 0, IC 0, CKD 0, MI 0, S 0, SAH 0, DM 0	ACEi/ARB 0, D 0, St 0, AS 0	42	No
Chen, 2009 (7)	Parallel	Full text	HF, Hyperuricemic	Allopurinol 300mg vs no treatment	60	60	0	HF 100, CKD 0, IC 42.5	NR	90	NR

ACEi – Angiotensin-converting enzyme inhibitors, ARB – Angiotensin II receptor blockers, CKD - Chronic kidney disease, CVD – Cardiovascular disease, D – Diuretics, DM – Diabetes mellitus, HF - Heart failure, IC - Ischemic cardiomyopathy, LVH – Left ventricular hypertrophy, MI - Myocardial infarction, NR – Not reported, S – Stroke, SA – Salicylic acid, SAH – Systemic arterial hypertension, Sm – Smoking, St – Statins

Chen, 2014 (8)	Parallel	Full text	Hemodialysis	Allopurinol 200-300mg vs no treatment	NR	51.6	NR	CKD 100	NR	270	NR
Cingolani, 2006 (9)	Parallel	Full text	HF	Oxypurinol 600mg vs Placebo	68.6	61.7	0	HF 100, IC 55	ACEi/ARB 80, St 25, SA 63.3	30	Yes (Cardiome Pharma Corp).
Dawson, 2009 (10)	Parallel	Full text	Subcortical stroke	Allopurinol 300mg vs Placebo	58.3	NR	0	MI 0, S 100, SAH 59.1, DM 10.2, Sm 44.9	ACEi/ARB 55.1, D 34.7, St 93.9, SA 95.9	90	No (West Endowments Research Fund)
Deng, 2010 (11)	Parallel	Full text	CKD, hyperuricemia	Allopurinol 100-300mg vs No treatment	59.3	52.4	NR	CKD 100, DM 34.4	NR	365	NR
Dickerson, 2009 (12)	Parallel	Full text	Schizophrenia	Allopurinol 600mg vs Placebo	43.1	66	0	DM 0, HF 0, IC 0, CKD 0, MI 0, S 0	NR	60	No (Stanley Medical Research Institute)
Dogan, 2011 (13)	Parallel	Full text	DM	Allopurinol 900mg vs Placebo	50.3	51	0	HF 0, IC 0, CKF 0, MI 0, S 0, SAH 0, DM 100, Sm 0	ACEi/ARB 0, St 74, SA 85	84	No
Eddeland, 1983 (14)	Parallel	Full text	Indwelling Urethral Catheters	Allopurinol 300mg vs Placebo	76.2	23.3	NR	NR	NR	210 (mean)	No
Ettinger, 1986 (15)	Parallel	Full text	Recurrent calcium oxalate stone disease	Allopurinol 300mg vs Placebo	47.6	NR	NR	CKD 0	NR	168	Partial (Burroughs Wellcome Company)
Fan, 2012 (16)	Parallel	Full text	Manic episode	Allopurinol 600mg vs Placebo	42.8	51.8	0	HF 0, IC 0, CKD 0, MI 0, S 0	NR	42	NR

ACEi – Angiotensin-converting enzyme inhibitors, ARB – Angiotensin II receptor blockers, CKD - Chronic kidney disease, CVD – Cardiovascular disease, D – Diuretics, DM – Diabetes mellitus, HF - Heart failure, IC - Ischemic cardiomyopathy, LVH – Left ventricular hypertrophy, MI - Myocardial infarction, NR – Not reported, S – Stroke, SA – Salicylic acid, SAH – Systemic arterial hypertension, Sm – Smoking, St – Statins

Feuerman, 1973 (17)	Cross-over	Full text	Psoriasis	Allopurinol 600mg vs Placebo	48.4	61.5	0	HF 0, IC 0, CKD 0, MI 0, S 0, SAH 0, DM 15.4	NR	56	No
George, 2006 (18)	Cross-over	Full text	HF	Allopurinol 300-600mg vs Placebo	69.7	83.3	NR	HF 100, CKD 0, MI 70, CI 96.6 , SAH 60, Sm 10	ACEi/ARB 89.9, **D 0**, SA 80, St 90	28	No (British Heart Foundation)
Gibson, 1982 (19)	Parallel	Full text	Gout	Allopurinol 200mg vs Placebo	49	98.3	100	CKD 0, SAH 22	NR	730	No (Arthritis and Rheumatism Council)
Givertz, 2015 (20)	Parallel	Full text	HF	Allopurinol 600mg vs Placebo	63	82.2	22.5	HF 100, IC 53.3, MI 0, SAH 78.2, DM 54.5	ACEi/ARB 84.6	180	No (National Institutes of Health)
*Goicoechea, 2010 (21)	Parallel	Full text	CKD	Allopurinol 100mg vs No treatment	71.7	NR	NR	IC 23.5, CKD 100, DM 37.2	ACEi/ARB 77.9, D 58.4, St 45.1	702 (mean)	No
Goldfarb, 2013 (22)	Parallel	Full text	Hyperuricosuric	Allopurinol 300mg vs Febuxostat 80mg vs Placebo	47.4	85.9	0	CKD 61.2, MI 30.3, S 0, SAH 27.2, DM 12.2, Sm 21.2	ACEi 18.2, St 28.3, D 5.1	180	Yes (Takeda Global Research & Development Center)
Greig, 2011 (23)	Parallel	Full text	HF	Allopurinol 300mg vs Placebo	59.5	82.4	NR	HF 100, IC 21.6, SAH 59.5, DM 14.9, Sm 13.5	ACEi/ARB 91.9, D 77, St 100	28	No (FONDECYT, FONDAP)
Guo, 2015 (24)	Parallel	Full text	Hyperuricemic, Acute ischemic stroke	Allopurinol 300mg vs no treatment	67.4	63.7	0	HF 0, CKD 0, DM 0	NR	84	NR

ACEi – Angiotensin-converting enzyme inhibitors, ARB – Angiotensin II receptor blockers, CKD - Chronic kidney disease, CVD – Cardiovascular disease, D – Diuretics, DM – Diabetes mellitus, HF - Heart failure, IC - Ischemic cardiomyopathy, LVH – Left ventricular hypertrophy, MI - Myocardial infarction, NR – Not reported, S – Stroke, SA – Salicylic acid, SAH – Systemic arterial hypertension, Sm – Smoking, St – Statins

Guo, 2016 (25)	Parallel	Full text	Acute Ischemic Stroke, hyperuricemia	Allopurinol vs no treatment	32	61.2	NR	DM 0	NR	90	NR
Hare, 2008 (26)	Parallel	Full text	HF	Oxypurinol 600mg vs Placebo	64.5	72.8	NR	HF 100, IC 61	D 95	168	Yes (Cardiome Pharma Corp.)
Higgins, 2014 (27)	Parallel	Full text	Stroke and Transient ischaemic attack	Allopurinol 300mg vs Placebo	67.9	57.5	0	SAH 42.5, DM 13.7, Sm 18.7, IC 10	ACEi/ARB 48.75, D 31.25, SA 92.5, St 92.5	360	No (The stroke Association, UK and Scottish Stroke Research Network)
Hill, 2015 (28)	Parallel	Full text	Gout	Allopurinol 200mg vs Placebo	55.5	94.3	100	NR	NR	28	No
Hosoya, 2014 (29)	Parallel	Full text	Hyperuricemia CKD	Topiroxostat 160mg vs Placebo	63.5	89.3	NR	CKD 100, DM 35,2	ACEi/ARB 56,5	154	Yes (Sanwa Kagaku Kenkyusho Co.)
Hosoya, 2016 fase 2a (30)	Parallel	Full text	Gout, hyperuricemia	Topiroxostat 40-60-80-120mg vs Placebo	45.5	100	66.6	DM 0, CKD 0	NR	60	Yes (Fuji Yakuhin Co.)
Hosoya, 2016 fase 2b (31)	Parallel	Full text	Hyperuricemia	Allopurinol 200mg vsTopiroxostat 120-160mg vs Placebo	51.4	98	NR	CKD 0	NR	120	Yes (Fuji Yakuhin Co.)

ACEi – Angiotensin-converting enzyme inhibitors, ARB – Angiotensin II receptor blockers, CKD - Chronic kidney disease, CVD – Cardiovascular disease, D – Diuretics, DM – Diabetes mellitus, HF - Heart failure, IC - Ischemic cardiomyopathy, LVH – Left ventricular hypertrophy, MI - Myocardial infarction, NR – Not reported, S – Stroke, SA – Salicylic acid, SAH – Systemic arterial hypertension, Sm – Smoking, St – Statins

Jahangard, 2014 (32)	Parallel	Full text	Manic episode	Allopurinol 600mg vs Placebo	34.4	61.4	NR	HF 0, IC 0, MI 0, S 0 , DM 0, CKD 0, Sm 0, SAH 0	NR	28	No
Jalal, 2016 (33)	Parallel	Full text	CKD, hyperuricemia	Allopurinol 300mg vs Placebo	57.4	79.9	0	HF 0, CKD 100, DM 61, Sm 17.5 CVD 45	NR	90	No (National Institutes of Health)
Jalalzadeh, 2012 (34)	Cross-over	Full text	Hemodialysis, hyperuricemia	Allopurinol 100mg vs standard treatment	55.9	62.3	NR	CDK 100, SAH 100	ACEi 54.7	90	No (Iran's Zanjan University of Medical Sciences)
Jarnerot, 2000 (35)	Parallel	Full text	Ulcerative colitis	Allopurinol 200mg vs Placebo	44	52.8	NR	Sm 11.4	NR	365	No
Jitapunkul, 1991 (36)	Cross-over	Full text	DM	Allopurinol 300mg vs Placebo	51.3	NR	NR	CKD 100, HF 0, IC 0, MI 0, S 0, DM 100	D 0, SA 0	84	No
Joelsson, 2001 (37)	Parallel	Full text	Ulcerative colitis	Allopurinol 200mg vs Placebo	38	59.8	NR	NR	NR	730	No (Swedish Medical Research Council)
Kamatami late phase 2, 2011 (38)	Parallel	Full text	Gout, Asymptomatic hyperuricemia	Febuxostat 20-40-60-80 vs Placebo	53.6	97	59.3	CKD 0, SAH 48.7, DM 10.5	NR	120	Yes (Teijin Pharma Ltd.)
Kamatani Phase 3, 2011 (39)	Parallel	Full text	Hyperuricemia	Febuxostat 20mg vs Febuxostat 40mg vs Placebo	47.5	100	100	SAH 35.2 CKD 7.8 DM 10.7	NR	60	Yes (Teijin Pharma Ltd.)

ACEi – Angiotensin-converting enzyme inhibitors, ARB – Angiotensin II receptor blockers, CKD - Chronic kidney disease, CVD – Cardiovascular disease, D – Diuretics, DM – Diabetes mellitus, HF - Heart failure, IC - Ischemic cardiomyopathy, LVH – Left ventricular hypertrophy, MI - Myocardial infarction, NR – Not reported, S – Stroke, SA – Salicylic acid, SAH – Systemic arterial hypertension, Sm – Smoking, St – Statins

Kanbay, 2011 (40)	Parallel	Full text	Asymptomatic hyperuricemia	Allopurinol 300mg vs No treatment	52.1	50.2	0	HF 0, IC 0, CKD 0, MI 0, S 0, SAH 0, DM 0, Sm 0	ACEi/ARB 0, St 0	120	No
Kao, 2011 (41)	Parallel	Full text	LVH, CKD	Allopurinol 300mg vs Placebo	72.1	52.8	0	HF 0, CKD 100, Sm 13.2	ACEi/ARB 73.6, D 45.3, St 77.3	270	No (British Heart Foundation)
Khan, 2008 (42)	Parallel	Full text	S, Hyperuricemia	Allopurinol 300mg vs Placebo	68.5	78.6	NR	IC 25, S 100, DM 3.6, Sm 3.6	ACEi/ARB 64.3, D 46.4, St 89.3	56	No (Chest, Heart and Stroke Scotland, Heart Research UK)
Lei, 2009 (43)	Parallel	Full text	CKD, hyperuricemia	Allopurinol 100-200mg vs no Placebo	49	68.4	NR	CKD 100	NR	365	NR
Liu, 2007 (44)	Parallel	Full text	CKD, hyperuricemia	Allopurinol 100-300mg vs No treatment	46.0	61.6	NR	CKD 100	NR	365	NR
Liu, 2015 (45)	Parallel	Full text	Type2 diabetes	Allopurinol 100mg vs no treatment	50.4	46.02	0	CKD 0, SAH 0, DM 100, Sm 34	St 33.7, AS 48.6	1095	NR
Machado-Vieira, 2008(46)	Parallel	Full text	Bipolar disorder	Allopurinol 600mg vs Placebo	28.1	41.7	NR	NR	NR	30	No (Stanley Medical Research Institute)
Madero, 2015 (47)	Parallel	Full text	Overweight and prehypertensive subjects	Allopurinol 300mg vs Placebo	46.1	61.1	0	HF 0, IC 0, CKD 0, MI 0, S 0, SAH 0, DM 0	NR	28	NR
Mao, 2015 (48)	Parallel	Full text	Coronary Heart Disease	Allopurinol 300mg vs Placebo	46	57.1	NR	IC 54	NR	730	NR

ACEi – Angiotensin-converting enzyme inhibitors, ARB – Angiotensin II receptor blockers, CKD - Chronic kidney disease, CVD – Cardiovascular disease, D – Diuretics, DM – Diabetes mellitus, HF - Heart failure, IC - Ischemic cardiomyopathy, LVH – Left ventricular hypertrophy, MI - Myocardial infarction, NR – Not reported, S – Stroke, SA – Salicylic acid, SAH – Systemic arterial hypertension, Sm – Smoking, St – Statins

Modabber, 2009 (49)	Cross-over	Full text	Schizophrenia	Allopurinol 600mg vs Placebo	36.2	NR	0	HF 0, IC 0, CKD 0, MI 0, S 0, SAH 0, DM 0	NR	180	No (Kurdistan University of Medical Sciences Vice Chancellor for research)
Momeni, 2010 (50)	Parallel	Full text	DM, CKD	Allopurinol 100mg vs Placebo	57.7	45	NR	DM 100, CKD 100	ACEi/ARB 100	120	No
Muir, 2008 (51)	Parallel	Full text	Acute ischemic stroke	Allopurinol 100-300mg vs Placebo	69.9	42.3	NR	S 100, DM 12	NR	42	No (Glasgow West Endowments Fund.)
NCT01078389, 2014 (52)	Parallel	ClinicalTrials	Gout	Topiroxostat 40/80mg vs Placebo	50.8	91.7	100	Sm 18.1, CKD 71.6	NR	730	Yes (Takeda)
NCT01350388 / Beddhu, 2016 (53)	Parallel	ClinicalTrials /Full text	CKD, DM	Febuxostat 80mg vs Placebo	68	65	NR	CKD 100, DM 100	NR	180	No (University of Utah)
NCT01496469, 2015 (54)	Parallel	ClinicalTrials	SAH, hyperuricemia	Febuxostat 80mg vs Placebo	53.6	80.9	0	Sm14.8, SAH 100, HF0, CI0,S 0, MI0, CKD62.8	ACEi/ARB 44.6	45	Yes (Takeda)
NCT02128490 / Gunawardhana, 2016 (55)	Parallel	ClinicalTrials	Gout, CKD	Febuxostat 40I, 40X, 80I, 80Xmg vs Placebo	63.1	70.8	100	Sm 10	NR	90	Yes (Takeda)
NCT02139046 / Saag, 2016 (56)	Parallel	ClinicalTrials	Gout	Febuxostat 40I-40X-80I-80Xmg vs Placebo	55.1	88.4	100	Sm 16	NR	90	Yes (Takeda)

ACEi – Angiotensin-converting enzyme inhibitors, ARB – Angiotensin II receptor blockers, CKD - Chronic kidney disease, CVD – Cardiovascular disease, D – Diuretics, DM – Diabetes mellitus, HF - Heart failure, IC - Ischemic cardiomyopathy, LVH – Left ventricular hypertrophy, MI - Myocardial infarction, NR – Not reported, S – Stroke, SA – Salicylic acid, SAH – Systemic arterial hypertension, Sm – Smoking, St – Statins

Noman, 2010 (57)	Cross-over	Full test	Stable chronic angina pectoris	Allopurinol 600mg vs placebo	64.6	83	0	SAH 45, DM 12, IC 100 , HF 15 , Sm 8	ACEi 56.6, St 97, SA 100	42	No (British Heart Foundation)
Parmley 1992 (58)	Parallel	Full text	Ischemic cardiomyopathy	Allopurino 300mg vs Placebo	54.5	73.5	NR	IC 100, CKD 0, SAH 52.1, DM 30.5, Sm 79.3	NR	90	Yes (Burroughs Wellcome Company)
Poiley, 2016 (59)	Parallel	Full text	Gout	Allopurinol 300mg vs Placebo	52.1	95.1	100	HF 0, CKD 0, MI 0, S 0	NR	90	Yes (CymaBay Therapeutics)
Puntoni, 2013 (60)	Parallel	Full text	Colorectal Adenoma	Allopurinol 200mg vs Allopurinol 100mg vs Placebo	61.3	53.4	0	Sm 11	NR	28	Yes (Italian League Against Cancer, the Ligurian Public Health Regional Agency, Cassa di Risparmio di Genova e Imperia—CA.RI.GE. Foundation, and Edoardo Garrone Foundation)
Rassi, 2007 (61)	Parallel	Full text	Chronic phase of Chagas disease	Allopurinol 900mg vs Placebo	48.5	45.7	NR	CKD 0, HF 0, IC 0, LVH 0, MI 0,	D 0	60	No (UNDP/WORLD BANK/WHO SPECIAL PROGRAMME)

ACEi – Angiotensin-converting enzyme inhibitors, ARB – Angiotensin II receptor blockers, CKD - Chronic kidney disease, CVD – Cardiovascular disease, D – Diuretics, DM – Diabetes mellitus, HF - Heart failure, IC - Ischemic cardiomyopathy, LVH – Left ventricular hypertrophy, MI - Myocardial infarction, NR – Not reported, S – Stroke, SA – Salicylic acid, SAH – Systemic arterial hypertension, Sm – Smoking, St – Statins

Rekhraj, 2013 (62)	Parallel	Full text	Ischemic heart disease	Allopurinol 600mg vs Placebo	64.5	90	0	Sm 16.6. CKD 0, SAH 65, DM 11.6, MI 50, HF 0, IC 100, S 10	ACEi 58.3, ARB 16.6, St 93, SA 91.6	270	No (Medical Research Council - (UK)
Rentoukas, 2010 (63)	Parallel	Full text	STEMI undergoing primary PCI	Allopurinol 400-100mg vs Placebo	63.9	72.5	NR	IC 100, MI 17.45, SAH 25, DM 22.5, Sm 27.5	D 22.5	30	NR
Robertson, 2015 (64)	Parallel	Full text	Peripheral Arterial Disease	Allopurinol 600mg vs Placebo	68.4	78	NR	HF 0, IC 0, CKD 0, Sm 26	ACEi/ARB 70, St 94, SA 94	180	No (British Heart Foundation)
Rosenfeld, 1974 (65)	Parallel	Full text	Hyperuricemic	No fixed dose	50.3	NR	NR	CKD 68.3, SAH 78	CKD 68.3, SAH 70	900	NR
Saag, 2016 (66)	Parallel	Full text	CKD,gout	Febuxostat 60-40-80mg vs Placebo	65.7	80.2	100	CKD 100, SAH 95.8, DM 44.8	ACEi/ARB 65.6, SA 54.2	365	Yes (Takeda)
Sarris, 2007 (67)	Parallel	Abstract	CKD	Allopurinol 150mg vs no treatment	49.8	47.2	0	HF 0, CKD 100	NR	365	NR
Schumacher, 2008 (68)	Parallel	Full text	Hyperuricemia and gout	Allopurinol 300-100mg vsFebuxostat 80-120-240mg vs Placebo	51.7	93.7	100	CVD 13,4, SAH 46.7	SA 17	210	Yes (Takeda Global Research & Development Center, Inc.)

ACEi – Angiotensin-converting enzyme inhibitors, ARB – Angiotensin II receptor blockers, CKD - Chronic kidney disease, CVD – Cardiovascular disease, D – Diuretics, DM – Diabetes mellitus, HF - Heart failure, IC - Ischemic cardiomyopathy, LVH – Left ventricular hypertrophy, MI - Myocardial infarction, NR – Not reported, S – Stroke, SA – Salicylic acid, SAH – Systemic arterial hypertension, Sm – Smoking, St – Statins

Segal, 2015 (69)	Parallel	Full text	SAH<br>CKD	Allopurinol 300-600mg vs Placebo	50.9	28	0	SAH 100	ACEi/ARB 36.5, D 48.2	67	No (NIH/NCATS and Divison of Nephology Gatorade Research Funds)
Separham, 2016 (70)	Parallel	Full text	MI	Allopurinol 100mg vs Placebo	60.6	77.9	NR	MI 100, DM 25.7, SAH 42.1, Sm 42.1	NR	30	No
Shen, 2010 (71)	Parallel	Full text	CKD, hyperuricemia	Allopurinol 100-200mg vs No treatment	47.3	67.3	0	CKD 100	NR	365	NR
Shi, 2012 (72)	Parallel	Full text	IgA nephropathy	Allopurinol 100-300mg vs No treatment	39.9	55	NR	SAH 45	ACEi/ARB 0	180	No (Scientific and Technologic Commitee of Guangdong Province)
Sircar, 2015 (73)	Parallel	Full text	CKD, hyperuricemia	Febuxostat 40mg vs Placebo	57.3	70.7	0	CKD 100, SAH 97.9, DM 37.2, IC 37.7	ACEi/ARB 65.6, D 18.5	180	No
Siu, 2006 (74)	Parallel	Full text	CDK, Hyperuricemic	Allopurinol 200-100mg vs Placebo	48.2	43.1	0	CKD 94.4, SAH 78.3, DM 25.5	ACEi 53.7 ARB 24.0 St 33.7	360	No
Szwejkowski, 2013 (75)	Parallel	Full text	Type 2 Diabetes LVH	Allopurinol 600mg vs Placebo	64.6	61	0	CKD 0, HF 100, IC 10.2, S 10.67, SAH 89.8, DM 100, Sm 13.6	ACEi 49.15, ARB 27.12, D 28.81; St 84.75, SA 47.46	270	No (Diabetes UK)

ACEi – Angiotensin-converting enzyme inhibitors, ARB – Angiotensin II receptor blockers, CKD - Chronic kidney disease, CVD – Cardiovascular disease, D – Diuretics, DM – Diabetes mellitus, HF - Heart failure, IC - Ischemic cardiomyopathy, LVH – Left ventricular hypertrophy, MI - Myocardial infarction, NR – Not reported, S – Stroke, SA – Salicylic acid, SAH – Systemic arterial hypertension, Sm – Smoking, St – Statins

Taheraghdam, 2014 (76)	Parallel	Full text	Acute ischemic stroke	Allopurinol 200mg vs Placebo	69	35.7	0	HF 0, IC 28.6, CKD 0, S 100, MI 12.8, SAH 35.7, DM 62.8, Sm 14.3	ACEi/ARB 35.7, D 0, St 100	90	No
Takir, 2015 (77)	Parallel	Full text	Hyperuricemia	Allopurinol 300mg vs Placebo	48.4	33.8	0	DM 0, CKD 0, SAH 52.9	ACEi/ARB 53.7	90	No (Istambul Medeniyet University)
Tan, 2014 (78)	Parallel	Full text	CKD	Allopurinol 66.6 - 200mg vs No treatment	NR	NR	NR	CKD 100	NR	730	NR
Tanaka, 2015 (79)	Parallel	Full text	CDK, Hyperuricemic	Febuxostat 40mg vs Placebo	68.2	87.5	0	CKD 100, DM 6.7, SAH 42.5, Sm 20, CVD 11.1	NR	90	NR
Tani, 2015 (80)	Parallel	Full text	Hyperuricemic, Hypertension	Febuxostat 10mg vs Placebo	68	88	NR	CKD 43, IC 32, SAH 100, DM 32, Sm 8.3	ACEi/ARB 65, D 25, St 53	180	No
Togha, 2007 (81)	Parallel	Full text	Epilepsy	Allopurinol 300mg vs Placebo	24.9	55.3	0	HF 0, CKD 0, MI 0, CI 0 , S 0, SAH 0, DM 0	ACEi/ARB 0, D 0, SA 0, St 0	180	No (Iran Epilepsy Society)
Tsuruta, 2015 (82)	Parallel	Full text	Hemodialysis, hyperuricemia	Febuxostat 10mg vs Placebo	68.2	64.1	0	SAH 79.2, DM 41.4, CKD 100, IC 26.3	NR	30	No (Global Forum on Home Hemodialysis)
Usharani, 2016 (83)	Parallel	Full text	Hyperuricemia	Febuxostat 40mg vs Placebo	53	68	NR	CKD 0	D 0, SA 0	180	No

ACEi – Angiotensin-converting enzyme inhibitors, ARB – Angiotensin II receptor blockers, CKD - Chronic kidney disease, CVD – Cardiovascular disease, D – Diuretics, DM – Diabetes mellitus, HF - Heart failure, IC - Ischemic cardiomyopathy, LVH – Left ventricular hypertrophy, MI - Myocardial infarction, NR – Not reported, S – Stroke, SA – Salicylic acid, SAH – Systemic arterial hypertension, Sm – Smoking, St – Statins

Wang, 2012 (84)	Parallel	Full text	CKD, hyperuricemia	Allopurinol 300mg vs No treatment	46.2	72.8	NR	IC 0, Sm 22, SAH 27,1, DM 27,1, CKD 0,	D 0, SA 0	90	Impossible to extract
Wang, 2015 (85)	Parallel	Full text	Atherosclerosis hyperuricemia	Allopurinol 300mg vs no treatment	47.8	63.5	NR	CKD 0	NR	90	NR
Weiser, 2012 (86)	Parallel	Full text	Schizophrenia or schizoaffective disorder	Allopurinol 600mg vs Placebo	42.6	51.5	NR	NR	NR	60	No (Stanley Medical Research Institute)
Weiser, 2014 (87)	Parallel	Full text	Bipolar disorder	Allopurinol 300mg vs Placebo	46.7	32.8	NR	NR	NR	42	No (Stanley Medical Research Institute)
Yin, 2015 (88)	Parallel	Full text	Angina pectoris, hyperuricemia	Allopurinol 400mg vs no treatment	66.9	55.6	0	DM 25.3, SHA 32.9, Sm 46.8	SA 100	90	NR
Zhang, 2012 (89)	Parallel	Full text	Acute coronary syndromes, hyperuricemia, hyperlipidemia	Allopurinol 300mg vs no treatment	NR	75	0	IC 100, CKD 0, HF 0	NR	365	NR
Zhou, 2009 (90)	Parallel	Full text	CKD, hyperuricemia	Allopurinol 100-200mg vs Placebo	58.9	43	0	CKD 100, DM 32.5, SAH 52.3	ACEi/ARB 52.3	180	NR
Ziaee, 2006 (91)	Parallel	Full text	Chronic nonbacterial prostatitis	Allopurinol 100mg vs Placebo	33.3	100	NR	NR	NR	90	No

ACEi – Angiotensin-converting enzyme inhibitors, ARB – Angiotensin II receptor blockers, CKD - Chronic kidney disease, CVD – Cardiovascular disease, D – Diuretics, DM – Diabetes mellitus, HF - Heart failure, IC - Ischemic cardiomyopathy, LVH – Left ventricular hypertrophy, MI - Myocardial infarction, NR – Not reported, S – Stroke, SA – Salicylic acid, SAH – Systemic arterial hypertension, Sm – Smoking, St – Statins

Study	Primary outcome
Akhondzadeh, 2005 (1)	Mean decrease in Positive and Negative Syndrome Scale score (PANSS) over 8 weeks.
Akhondzadeh, 2006 (2)	Mean decrease in the Young Mania Rating Scale score from baseline over 8 weeks.
Becker, 2005 (3)	Proportion of subjects in each treatment group with sUA levels 6.0 mg/dl on day 28.
Borgi, 2017 (4)	Kidney-specific and systemic RAS (renin-angiotensin system) activity at 8 weeks.
Bowden, 2013(5)	Colesterol LDL, HDL, Triglycerides, and Apo B levels at 8 weeks.
Brunstein, 2005 (6)	Total, positive, negative and general PANNS score over 6 weeks.
Chen, 2009 (7)	Changes of serum uric acid level and NYHA class of heart function at 3 months.
Chen, 2014 (8)	Changes of serum uric acid level, blood pressure and C-reactive protein at 9 months.
Cingolani, 2006 (9)	Change in LVEF, uric acid, and 6-minute walk distance at 4 weeks.
Dawson, 2009 (10)	Cerebrovascular reactivity difference between baseline and 3 months.
Deng, 2010 (11)	Doubling of serum creatinine, advance to end-stage renal disease, and mortality at 12 months.
Dickerson, 2009 (12)	PANSS score over 8 weeks.
Dogan, 2011 (13)	Brachial artery flow-mediated dilatation (FMD) and nitrate-

	induced dilatation (NID) at 12 weeks.
Eddeland, 1983 (14)	The frequency of need for indwelling urethral catheter attention including catheter change over time.
Ettinger, 1986 (15)	Incidence of recurrent calcium oxalate calculi of the kidney over 24 weeks.
Fan, 2012 (16)	YMRS (Young Mania Rating Scale) at week 6.
Feuerman, 1973 (17)	Extent and severity of psoriatic lesions at 56 days.
George, 2006 (18)	Endothelial function was assessed by standard forearm venous occlusion plethysmography at 28 days.
Gibson, 1982 (19)	Decline in renal function over 2 years.
Givertz, 2015 (20)	Composite end point based on survival, worsening HF, and patient global assessment at 24 weeks.
Goicoechea, 2010 (21)	Incidence of hospitalizations, cardiovascular events, end-stage renal disease requiring dialysis therapy, and mortality at 24 months.
Goldfarb, 2013 (22)	Percent change frombaseline to month 6 in 24-hour urinary uric acid.
Greig, 2011 (23)	Malondialdehyde (MDA), extracellular superoxide<br><br>dismutase (ecSOD) activity, flow-dependent endothelial-mediated vasodilation (FDD), and functional capacity by 6-minute walk test (6MWT) after 4 weeks.
Guo, 2015 (24)	The levels of serum uric acid and neurological function at week 12.

Guo, 2016 (25)	The scores of Barthel index (BI) and modified Rankin Scale (mRS) were evaluated at 30 days and 90 days.
Hare, 2008 (26)	A composite end point comprising heart failure morbidity, mortality, and quality of life at week 24.
Higgins, 2014 (27)	Change in Central blood pressure (CBP), arterial stifness, and carotid intima-media thickness (CIMT) at 12 months.
Hill, 2015 (28)	Protocol defined days to resolution of acute gout.
Hosoya, 2014 (29)	Percent change of the serum urate level and eGFR from the baseline to the final visit at week 22.
Hosoya, 2016 fase 2a (30)	Percent change in serum urate level from baseline to the final visit at week 8.
Hosoya, 2016 fase 2b (31)	Serum urate reduction rate from baseline to the final visit at week 16.
Jahangard, 2014 (32)	Change in YMRS (Young Mania Rating Scale) over 4 weeks.
Jalal, 2016 (33)	Change in brachial artery flow-mediated dilation (BA-FMD) from the week 0 to week 12 visits.
Jalalzadeh, 2012 (34)	Lowering of systolic and diastolic blood pressure at 12 weeks.
Jarnerot, 2000 (35)	Maintenance of remission of ulcerative colitis over 12 months.
Jitapunkul, 1991 (36)	Levels of hemoglobin A1c, plasma glucose in 75-gr oral tolerance test, and insulin levels at week 12.
Joelsson, 2001 (37)	Incidence of pouchitis over 24 months.
Kamatami late phase 2, 2011 (38)	Percentage of patients achieving serum uric<br><br>acid levels 6.0 mg/dL or less at 16 weeks.

Kamatani Phase 3, 2011 (39)	Percentage of patients achieving serum uric acid levels 6.0 mg/dL or less at 8 weeks.
Kanbay, 2011 (40)	Flow-mediated dilation (FMD) and estimated glomerular filtration rate (eGFR) change from baseline to 16 weeks.
Kao, 2011 (41)	Left ventricular mass index (LVMI), endothelial function and central arterial stiffness change from baseline to 9 months.
Khan, 2008 (42)	Change in arterial wave reflection, determined from the augmentation index (AIx), from baseline to week 8.
Lei, 2009 (43)	Creatinine level and incidence of renal function deterioration at 12 months.
Liu, 2007 (44)	Increase if serum creatinine at 12 months.
Liu, 2015 (45)	Change in carotid IMT (intima-media thickness) after 3 years of treatment.
Machado-Vieira, 2008 (46)	Change in Young Mania Rating Scale (YMRS) over 4 weeks.
Madero, 2015 (47)	Clinic blood pressure reduction at 8 weeks.
Mao, 2015 (48)	BaPWV (brachial—ankle pulse wave velocity) and ABI (ankle brachial index) at 12 and 24 months.
Modabber, 2009 (49)	Changes in the mean Positive and Negative Syndrome Scale (PANSS) score at 6 months.
Momeni, 2010 (50)	Proteinuria at 2 and 4 months.
Muir, 2008 (51)	Change in Intercellular Adhesion Molecule-1, C-reactive protein, and IL-6 Levels at week 6.
NCT01078389, 2014 (52)	Mean Change From Baseline to Month 24 in the Modified

	Sharp/Van Der Heijde Erosion Score of the Single Affected Joint
NCT01350388 / Beddhu 2016 (53)	Change in Thiobarbituric Acid Reactive Substance (TBARS) Concentration in Adipose Tissue From Baseline to 24 Weeks
NCT01496469, 2015 (54)	Change From Baseline in 24-hour Mean Systolic Blood Pressure (SBP) Measured by Ambulatory Blood Pressure Monitoring at Week 6
NCT02128490 / Gunawardhana, 2016 (55)	Percentage of Participants With Serum Urate <5.0 mg/dL at Month 3
NCT02139046 / Saag, 2016 (56)	Percentage of Participants With Serum Urate <5.0 mg/dL at Month 3
Noman, 2010 (57)	Primary endpoint was the time to ST depression during exercise tolerance test at 6 weeks.
Parmley 1992 (58)	Infarct extension during the period of follow-up.
Poiley, 2016 (59)	Incidence of gout flares over 12 weeks.
Puntoni, 2013 (60)	Change of Ki-67 labeling index in adenomatous tissue at 4 weeks.
Rassi, 2007 (61)	Clearence of negativation serology for chagas disease over 2 years.
Rekhraj, 2013 (62)	Change in left ventricular mass accessed by CMR (cardiac magnetic resonance) imaging at 9 months.
Rentoukas, 2010 (63)	Effect on cardiac biomarkers, ST-E recovery, and major adverse cardiovascular events over 30 days.
Robertson, 2015 (64)	Change in exercise capacity on treadmill testing at 6 months.
Rosenfeld, 1974 (65)	Creatinine and creatinine clearence at the end of the study.

Saag, 2016 (66)	Change from baseline to month 12 in serum creatinine.
Sarris, 2007 (67)	Serum creatinine at 12 months.
Schumacher, 2008 (68)	Proportion of subjects with the last 3 monthly serum urate levels < 6.0 mg/dl at 28 weeks.
Segal, 2015 (69)	Change in clinic systolic blood pressure at 4 weeks.
Separham, 2016 (70)	Resolution of 50% of ST elevation 90 minutes after thrombolytic therapy.
Shen, 2010 (71)	Serum creatinine levels at the end of 12 months.
Shi, 2012 (72)	Change in renal function assessed as changes in eGFR at 6 months.
Sircar, 2015 (73)	Proportion of patients showing a 10% decline in estimated glomerular filtration rate (eGFR) from baseline at 6 months.
Siu, 2006(74)	Prevalence of stable kidney function with less than 40% increase in serum creatinine level at the end of 12 months.
Szwejkowski, 2013 (75)	Reduction in LVM as calculated by cardiac magnetic resonance imaging at baseline and at 9 months.
Taheraghdam, 2014 (76)	Functional outcome evaluated using a modified Rankin scale at the 3 months.
Takir, 2015 (77)	Improvement in insulin resistance defined by homeostatic model assessment of insulin resistance (HOMA-IR) at 3 months.
Tan, 2014 (78)	Changes in blood pressure, blood uric acid, hypersensitive c-reactive protein, bladder inhibition-C and glomerular filtration rate (eGFR) over 2 years.

Tanaka, 2015 (79)	Mean serum uric acid at 12 weeks.
Tani, 2015 (80)	Change in the plasma renin activity (PRA) and plasma aldosterone concentration at 6 months.
Togha, 2007 (81)	Mean number of seizures per month and duration of seizure attacks over 6 months.
Tsuruta, 2015 (82)	FMD (Flow-mediated dilation) after the 4-week study period.
Usharani, 2016 (83)	Absolute and mean percentage reduction in serum uric acid levels from baseline to the end of 24 weeks.
Wang, 2012 (84)	Change in brachial-ankle pulse wave velocity (baPWV) and ankle-brachial index (ABI) at 3 months.
Wang, 2015 (85)	ABI and baPWV at 3 months.
Weiser, 2012 (86)	Total PANSS score at week 8.
Weiser, 2014 (87)	Total YMRS score at 6 weeks.
Yin, 2015 (88)	Thrombosis in myocardial infarction (TIMI) score at 3 months.
Zhang, 2012 (89)	Incidence of cardiovascular events over 1 year.
Zhou, 2009 (90)	Serum creatinine and glomerular filtration rate at 6 months.
Ziaee, 2006 (91)	Change in total symptom score over 3 months.

REFERENCES

1. Akhondzadeh S, Milajerdi MR, Amini H, Moinalghorabaei M**.** A double-blind, randomized, placebo-controlled study of allopurinol as adjunctive treatment for acute mania in hospitalized bipolar patients. Br J Clin Pharmacol 2005;59:634.
2. Akhondzadeh S, Milajerdi MR, Amini H, Tehrani-Doost M**.** Allopurinol as an adjunct to lithium and haloperidol for treatment of patients with acute mania: a double-blind, randomized, placebo-controlled trial. Bipolar Disord 2006;8:485-9.
3. Becker MA, Schumacher HR, Wortmann RL, MacDonald PA, Palo WA, Eustace D, et al. Febuxostat, a novel nonpurine selective inhibitor of xanthine oxidase: a twenty-eight-day, multicenter, phase II, randomized, double-blind, placebo-controlled, dose-response clinical trial examining safety and efficacy in patients with gout. Arthritis Rheum; 2005:916-23.
4. Borgi L, McMullan C, Wohlhueter A, Curhan GC, Fisher ND, Forman JP**.** Effect of Uric Acid-Lowering Agents on Endothelial Function: A Randomized, Double-Blind, Placebo-Controlled Trial. Hypertension 2017;69:243-8.
5. Bowden RG, Shelmadine BD, Moreillon JJ, Deike E, Griggs JO, Wilson RL, et al. Effects of uric acid on lipid levels in CKD patients in a randomized controlled trial. Cardiovasc Res 2013;4:56-63.
6. Brunstein MG, Ghisolfi ES, Ramos FL, Lara DR**.** A clinical trial of adjuvant allopurinol therapy for moderately refractory schizophrenia. J Clin Psychiatry 2005;66:213-9.

7. Chen X, Ye M**.** Effects of allopurinol on cardiac function and exercise capacity in hyperuricemic patients with congestive heart failure. Chinese Journal of Clinical Rational Drug Use 2009;12:1-3.

8. Chen W, Zhou H**.** Effects of Lowering Serum Uric Acid Levels on Vascular Sclerosis in Hemodialysis Patients. Practical Clinical Medicine 2014;15:15-7.

9. Cingolani HE, Plastino JA, Escudero EM, Mangal B, Brown J, Perez NG**.** The effect of xanthine oxidase inhibition upon ejection fraction in heart failure patients: La Plata Study. J Card Fail 2006;12:491-8.

10. Dawson J, Quinn TJ, Harrow C, Lees KR, Walters MR**.** The effect of allopurinol on the cerebral vasculature of patients with subcortical stroke; a randomized trial. Br J Clin Pharmacol 2009;68:662-8.

11. Deng Y, Zhang P, Liu H, Jia Q**.** Observation on allopurinol in lowering blood uric acid for slowing the progression of chronic renal failure. Journal of Practical Medicine 2010;26:982-4.

12. Dickerson FB, Stallings CR, Origoni AE, Sullens A, Khushalani S, Sandson N, et al. A double-blind trial of adjunctive allopurinol for schizophrenia. Schizophr Res 2009;109:66-9.

13. Dogan A, Yarlioglues M, Kaya MG, Karadag Z, Dogan S, Ardic I, et al. Effect of long-term and high-dose allopurinol therapy on endothelial function in normotensive diabetic patients. Blood Press; 2011:182-7.

14. Eddeland A, Hedelin H. Effects of allopurinol (Zyloric(Reg.trademark)) in patients with long-term indwelling urethral catheters. J Int Med Res; 1983:116-9.

15. Ettinger B, Tang A, Citron JT, Livermore B, Williams T. Randomized trial of allopurinol in the prevention of calcium oxalate calculi. N Engl J Med 1986;315:1386-9.

16. Fan A, Berg A, Bresee C, Glassman LH, Rapaport MH. Allopurinol augmentation in the outpatient treatment of bipolar mania: a pilot study. Bipolar disord; 2012:206-10.

17. Feuerman EJ, Nir MA. Allopurinol in psoriasis - a double-blind study. Br J Dermatol 1973;89:83-6.

18. George J, Carr E, Davies J, Belch JJ, Struthers A. High-dose allopurinol improves endothelial function by profoundly reducing vascular oxidative stress and not by lowering uric acid. Circulation 2006;114:2508-16.

19. Gibson T, Rodgers V, Potter C, Simmonds HA. Allopurinol treatment and its effect on renal function in gout: a controlled study. Ann Rheum Dis 1982;41:59-65.

20. Givertz MM, Anstrom KJ, Redfield MM, Deswal A, Haddad H, Butler J, et al. Effects of Xanthine Oxidase Inhibition in Hyperuricemic Heart Failure Patients: The Xanthine Oxidase Inhibition for Hyperuricemic Heart Failure Patients (EXACT-HF) Study. Circulation; 2015:1763-71.

21. Goicoechea M, De Vinuesa SG, Verdalles U, Ruiz-Caro C, Ampuero J, Rincon A, et al. Effect of allopurinol in chronic kidney disease progression and cardiovascular risk. Clin J Am Soc Nephrol 2010;5:1388-93.

22. Goldfarb DS, MacDonald PA, Gunawardhana L, Chefo S, McLean L**.** Randomized controlled trial of febuxostat versus allopurinol or placebo in individuals with higher urinary uric acid excretion and calcium stones. Clin J Am Soc Nephrol; 2013:1960-7.

23. Greig D, Alcaino H, Castro PF, Garcia L, Verdejo HE, Navarro M, et al. Xanthine-oxidase inhibitors and statins in chronic heart failure: effects on vascular and functional parameters. J Heart Lung Transplant 2011;30:408-13.

24. Guo J, Ni X, Shi L, Liu Ji, Wu X, Wei W**.** Clinical effect of allopurinol in the treatment of acute cerebral infarction combined with hyperuricemia. China Modern Medicine 2015;22:114-6.

25. Guo J, Ni X, Shi L, Liu Ji, Wu X, Wei W**.** Effect of Allopurinol on the Prognosis in Patients with Acute Ischemic Stroke Complicated with Hyperuricemia. Research of Traditional Chinese Medicine 2016;29:110-1.

26. Hare JM, Mangal B, Brown J, Fisher C, Freudenberger R, Colucci WS, et al. Impact of oxypurinol in patients with symptomatic heart failure. Results of the OPT-CHF study. J Am Coll Cardiol 2008;51:2301-9.

27. Higgins P, Walters MR, Murray HM, McArthur K, McConnachie A, Lees KR, et al. Allopurinol reduces brachial and central blood pressure, and carotid intima-media thickness progression after ischaemic stroke and transient ischaemic attack: A randomised controlled trial. Heart 2014;100:1085-92.

28. Hill E, Higgs JB, Sky K, Sit M, Collamer AN, Author A, et al. Does starting allopurinol prolong acute treated gout? Arthritis Rheum 2013;65(Suppl 10):S730.

29. Hosoya T, Ohno I, Nomura S, Hisatome I, Uchida S, Fujimori S, et al. Effects of topiroxostat on the serum urate levels and urinary albumin excretion in hyperuricemic stage 3 chronic kidney disease patients with or without gout. Clin Exp Nephrol 2014;18:876-84.

30. Hosoya T, Sasaki T, Hashimoto H, Sakamoto R, Ohashi T**.** Clinical efficacy and safety of topiroxostat in Japanese male hyperuricemic patients with or without gout: an exploratory, phase 2a, multicentre, randomized, double-blind, placebo-controlled study. J Clin Pharm Ther 2016;41:298-305.

31. Hosoya T, Sasaki T, Ohashi T**.** Clinical efficacy and safety of topiroxostat in Japanese hyperuricemic patients with or without gout: a randomized, double-blinded, controlled phase 2b study. Clin Rheumatol 2016;36:649-56.

32. Jahangard L, Soroush S, Haghighi M, Ghaleiha A, Bajoghli H, Holsboer-Trachsler E, et al. In a double-blind, randomized and placebo-controlled trial, adjuvant allopurinol improved symptoms of mania in in-patients suffering from bipolar disorder. Eur Neuropsychopharmacol 2014;24:1210-21.

33. Jalal DI, Decker E, Perrenoud L, Nowak KL, Bispham N, Mehta T, et al. Vascular Function and Uric Acid-Lowering in Stage 3 CKD. J Am Soc Nephrol 2016;28(Suppl 3):943-52.

34. Jalalzadeh M, Nurcheshmeh Z, Mohammadi R, Mousavinasab N, Ghadiani MH**.** The effect of allopurinol on lowering blood pressure in hemodialysis patients with hyperuricemia. J Res Med Sci 2012;17:1039-46.

35. Jarnerot G, Strom M, Danielsson A, Kilander A, Loof L, Hultcrantz R, et al. Allopurinol in addition to 5-aminosalicylic acid based drugs for the maintenance treatment of ulcerative colitis. Aliment Pharmacol Ther 2000;14:1159-62.

36. Jitapunkul S, Chalaprawat M, Bunnag S, Bhuvapanich S, Kangkaya V, Pasatrat S, et al. The relationship between glucose and uric acid metabolism: influence of short term allopurinol on glucose metabolism. J Med Assoc Thai 1991;74:80-6.

37. Joelsson M, Andersson M, Bark T, Gullberg K, Hallgren T, Jiborn H, et al. Allopurinol as prophylaxis against pouchitis following ileal pouch-anal anastomosis for ulcerative colitis: A randomized placebo-controlled double-blind study. Scand J Gastroenterol 2001;36:1179-84.

38. Kamatani N, Fujimori S, Hada T, Hosoya T, Kohri K, Nakamura T, et al. Placebo-controlled double-blind dose-response study of the non-purine-selective xanthine oxidase inhibitor febuxostat (TMX-67) in patients with hyperuricemia (including gout patients) in japan: late phase 2 clinical study. J Clin Rheumatol 2011;17:S35-43.

39. Kamatani N, Fujimori S, Hada T, Hosoya T, Kohri K, Nakamura T, et al. Placebo-Controlled, Double-Blind Study of the Non-Purine-Selective Xanthine Oxidase Inhibitor Febuxostat (TMX-67) in Patients With Hyperuricemia Including Those With Gout in Japan: phase 3 clinical study. J Clin Rheumatol 2011;17:S19-S26.

40. Kanbay M, Huddam B, Azak A, Solak Y, Kadioglu GK, Kirbas I, et al. A randomized study of allopurinol on endothelial function and estimated glomular filtration rate in asymptomatic hyperuricemic subjects with normal renal function. Clin J Am Soc Nephrol 2011;6:1887-94.

41. Kao MP, Ang DS, Gandy SJ, Nadir MA, Houston JG, Lang CC, et al. Allopurinol benefits left ventricular mass and endothelial dysfunction in chronic kidney disease. J Am Soc Nephrol 2011;22:1382-9.

42. Khan F, George J, Wong K, McSwiggan S, Struthers AD, Belch JJ**.** Allopurinol treatment reduces arterial wave reflection in stroke survivors. Cardiovasc Ther; 2008:247-52.

43. Lei J, Li S. Clinical research on allopurinol lowering of uric acid level of chronic renal disease peciant for the delay of the progression of renal disease. Shaanxi Medical Journal 2009;38:1191-2.

44. Liu J, Sheng D**.** Allopurinol in lowering serum uric acid level for the delay of the progression of chronic renal disease. China Pharmacy 2007;18:2524-5.

45. Liu P, Wang H, Zhang F, Chen Y, Wang D, Wang Y**.** The Effects of Allopurinol on the Carotid Intima-media Thickness in Patients with Type 2 Diabetes and Asymptomatic Hyperuricemia: A Three-year Randomized Parallel-controlled Study. Intern Med 2015;54:2129-37.

46. Machado-Vieira R, Soares JC, Lara DR, Luckenbaugh DA, Busnello JV, Marca G, et al. A double-blind, randomized, placebo-controlled 4-week study on the efficacy and safety of the purinergic agents allopurinol and dipyridamole adjunctive to lithium in acute bipolar mania. J Clin Psychiatry 2008;69:1237-45.

47. Madero M, Rodriguez Castellanos FE, Jalal D, Villalobos-Martin M, Salazar J, Vazquez-Rangel A, et al. A pilot study on the impact of a low fructose diet and allopurinol on clinic blood pressure among overweight and prehypertensive subjects: a randomized placebo controlled trial. J Am Soc Hypertens 2015;9:837-44.

48. Mao L**.** Prospective study of influence of blood uric acid and intervention treatment on coronary heart disease in rural area of Nanhai. China Modern Doctor 2015;53:8-11.

49. Modabber A, Najmedin ST**.** Allopurinol as an adjuvant therapy for refractory schizophrenia. Pak J Med Sci 2009;25:591-6.

50. Momeni A, Shahidi S, Seirafian S, Taheri S, Kheiri S**.** Effect of Allopurinol in Decreasing Proteinuria in Type 2 Diabetic Patients. Iran J Kidney Dis 2010;4:128-32.

51. Muir SW, Harrow C, Dawson J, Lees KR, Weir CJ, Sattar N, et al. Allopurinol Use Yields Potentially Beneficial Effects on Inflammatory Indices in Those With Recent Ischemic Stroke A Randomized, Double-Blind, Placebo-Controlled Trial. Stroke 2008;39:3303-7.

52. Takeda Pharmaceuticals. Effect of Febuxostat on Joint Damage in Hyperuricemic Subjects With Early Gout. In: ClinicalTrials.gov [Internet]. Bethesda (MD): National Library of Medicine (US). 2000- [cited 2016 Dec 27]. Available from: https://clinicaltrials.gov/ct2/show/NCT01078389?term=nct01078389&rank=1 NLM Identifier: NCT01078389.

53. Beddhu S, Filipowicz R, Wang B, Wei G, Chen X, Roy AC, et al. A Randomized Controlled Trial of the Effects of Febuxostat Therapy on Adipokines and Markers of Kidney Fibrosis in Asymptomatic Hyperuricemic Patients With Diabetic Nephropathy. Can J Kidney Health Dis. 2016;5;3:2054358116675343. doi: 10.1177/2054358116675343. Also available from: https://clinicaltrials.gov/ct2/show/NCT01350388?term=nct01350388&rank=1.

54. Takeda Pharmaceuticals. Effect of Febuxostat on Blood Pressure. In: ClinicalTrials.gov [Internet]. Bethesda (MD): National Library of Medicine (US). 2000- [cited 2016 Dec 27]. Available from: https://clinicaltrials.gov/ct2/show/NCT01496469?term=nct01496469&rank=1 NLM Identifier: NCT01496469.

55. Takeda Pharmaceuticals. Efficacy and Safety of Extended Release and Immediate Release Febuxostat in Participants With Gout and Moderate Renal Impairment. In: ClinicalTrials.gov [Internet]. Bethesda (MD): National Library of Medicine (US). 2000- [cited 2016 Dec 27]. Available from: https://clinicaltrials.gov/ct2/show/NCT02128490?term=nct02128490&rank=1 NLM Identifier: NCT02128490.

.

56. Takeda Pharmaceuticals. Efficacy and Safety of Extended Release and Immediate Release Febuxostat in Participants With Gout. In: ClinicalTrials.gov [Internet]. Bethesda (MD): National Library of Medicine (US). 2000- [cited 2016 Dec 27]. Available from: https://clinicaltrials.gov/ct2/show/NCT02139046?term=nct02139046&rank=1 NLM Identifier: NCT02139046.

57. Noman A, Ang DSC, Ogston S, Lang CC, Struthers AD**.** Effect of high-dose allopurinol on exercise in patients with chronic stable angina: a randomised, placebo controlled crossover trial. Lancet 2010;375:2161-7.

58. Parmley LF, Mufti AG, Downey JM**.** Allopurinol therapy of ischemic heart disease with infarct extension. Can J Cardiol 1992;8:280-6.

59. Poiley J, Steinberg AS, Choi YJ, Davis CS, Martin RL, McWherter CA, et al. A Randomized, Double-Blind, Active- and Placebo-Controlled Efficacy and Safety Study of Arhalofenate for Reducing Flare in Patients With Gout. Arthritis Rheumatol 2016;68:2027-34.

60. Puntoni M, Branchi D, Argusti A, Zanardi S, Crosta C, Meroni E, et al. A Randomized, Placebo-Controlled, Preoperative Trial of Allopurinol in Subjects with Colorectal Adenoma. Cancer Prev Res 2013;6:74-81.

61. Rassi A, Luquetti AO, Rassi Jr A, Rassi GG, Rassi SG, Da Silva IG, et al. Specific treatment for Trypanosoma cruzi: Lack of efficacy of allopurinol in the human chronic phase of chagas disease. Am J Trop Med Hyg 2007;76:58-61.

62. Rekhraj S, Gandy SJ, Szwejkowski BR, Nadir MA, Noman A, Houston JG, et al. High-Dose Allopurinol Reduces Left Ventricular Mass in Patients With Ischemic Heart Disease. J Am Coll Cardiol 2013;61:926-32.

63. Rentoukas E, Tsarouhas K, Tsitsimpikou C, Lazaros G, Deftereos S, Vavetsi S**.** The prognostic impact of allopurinol in patients with acute myocardial infarction undergoing primary percutaneous coronary intervention. Int J Cardiol 2010;145:257-8.

64. Robertson AJ, Struthers AD**.** A Randomized Controlled Trial of Allopurinol in Patients With Peripheral Arterial Disease. Can J Cardiol 2015;32:190-6.

65. Rosenfeld JB**.** Effect of long-term allopurinol administration on serial GFR in normotensive and hypertensive hyperuricemic subjects. Adv Exp Med Biol 1974;41:581-96.

66. Saag KG, Whelton A, Becker MA, MacDonald P, Hunt B, Gunawardhana L**.** Impact of Febuxostat on Renal Function in Gout Patients With Moderate-to-Severe Renal Impairment. Arthritis Rheumatol 2016;68:2035-43.

67. Sarris E, Bagiatudi G, Stavrianaki D, Salpigidis K, M. S**.** Use of allopurinol in slowing the progression of chronic renal disease (abstract). Nephrol Dial Transplant 2007;47:vi61.

68. Schumacher HR, Jr., Becker MA, Wortmann RL, MacDonald PA, Hunt B, Streit J, et al. Effects of Febuxostat Versus Allopurinol and Placebo in Reducing Serum Urate in Subjects With Hyperuricemia and Gout: A 28-Week, Phase III, Randomized, Double-Blind, Parallel-Group Trial. Arthritis Care Res 2008;59:1540-8.

69. Segal MS, Srinivas TR, Mohandas R, Shuster JJ, Wen X, Whidden E, et al. The effect of the addition of allopurinol on blood pressure control in African Americans treated with a thiazide-like diuretic. J Am Soc Hypertens; 2015:610-9.

70. Separham A, Ghaffari S, Najafi H, Ghaffari R, Ziaee M, Babaei H**.** The Impact of Allopurinol on Patients With Acute ST Elevation Myocardial Infarction Undergoing Thrombolytic Therapy. J Cardiovasc Pharmacol 2016;68:265-8.

71. Shen H, Liu D**.** Clinical research on allopurinol in lowering serum uric acid level for the delay of the progression of chronic renal disease. China Foreign Medical Treatment 2010;12:88–9.

72. Shi Y, Chen W, Jalal D, Li Z, Chen W, Mao H, et al. Clinical outcome of hyperuricemia in IgA nephropathy: a retrospective cohort study and randomized controlled trial. Kidney Blood Press Res 2012;35:153-60.

73. Sircar D, Chatterjee S, Waikhom R, Golay V, Raychaudhury A, Chatterjee S, et al. Efficacy of Febuxostat for Slowing the GFR Decline in Patients With CKD and Asymptomatic Hyperuricemia: A 6-Month, Double-Blind, Randomized, Placebo-Controlled Trial. Am J Kidney Dis 2015;66:945-50.

74. Siu YP, Leung KT, Tong MK, Kwan TH**.** Use of allopurinol in slowing the progression of renal disease through its ability to lower serum uric acid level. Am J Kidney Dis 2006;47:51-9.

75. Szwejkowski BR, Gandy SJ, Rekhraj S, Houston JG, Lang CC, Morris AD, et al. Allopurinol Reduces Left Ventricular Mass in Patients With Type 2 Diabetes and Left Ventricular Hypertrophy. J Am Coll Cardiol 2013;62:2284-93.

76. Taheraghdam AA, Sharifipour E, Pashapour A, Namdar S, Hatami A, Houshmandzad S, et al. Allopurinol as a Preventive Contrivance after Acute Ischemic Stroke in Patients with a High Level of Serum Uric Acid: A Randomized, Controlled Trial. Med Princ Pract 2014;23:134-9.

77. Takir M, Kostek O, Ozkok A, Elcioglu OC, Bakan A, Erek A, et al. Lowering Uric Acid With Allopurinol Improves Insulin Resistance and Systemic Inflammation in Asymptomatic Hyperuricemia. J Investig Med 2015;63:924-9.

78. Tan Y, Weinan D. Effect of Allopurinol in Chronic Kidney Disease Progression and Cardiovascular Risk. Journal of Liaoning Medical University 2014;35:17-9.

79. Tanaka K, Nakayama M, Kanno M, Kimura H, Watanabe K, Tani Y, et al. Renoprotective effects of febuxostat in hyperuricemic patients with chronic kidney disease: a parallel-group, randomized, controlled trial. Clin Exp Nephrol 2015;19:1044-53.

80. Tani S, Nagao K, Hirayama A. Effect of Febuxostat, a Xanthine Oxidase Inhibitor, on Cardiovascular Risk in Hyperuricemic Patients with Hypertension: A Prospective, Open-label, Pilot Study. Clin Drug Investig 2015;35:823.

81. Togha M, Akhondzadeh S, Motamedi M, Ahmadi B, Razeghi S. Allopurinol as adjunctive therapy in intractable epilepsy: a double-blind and placebo-controlled trial. Arch Med Res 2007;38:313-6.

82. Tsuruta Y, Kikuchi K, Tsuruta Y, Sasaki Y, Moriyama T, Itabashi M, et al. Febuxostat improves endothelial function in hemodialysis patients with hyperuricemia: A randomized controlled study. Hemodial Int 2015;19:514-20.

83. Usharani P, Nutalapati C, Pokuri VK, Kumar CU, Taduri G. A randomized, double-blind, placebo-, and positive-controlled clinical pilot study to evaluate the efficacy and tolerability of standardized aqueous extracts of Terminalia chebula and Terminalia bellerica in subjects with hyperuricemia. Clin Pharmacol 2016;8:51-9.
84. Wang X, Gao Y, An Y. Short-time impact of allopurinol on arterial elasticity in patients with hyperuricemia. . Journal of Shandong University (Health Sciences) 2012;50:1–5.
85. Wang X, Pang Z. Impact of Allopurinol on Atherosclerosis Complicated with Hyperuricemia. Practical Journal of Cardiac Cerebral Pneumal and Vascular Disease 2015;23:20-3.
86. Weiser M, Gershon AA, Rubinstein K, Petcu C, Ladea M, Sima D, et al. A randomized controlled trial of allopurinol vs. placebo added on to antipsychotics in patients with schizophrenia or schizoaffective disorder. Schizophr Res 2012;138:35-8.
87. Weiser M, Burshtein S, Gershon AA, Marian G, Vlad N, Grecu IG, et al. Allopurinol for mania: a randomized trial of allopurinol versus placebo as add-on treatment to mood stabilizers and/or antipsychotic agents in manic patients with bipolar disorder. Int J Bipolar Disorder 2014;16:441-7.
88. Yin G, Han N, Li Y. Effect of allopurinol on unstable angina pectoris complicated with hyperuicemia. Journal of Practical Diagnosis and Therapy 2015;29:93-5.

89. Zhang J**.** The effect of intervene allopurinol of hyperuricemia and hyperlipidemia of patient with acute coronary syndrome prognosis. Chinese Journal of Clinical Rational Drug Use 2012;5:5-6.

90. Zhou DY, Zhao YJ, Xiao X**.** Treatment of hyperuricemia in chronic kidney disease patients and its effect. . Mod Med J China 2009;11:36-9.

91. Ziaee AM, Akhavizadegan H, Karbakhsh M**.** Effect of allopurinol in chronic nonbacterial prostatitis: a double blind randomized clinical trial. Int Braz J Urol 2006;32:181-6.
