# Supplementary material for: Xanthine oxidase inhibitors for prevention of cardiovascular events: a systematic review and meta-analysis of randomized controlled trials
Source: BMC Cardiovasc Disord. 2018 Feb 7;18:24. doi: 10.1186/s12872-018-0757-9 (PMC5804046; doi:10.1186/s12872-018-0757-9)
Supplement: Supplementary file 3 — Supplementary file containing description of studies excluded from the meta-analysis after evaluation in full-text format. (PDF 410 kb) [file 12872_2018_757_MOESM3_ESM.pdf]

Additional File 3

Bredemeier et al. Xanthine Oxidase Inhibitors for Prevention of Cardiovascular Events: A Systematic Review and Meta-Analysis of Randomized Controlled Trials. BMC Cardiovascular Disorders.

DESCRIPTION OF STUDIES EXCLUDED FROM THE META-ANALYSIS AFTER EVALUATION IN FULL-TEXT FORMAT

1) Initial systematic literature search from inception to Sep 29, 2014 (subtitles represent the reasons for exclusion).

Absence of placebo or no-treatment group

1. Becker MA, Schumacher HR, MacDonald PA, Lioyd EJ, Lademacher C, Joseph-Ridge N. Orate-lowering therapy in subjects with gout: Interim results from the febuxostat/allopurinol comparative extension long-term study (EXCEL). Annals of the Rheumatic Diseases 2007;66:230-1.

2. Bosmansky K, Trnavsky K. Evaluation of the therapy effects of Acidum tienilicum, Benzbromaron and Allopurinol in gout patients. Zeitschrift fur Rheumatologie; 1982:165.

3. Calvo C, Lopez Paz JE, Hermida Ameijeiras A, Pena Seijo M, Calvo Gonzalez G, Romero Miguez ML. Hyperuricemia, cronotherapy with alopurinol and hypertension [abstract]. Journal of hypertension; 2010:e541.

4. Chocair P, Duley J, Simmonds HA, Cameron JS, Ianhez L, Arap S, et al. Low-dose allopurinol plus azathioprine/cyclosporin/prednisolone, a novel immunosuppressive regimen. Lancet; 1993:83-4.

5. Chocair PR, Duley JA, Cameron JS, Arap S, Ianhez L, Sabbaga E, et al. Does low-dose allopurinol, with azathioprine, cyclosporin and prednisolone, improve renal transplant immunosuppression? Advances in Experimental Medicine and Biology 1995;370:205-8.

6. Das VN, Ranjan A, Sinha AN, Verma N, Lal CS, Gupta AK, et al. A randomized clinical trial of low dosage combination of pentamidine and allopurinol in the treatment of antimony unresponsive cases of visceral leishmaniasis. J Assoc Physicians India 2001;49:609-13.

7. Kumar B, Agarwal PK. Comparative evaluation of efficacy and safety profile of febuxostat with allopurinol in patients with hyperuricemia and gout. International Journal of Pharma Medicine and Biological Sciences; 2013:52-6.

8. Kuzell WC, Seebach LM, Glover RP, Jackman AE. Treatment of gout with allopurinol and sulphinpyrazone in combination and with allopurinol alone. Ann Rheum Dis 1966;25(6 Suppl):634-42.

9. Landmesser U, Spiekermann S, Preuss C, Sorrentino S, Fischer D, Manes C, et al. Angiotensin II induces endothelial xanthine oxidase activation: Role for endothelial dysfunction in patients with coronary disease. Arteriosclerosis, Thrombosis, and Vascular Biology 2007;27(4):943-8.

10. Liu C, Zhao Q, Zhen Y, Gao Y, Tian L, Wang L, et al. Prednisone in Uric Acid lowering in Symptomatic Heart Failure Patients With Hyperuricemia (PUSH-PATH) study. The Canadian journal of cardiology; 2013:1048-54.

11. MacDonald TM, Ford I, Nuki G, Mackenzie IS, De Caterina R, Findlay E, et al. Protocol of the Febuxostat versus Allopurinol Streamlined Trial (FAST): a large prospective, randomised, open, blinded endpoint study comparing the cardiovascular safety of allopurinol and febuxostat in the management of symptomatic hyperuricaemia. Bmj Open 2014;4(7).

12. Marwaha V, Singh B, Dawra S, Behl R, Singh H, Narayanan CS, et al. A clinical study of patients of chronic topheceous gout with special reference to response to therapy. Indian Journal of Rheumatology 2010;5(3 SUPPL. 1):S29.

13. Matzkies F. [Long lasting normalization of uric acid after combination therapy with 300 mg allopurinol and 60 mg benzbromarone in patients with gout and hyperuricemia]. Med Klin (Munich) 1992;87(9):460-2.

14. Merimsky O, Inbar M, Chaitchik S. Treatment of advanced colorectal cancer by 5-fluorouracil-leucovorin combination with or without allopurinol: a prospective randomized study. Anti-cancer drugs; 1991:447-51.

15. Mitha E, Schumacher HR, Fouche L, Luo S-F, Weinstein SP, Yancopoulos GD, et al. Rilonacept for gout flare prevention during initiation of uric acid-lowering therapy: results from the PRESURGE-2 international, phase 3, randomized, placebo-controlled trial. Rheumatology 2013;52(7):1285-92.

16. Nasr G, Maurice C. Allopurinol and global left myocardial function in heart failure patients. J Cardiovasc Dis Res 2010;1(4):191-5.

17. Nyfos L. [Treatment of the gouty diathesis with a xanthine oxidase inhibitor. A clinical trial of allopurinol]. Ugeskr Laeger 1967;129(8):253-7.

18. O'Driscoll JG, Green DJ, Rankin JM, Taylor RR. Nitric oxide-dependent endothelial function is unaffected by allopurinol in hypercholesterolaemic subjects. Clinical and Experimental Pharmacology and Physiology 1999;26(10):779-83.

19. Okada Y, Nonomura M, Takeuchi H, Kawamura J, Yoshida O. [Experimental and clinical studies on calcium lithiasis. II. Prevention of recurrent calcium stones with thiazides and allopurinol]. Hinyokika Kiyo 1986;32(9):1247-57.

20. Perez-Ruiz F, Calabozo M, Fernandez-Lopez MJ, Herrero-Beites A, Ruiz-Lucea E, Garcia-Erauskin G, et al. Treatment of chronic gout in patients with renal function impairment: An open, randomized, actively controlled study. Journal of Clinical Rheumatology 1999;5(2):49-55.

21. Pergament E, Christiansen N, DeBerry M. Inefficacy of allopurinol in treatment of Duchenne muscular dystrophy. Current therapeutic research, clinical and experimental; 1983:832-8.

22. Salim AS. Oxygen-derived free radical scavengers protect patients against the complications of erosive gastritis. Intensive care medicine; 1992:61-2.

23. Schumacher HR, Becker MA, Lloyd E, MacDonald PA, Lademacher C. Febuxostat in the treatment of gout: 5-yr findings of the FOCUS efficacy and safety study. Rheumatology (Oxford, England); 2009:188-94.

24. Sies CW, Florkowski CM, Frampton CM, O'Donnell JL, Chapman PT, Stamp LK. Comparison of two plasma urate assays in patients receiving vitamin C supplementation. Pathology 2014;46(4):333-5.

25. Singal KK, Goyal S, Gupta P, Aggawal BK, Author A, Dept. of Medicine MMI, et al. Comparison between Allopurinol and Febuxostat in management of gout patients - A prospective study. Bangladesh Journal of Medical Science 2011;10(4):257-9.

26. Smyth CJ. Studies with allopurinol (HPP) in patients with tophaceous gout. Arthritis Rheum 1965;8(5):907-10.

27. Stamp LK, O'Donnell JL, Frampton C, Drake JM, Zhang M, Chapman PT. Clinically Insignificant Effect of Supplemental Vitamin C on Serum Urate in Patients With Gout: A Pilot Randomized Controlled Trial. Arthritis and Rheumatism 2013;65(6):1636-42.

28. Sziegoleit W, Mann D, Forster W, Crodel HW, Graupner S, Gutschker A, et al. MULTICENTRIC THERAPY STUDY WITH ALLOPURINOL AND TALINOLOL IN COMPARISON TO

PLACEBO IN PATIENTS WITH ANGINA-PECTORIS. Deutsche Gesundheitswesen-Zeitschrift Fur Klinische Medizin 1981;36(7):321-5.

29. Tobaigy M, Mejer J, Peitersen B, Ansari M, Poll P. A comparative study of allopurinol and pentostam in the treatment of visceral leishmaniasis. Advances in experimental medicine and biology; 1986:471-3.

30. Torres RJ, Prior C, Puig JG, Author A, Division of C, Biochemistry LPUHMS, et al. Efficacy and safety of allopurinol in patients with hypoxanthine-guanine phosphoribosyltransferase deficiency. Metabolism: Clinical and Experimental 2007;56(9):1179-86.

31. Tousoulis D, Andreou I, Tsiatas M, Miliou A, Tentolouris C, Siasos G, et al. Effects of rosuvastatin and allopurinol on circulating endothelial progenitor cells in patients with congestive heart failure: the impact of inflammatory process and oxidative stress. Atherosclerosis 2011;214(1):151-7.

32. Viglioglia PA, Plante GE, Viglioglia J, Saraceno EF. Allopurinol in psoriasis. Dermatologica 1970;141(3):203-7.

33. Wang YF, Li BH, Zhang M, Xu WB, Zhou R, Zhou M, et al. Stage-based treatment of gouty arthritis by combination therapy of traditional Chinese and Western medicines: A randomized controlled trial. Journal of Chinese Integrative Medicine 2008;6(6):576-80.

34. Xie JY, Wang L, Li QX, Li XM. [Study on mechanisms of electroacupuncture treatment of acute gouty arthritis]. Zhongguo Zhen Jiu 2007;27(12):898-900.

35. Yamamoto T, Moriwaki Y, Takahashi S, Tsutsumi Z, Yamakita J, Higashino K. Effect of allopurinol and benzbromarone on the concentration of uridine in plasma. Metabolism 1997;46(12):1473-6.

36. Yokomizo H, Yoshimatsu K, Hashimoto M, Ishibashi K, Umehara A, Yoshida K, et al. Prophylactic Efficacy of Allopurinol Ice Ball for Leucovorin/ 5-Fluorouracil Therapy-induced Stomatitis. Anticancer Research 2004;24(2 C):1131-4.

37. Zulantay I, Apt W, Rodriguez J, Venegas J, Sanchez G. Serologic evaluation of treatment of chronic Chagas disease with allopurinol and itraconazole. Revista Medica De Chile 1998;126(3):265-70.

Study protocol

1. Freudenberg RS, Schwarz Jr RP, Brown J, Moore A, Mann D, Givertz MM, et al. Rationale, design and organisation of an efficacy and safety study of oxypurinol added to standard therapy in patients with NYHA class III - IV congestive heart failure. Expert Opinion on Investigational Drugs 2004;13(11):1509-16.

2. Hosoya T, Kimura K, Itoh S, Inaba M, Uchida S, Tomino Y, et al. The effect of febuxostat to prevent a further reduction in renal function of patients with hyperuricemia who have never had gout and are complicated by chronic kidney disease stage 3: study protocol for a multicenter randomized controlled study. Trials 2014;15.

Publication not obtained

1. Bailey RR, Author A, Christchurch Hosp C, Correspondence A, Christchurch Hosp C. Allopurinol in calcium stone disease. Current Therapeutics 1978;19(6):13-5.

2. Emmerson BT. The use of the xanthine oxidase inhibitor, allopurinol, in the control of hyperuricaemia, gout and uric acid calculi. Australas Ann Med 1967;16(3):205-14.

3. Eyman E, Brzozowska-Jurkowska A, Werynska-Przybylska J. [Clinical evaluation of Milurit]. Reumatologia 1973;11(3):299-304.

4. Kalyakin IE, Mitkin AF. Effects of allopurinol on uric acid metabolism and lipid peroxidation in IHD patients with stable angina pectoris. Kardiologiya; 1993:15-7.

Inclusion of individuals <18 years-old

1. Allopurinol reduces blood pressure in hypertensive youths AUTHOR ADDRESSES SOURCE Australian Journal of Pharmacy (2009) 90:1067 (76-77). Date of Publication: April 2009.

2. Apt W, Aguilera X, Arribada A, Perez C, Miranda C, Zulantay I, et al. TREATMENT OF CHRONIC HUMAN CHAGAS-DISEASE WITH ITRACONAZOLE AND ALLOPURINOL - PRELIMINARY-REPORT. Revista Medica De Chile 1994;122(4):420-7.

3. El-Samahy MH, Elbarbary NS, Afify MAA, Sallam DE, Author A, Ain S, et al. Effect of allopurinol versus angiotensin converting enzyme inhibitors in decreasing microalbuminuria in type I diabetic patients. Pediatric Diabetes 2014;15 SUPPL. 19:84.

4. Ginella A, Holzman A, Iihoshi N, Barja Z, Peredo C. Eficacia del Alopurinol en la Enfermedad de Chagas Cronica. Resultados del estudio realizado en Santa Cruz, Bolivia. Efficacy of the Allopurinol in chronic Chagas disease. Bol. cient. CENETROP;16(1):25-9.

5. Griffiths RD, Cady E, Edwards RHT, Wilkie DR. 31 PHOSPHORUS NUCLEAR MAGNETIC-RESONANCE USED IN A DOUBLE-BLIND TRIAL OF ALLOPURINOL IN DUCHENNE MUSCULAR-DYSTROPHY. Clinical Science 1984;66(2):P20-P.

6. Horsfall PA, Plummer J, Allan WG, Girling DJ, Nunn AJ, Fox W. Double blind controlled comparison of aspirin, allopurinol and placebo in the management of arthralgia during pyrazinamide administration. Tubercle; 1979:13-24.

7. Martinez S, Marr JJ. Allopurinol in the treatment of American cutaneous leishmaniasis. New England journal of medicine; 1992:741-4.

8. Miranda ME. The influence of allopurinol on renal deterioration in familial nepropathy associated with hyperuricemia (FNAH). The Spanish Group for the Study of FNAH. Adv Exp Med Biol 1994;370:61-4.

9. Nct. Randomized Double-blinded, Placebo-controlled, Cross-over Trial of Allopurinol for the Treatment of Newly Diagnosed Essential Hypertension in Adolescents. ClinicalTrials.gov [http://clinicaltrials.gov]; 2003.

10. Smith CR. Possible cardioprotective effects of allopurinol. Am J Prev Med 1988;4(2 Suppl):33-8.

11. Sonoda T, Koide T, Oka T, Sakaguchi H, Itatani H, Ohkawa T, et al. [A study of allopurinol in the prevention of recurrent calcium oxalate stones]. Hinyokika Kiyo 1985;31(11):2071-9.

Different publications of studies already evaluated

1. Akhondzadeh S, Milajerdi MR, Amini H, Moinalghorabaei M. A double-blind, randomized, placebo-controlled study of allopurinol as adjunctive treatment for acute mania in hospitalized bipolar patients. British Journal of Clinical Pharmacology 2005;59(5):634-.

2. Akhondzadeh S, Safarcherati A, Amini H. Beneficial antipsychotic effects of allopurinol as add-on therapy for schizophrenia: a double blind, randomized and placebo controlled trial. Progress in Neuro-Psychopharmacology & Biological Psychiatry 2005;29(2):253-9.

3. Becker MA, Schumacher H, Wortmann RL, Joseph-Ridge N, Lademacher C. A safety and efficacy clinical trial of a novel nun-purine selective inhibitor of xanthine oxidase, febuxostat in subjects with gout. Annals of the Rheumatic Diseases 2004;63:60-.

4. Brunstein MG, Ghisolfi ES, Ramos FLP, Lara DR. Clinical trial of allopurinol adjuvant therapy for poorly responsive schizophrenia. Schizophrenia Research 2004;67(1):142-3.

5. Dawson J, Quinn TJ, Harrow C, Lees KR, Walters MR. The effect of allopurinol on the cerebral vasculature of patients with subcortical stroke; a randomized trial. British Journal of Clinical Pharmacology 2009;68(5):662-8.

6. Farquharson CAJ, Butler R, Hill A, Belch JJF, Struthers AD. Endothelial vasodilator dysfunction in chronic heart failure patients is improved by allopurinol [abstract]. Clinical science; 1999:2p-3p.

7. Gibson T, Simmonds HA, Potter C, Rogers V. A controlled study of the effect of long term allopurinol treatment on renal function in gout. Adv Exp Med Biol 1980;122a:257-62.

8. Goldfarb DS, MacDonald P, Hunt B, Gunawardhana L, Author A, New York Vamc NYULMCNYUS, et al. Febuxostat in gout: Serum urate responses in uric acid overproducers vs. underexcretors. American Journal of Kidney Diseases 2010;55(4):A59.

9. Goldfarb DS, MacDonald PA, Gunawardhana L, Chefo S, McLean L, Author A, et al. Prevention of recurrent calcium stones in subjects with hyperuricosuria: A randomized controlled trial of febuxostat vs allopurinol. Arthritis and Rheumatism 2012;64 SUPPL. 10:S67.

10. Harrow CJ, Muir SW, Lees KR, Walters MR. Allopurinol's effect on intercellular adhesion molecule 1 (ICAM) and inflammatory cytokines after ischaemic stroke: a pilot study. Cerebrovascular diseases (Basel, Switzerland); 2006:131-2.

11. Higgins P, Walters MR, Lees KR, McConnachie A, Murray H, McArthur K. Allopurinol reduces carotid artery intima-media thickness progression amongst stroke survivors. Cerebrovascular diseases (Basel, Switzerland); 2013:578.

12. Joseph-Ridge N. Phase II, dose-response, safety and efficacy clinical trial of a new oral xanthine oxidase inhibitor TMX-67 (febuxostat) in subjects with gout. Arthritis and Rheumatism 2002;46(9):S142-S.

13. Kao MP, Ang DS, Gandy SJ, Lang CC, Struthers AD, Author A, et al. Allopurinol reduces both left ventricular hypertrophy and endothelial dysfunction in patients with chronic kidney disease. NDT Plus 2010;3 SUPPL. 3:iii302.

14. Kao MP, Ang DS, Gandy SJ, Nadir A, Lang CC, Struthers AD, et al. Allopurinol reduces both left ventricular hypertrophy and endothelial dysfunction in cardiorenal patients. European Heart Journal 2010;31 SUPPL. 1:164.

Intervention and control groups differ not only on use of XOI

1. Guderian RH, Chico ME, Rogers MD, Pattishall KM, Grogl M, Berman JD. PLACEBO CONTROLLED TREATMENT OF ECUADORIAN CUTANEOUS LEISHMANIASIS. American Journal of Tropical Medicine and Hygiene 1991;45(1):92-7.

2. Herrick AL, Hollis S, Schofield D, Rieley F, Blann A, Griffin K, et al. A double-blind placebo-controlled trial of antioxidant therapy in limited cutaneous systemic sclerosis. Clin Exp Rheumatol 2000;18(3):349-56.

Results could not be translated

1. Momeni A, Shahidi S, Seirafian S, Taheri S, Kheiri S. Effect of Allopurinol in Decreasing Proteinuria in Type 2 Diabetic Patients. Iranian Journal of Kidney Diseases 2010;4(2):128-32.

Follow-up or treatment duration <28 days

1. Avidan MS, Meehan N, Sherwood RA, Pj. Allopurinol for the prevention of reperfusion injury during open heart surgery [abstract]. British journal of anaesthesia; 1999:176p.

2. Davis PS, Deller DJ. Effect of a xanthine-oxidase inhibitor (allopurinol) on radioiron absorption in man. Lancet; 1966:470-2.

3. DeConti RC, Calabresi P. Use of allopurinol for prevention and control of hyperuricemia in patients with neoplastic disease. N Engl J Med 1966;274(9):481-6.

4. Johnson WD, Kayser KL, Brenowitz JB, Saedi SF. A RANDOMIZED CONTROLLED TRIAL OF ALLOPURINOL IN CORONARY-BYPASS SURGERY. American Heart Journal 1991;121(1):20-4.

5. Nazari A, Sadrolhefazi B, Nikoofar A, Erfan M, Azizian H, Alamy M. Allopurinol mouthwash for prevention or alleviation radiotherapy induced oral mucositis: a randomized, placebo-controlled trial. Daru-Journal of Faculty of Pharmacy 2007;15(4):227-30.

6. Nct. Phase 2 Study to Determine if Allopurinol Blocks Features of Metabolic Syndrome Induced by Fructose Ingestion. ClinicalTrials.gov [http://clinicaltrials.gov]; 2008.

7. Nicotero JA, Scheib ET, Martinez R, Rodnan GP, Shapiro AP. Prevention of hyperuricemia by allopurinol in hypertensive patients treated with chlorothiazide. N Engl J Med 1970;282(3):133-5.

8. Parmley LF, Mufti AG, Downey JM. ALLOPURINOL THERAPY OF ISCHEMIC-HEART-DISEASE WITH INFARCT EXTENSION. Canadian Journal of Cardiology 1992;8(3):280-6.

9. Scott JT. Comparison of allopurinol and probenecid. Annals of the rheumatic diseases; 1966:623-6.

10. Subramanian S, Abraham OC, Usha S. Allopurinol as an adjust to quinine in the treatment of uncomplicated falciparum malaria [abstract no:83]. Journal of the Association of Physicians of India; 2001.

11. Tsavaris N, Bacoyannis C, Milonakis N, Sarafidou M, Zamanis N, Magoulas D, et al. Folinic acid plus high-dose 5-fluorouracil with allopurinol protection in the treatment of advanced colorectal carcinoma. Eur J Cancer 1990;26(10):1054-6.

12. Vriens MR, Marinelli A, Harinck HIJ, Zwinderman KH, Van de Velde CJH, Author A, et al. The role of allopurinol in human liver ischemia/reperfusion injury: A prospective randomized clinical trial. Hepato-Gastroenterology 2002;49(46):1069-73.

13. Wortmann RL, MacDonald PA, Hunt B, Jackson RL. Effect of Prophylaxis on Gout Flares After the Initiation of Urate-Lowering Therapy: Analysis of Data From Three Phase III Trials. Clinical Therapeutics 2010;32(14):2386-97.

14. Ziaee AM, Akhavizadegan H, Karbakhsh M. Effect of allopurinol in chronic nonbacterial prostatitis: a double blind randomized clinical trial. Int Braz J Urol;32(2):181-6.

Abstracts or full-text articles with no extractable data

1. Andreou I, Tousoulis D, Papadimitriou CA, Tentolouris C, Tsiatas M, Siasos G, et al. Effects of rosuvastatin and allopurinol treatment on endothelial progenitor cells in patients with chronic heart failure. European Heart Journal 2010;31 SUPPL. 1:853.

2. Apt W, Aguilera X, Arribada A, Perez C, Miranda C, Sanchez G, et al. Treatment of chronic Chagas' disease with itraconazole and allopurinol. American Journal of Tropical Medicine and Hygiene 1998;59(1):133-8.

3. Bergamini C, Cicoira M, Rossi A, Cassater D, Zanolla L, Vassanelli C, et al. Allopurinol lowers NT-proBNP plasma levels and improves diastolic function in heart failure patients: A double-blind, placebo-controlled study. European Heart Journal 2010;31 SUPPL. 1:849.

4. Berthoux FC, Juge J, Genin C, Sabatier JC, Assenat H. DOUBLE-BLIND TRIAL OF ALLOPURINOL AS A PREVENTIVE TREATMENT IN CALCIUM UROLITHIASIS. Mineral and Electrolyte Metabolism 1979;2(4-5):207-.

5. Burshtein S, Weiser M, Gershon AA, Marian G, Vlad N, Grecu IG, et al. Allopurinol for mania. A randomized trial of allopurinol versus placebo as add-on treatment in manic bipolar patients. European Neuropsychopharmacology 2013;23 SUPPL. 2:S378.

6. Butler R, Morris AD, Belch JJ, Hill A, Struthers AD. Allopurinol normalizes endothelial dysfunction in type 2 diabetics with mild hypertension. Hypertension 2000;35(3):746-51.

7. Farquharson CA, Butler R, Hill A, Belch JJ, Struthers AD. Allopurinol improves endothelial dysfunction in chronic heart failure. Circulation 2002;106(2):221-6.

8. Green D, O'Driscoll G, Rankin J, Taylor R. Endothelium-derived nitric oxide activity in forearm vessels of hypercholesterolaemic patients is unaffected by allopurinol. [abstract]. Australian and New Zealand journal of medicine; 1998:105.

9. Ivanov DD, Ivanova MD, Author A, Nephrology, Rrt Dept NMAo, Postgraduate Education named P.Shupik KU, et al. Febuxostat improves GFR and BP in non-diabetic adults with CKD 2-3. Nephrology Dialysis Transplantation 2013;28 SUPPL. 1:i48.

10. Kamatani N, Fujimori S, Hada T, Hosoya T, Kato R, Matsuzawa Y, et al. Phase II dose-response clinical trial using febuxostat (TMX-67), a novel-type xanthine Oxidase/Xanthine dehydrogenase inhibitor, for gout and hyperuricemia. Arthritis and Rheumatism 2003;48(9):S530-S.

11. Kamatani N, Fujimori S, Hada T, Hosoya T, Matsuzawa Y, Ueda J, et al. Febuxostat, a novel non-purine selective inhibitor of xanthine oxidase, in a Phase III placebo-controlled double-blind clinical trial in Japanese subjects with gout or hyperuricemia. Arthritis and Rheumatism 2004;50(9):S337-S.

12. Mendell JR, Wiechers DO. Lack of benefit of allopurinol in Duchenne dystrophy. Muscle Nerve 1979;2(1):53-6.

13. Miano L, Petta S, Paradisogalatioto G, Goldoni S. PLACEBO-CONTROLLED DOUBLE-BLIND-STUDY OF ALLOPURINOL IN SEVERE RECURRENT IDIOPATHIC RENAL LITHIASIS - PRELIMINARY-RESULTS. Urological Research 1984;12(1):46-.

14. Neu I, Mueller J, Authenrieth W, Hahn F. Treatment of hyperuricemia with special reference to tolerability. Double-blind crossover study of micronized benzbromaron vs. allopurinol. <ORIGINAL> THERAPIE DER HYPERURIKAMIE UNTER BESONDERER BERUCKSICHTIGUNG DER VERTRAGLICHKEIT. Die Therapiewoche; 1981:3168-75.

15. Persson BE, Ronquist G. Abacterial prostatitis and uric acid in prostatic expressate, and objective and subjective effect of allopurinol treatment: A double- blind, placebo-controlled study. Scandinavian journal of urology and nephrology. Supplementum; 1994:21-2.

16. Persson BE, Ronquist G, Author A, Department of Urology UH, Uppsala S, Correspondence A, et al. Abacterial prostatitis and uric acid in prostatic expressate, and objective and subjective effect of allopurinol treatment: A double-blind, placebo-controlled study. Scandinavian Journal of Urology and Nephrology, Supplement 1994(164):21-2.

17. Saag KG, Becker MA, Whelton A, MacDonald PA, Zhou Y, Gunawardhana L. Effect Of Febuxostat On Serum Urate Levels In Gout Subjects With Hyperuricemia and Moderate-To-Severe Renal Impairment: A Randomized Controlled Trial. Arthritis and Rheumatism 2013;65:S498-S9.

18. Saag KG, Becker MA, Whelton A, MacDonald PA, Zhou Y, Gunawardhana L, et al. Effect of febuxostat on serum urate levels in gout subjects with hyperuricemia and moderate-to-severe renal impairment: A randomized controlled trial. Arthritis and Rheumatism 2013;65 SUPPL. 10:S498-S9.

19. Sharifipour E, Taheraghdam A, Farhoudi M, Pashapour A, Sadeghi Hokmabadi E, Mostafaie S, et al. Effect of allopurinol in ischemic stroke prognosis. Cerebrovascular Diseases 2011;31 SUPPL. 2:129.

20. Smith MJ, Boyce WH. Allopurinol and urolithiasis. J Urol 1969;102(6):750-3.

21. Streiker FB. The use of allopurinol in controlling hyperuricemia. J Am Podiatry Assoc 1969;59(3):87-9.

22. Velez I, Agudelo S, Hendrickx E, Puerta J, Grogl M, Modabber F, et al. Inefficacy of allopurinol as monotherapy for Colombian cutaneous leishmaniasis: A randomized, controlled trial. Annals of Internal Medicine 1997;126(3):232-6.

23. Walter-Sack I, De Vries JX, Ernst B, Frei M, Kolb S, Kosmowski J, et al. Uric acid lowering effect of oxipurinol sodium in hyperuricemic patients: Therapeutic equivalence to allopurinol. Journal of Rheumatology 1996;23(3):498-501.

24. Wang XZ, Song YX, Song QF, Zhang LN, Jia HJ. Effect of allopurinol on the function of blood vessel endothelium in patients with essential hypertension complicating hyperuricemia. Journal of china pharmacy; 2005.

25. Williams R, Author A. Slowing the progression of renal disease with allopurinol. Nature Clinical Practice Nephrology 2006;2(4):180.

26. Yamanaka H, Nishioka K, Suzuki T, Kohno K. Allopurinol metabolism in a patient with xanthine oxidase deficiency. Ann Rheum Dis 1983;42(6):684-6.

Review, editorial, observational or quasi-experimental studies

1. Allopurinol and the control of hyperuricemia in neoplastic disease. Med Lett Drugs Ther 1968;10(25):103-4.

2. Bayram D, Tugrul Sezer M, Inal S, Altuntas A, Kidir V, Orhan H. The effects of allopurinol on metabolic acidosis and endothelial functions in chronic kidney disease patients. Clin Exp Nephrol 2014.

3. Beutler AM, Rull M, Schlesinger N, Baker DG, Hoffman BI, Schumacher BI, Jr., et al. Treatment with allopurinol decreases the number of acute gout attacks despite persistently elevated serum uric acid levels [1]. Clinical and Experimental Rheumatology 2001;19(5):595.

4. Blanco Vicente E. Effect of high doses of allopurinol patients with chronic stable angina: randomized controlled placebo. Revista Clinica Espanola 2011;211(4):205-.

5. Brown CH, 3rd, Stashick E, Carbone PP. Clinical efficacy and lack of toxicity of allopurinol (NSC-1390) given intravenously. Cancer Chemother Rep 1970;54(2):125-9.

6. Castelli P, Munari M, Author A, Anestesia e Rianimazione MI, Correspondence A, P. Castelli AeRMI. Improvement of cardiac function by allopurinol [2]. Journal of Cardiothoracic and Vascular Anesthesia 1997;11(6):809-10.

7. Chalmers RA, Kromer H, Scott JT, Watts RWE. A COMPARATIVE STUDY OF XANTHINE OXIDASE INHIBITORS ALLOPURINOL AND OXIPURINOL IN MAN. Clinical Science 1968;35(2):353-&.

8. Chohan S, Becker M, MacDonald P, Palmer R, Chefo S, Jackson R, et al. Efficacy and safety of febuxostat and allopurinol in women with gout, an older subset with increased comorbidity. Journal of Rheumatology 2011;38(6):1152.

9. DeConti RC, Calabresi P. Use of allopurinol for control of hyperuricemia in leukemia and lymphoma. Conn Med 1967;30(6):430-6.

10. Drew CD. The importance of renal damage due to hyperuricaemia and its prevention by allopurinol. J Med Liban 1970;23(2):161-72.

11. el-Gamel A, Evans C, Keevil B, Aziz T, Rahman A, Campbell C, et al. Effect of allopurinol on the metabolism of azathioprine in heart transplant patients. Transplant Proc 1998;30(4):1127-9.

12. Ettinger B, Citron J, Tang A. CONTROLLED-STUDIES IN STONE PROPHYLAXIS - COMPARISON OF PLACEBO VERSUS ALLOPURINOL CHLORTHALIDONE MAGNESIUM-HYDROXIDE. Urological Research 1984;12(1):49-.

13. Frang D, Berenyi M, Vegh A. [Allopurinol treatment of patients with uric acid calculi]. Acta Chir Acad Sci Hung 1973;14(2):105-11.

14. Gavin AD, Struthers AD. Allopurinol reduces B-type natriuretic peptide concentrations and haemoglobin but does not alter exercise capacity in chronic heart failure. Heart 2005;91(6):749-53.

15. Gunther R, Knapp E. [Effect of allopurinol (Zyloric) on uric acid, creatinine, fasting blood sugar and plasma lipids in patients with gout]. [German]. Wiener klinische Wochenschrift; 1970:78-82.

16. Jacobs F, Mamzer-Bruneel MF, Skhiri H, Thervet E, Legendre C, Kreis H. Safety of the mycophenolate mofetil-allopurinol combination in kidney transplant recipients with gout. Transplantation 1997;64(7):1087-8.

17. Lee M, Chen Y, Shen C, Author A, Institute of Biomedical Sciences A, Sinica TT, et al. A prospective study of HLA-Bnull5801 genotyping for the prevention of allopurinol

induced severe cutaneous adverse reactions. Clinical Pharmacology and Therapeutics 2012;91 SUPPL. 1:S107.

18. Marangella M, Tricerri A, Sonego S, Ronzani M, Fruttero B, Daniele PG, et al. Critical evaluation of various forms of therapy for idiopathic calcium stone disease. Proc Eur Dial Transplant Assoc 1983;20:434-9.

19. McNaughton Collins M, Wilt T. Allopurinol for chronic prostatitis. Cochrane Database Syst Rev 2000(2):Cd001041.

20. Meltzer M, Author A, Thomas Jefferson University PPAUS, Correspondence A, M. Meltzer TJUPPAUS. Allopurinol versus rilonacept for the prevention of gout flares: Comment on the Article by Schumacher et al. Arthritis Care and Research 2013;65(5):838.

21. Muszkowska-Penson J, Chodorowski Z. [Allopurinol in the treatment of gout]. Pol Tyg Lek 1969;24(8):286-8.

22. Nickel JC, Siemens DR, Lundie MJ. Ameliorative effect of allopurinol on nonbacterial prostatitis: A parallel double-blind controlled study. Journal of Urology 1997;157(2):628-.

23. Pai BH, Swarnalatha G, Ram R, Dakshinamurty KV. Allopurinol for prevention of progression of kidney disease with hyperuricemia. Indian J Nephrol 2013;23(4):280-6.

24. Perez-Ruiz F, Alonso-Ruiz A, Calabozo M, Herrero-Beites A, Garcia-Erauskin G, Ruiz-Lucea E, et al. Efficacy of allopurinol and benzbromarone for the control of hyperuricaemia. A pathogenic approach to the treatment of primary chronic gout. Annals of the Rheumatic Diseases 1998;57(9):545-9.

25. Pochedly C. Hyperuricemia in leukemia and lymphoma. Postgrad Med 1974;55(6):93-9.

26. Pohar S, Murphy G. Febuxostat for prevention of gout attacks. Issues Emerg Health Technol 2006(87):1-4.

27. Radak-Perovic M, Zlatkovic-Svenda M. The Efficacy and Tolerability of Allopurinol Dose Escalation in Patients with Gout. Srpski Arhiv Za Celokupno Lekarstvo 2013;141(5-6):333-6.

28. Reinders MK, Haagsma C, Jansen TL, Roon EN, Delsing J, Laar MA, et al. A randomised controlled trial on the efficacy and tolerability with dose escalation of allopurinol 300-600 mg/day versus benzbromarone 100-200 mg/day in patients with gout. Annals of the rheumatic diseases; 2009:892-7.

29. Salim AS. Oxygen-derived free-radical scavengers prolong survival in gastric cancer. Chemotherapy; 1992:135-44.

30. Salim AS. The relationship between Helicobacter pylori and oxygen-derived free radicals in the mechanism of duodenal ulceration. Internal medicine (Tokyo, Japan); 1993:359-64.

31. Salim AS. Scavengers of oxygen-derived free radicals prolong survival in advanced colonic cancer. A new approach. Tumour biology; 1993:9-17.

32. Schumacher HR, Sundy JS, Terkeltaub R, Knapp HR, Mellis S, Soo Y, et al. Placebo-controlled study of rilonacept for gout flare prophylaxis during initiation of urate-lowering therapy. Arthritis and Rheumatism 2009;60 SUPPL. 10:1096.

33. Scott R, Paterson PJ, Mathieson A, Smith M. The effect of allopurinol on urinary oxalate excretion in stone formers. Br J Urol 1978;50(7):455-8.

34. Shi Y, Chen W, Jalal D, Li Z, Chen W, Mao H, et al. Clinical Outcome of Hyperuricemia in IgA Nephropathy: A Retrospective Cohort Study and Randomized Controlled Trial. Kidney & Blood Pressure Research 2012;35(3):153-60.

35. Singh JA. Can racial disparities in optimal gout treatment be reduced? Evidence from a randomized trial. Bmc Medicine 2012;10.

36. Smith MJ. Allopurinol and calcium-stone formers. Lancet; 1973:315.

37. Smith MJ. Placebo versus allopurinol for renal calculi. Journal of urology; 1977:690-2.

38. Smith MJ. Placebo versus allopurinol for recurrent urinary calculi. Proceedings of the European Dialysis and Transplant Association. European Dialysis and Transplant Association; 1983:422-6.

39. Sramek F. [Use of allopurinol in the treatment of urolithiasis]. Rozhl Chir 1975;54(7):472-6.

40. Stamp L, O'Donnell JL, Zhang M, Frampton C, Barclay M, Chapman PT, et al. Increasing allopurinol dose above the recommended range is effective and safe in chronic gout, including in those with renal impairment a pilot study. Arthritis and Rheumatism 2009;60 SUPPL. 10:1950.

41. Stamp LK, O'Donnell JL, Zhang M, James J, Frampton C, Barclay ML, et al. Using allopurinol above the dose based on creatinine clearance is effective and safe in patients

with chronic gout, including those with renal impairment. Arthritis Rheum 2011;63(2):412-21.

42. Stolbach L, Begg C, Bennett JM, Silverstein M, Falkson G, Harris DT, et al. Evaluation of bone marrow toxic reaction in patients treated with allopurinol. Jama 1982;247(3):334-6.

43. Stone PH, Author A, Cardiovascular Division Ba, Women's Hospital HMSFSBMA, States U, Correspondence A, et al. Allopurinol: A new anti-ischemic role for an old drug. Journal of the American College of Cardiology 2011;58(8):829-30.

44. Sziegoleit W, Böger W, Noack W, Förster W. Therapeutic trial with Allopurinol in peripheral arterial occlusion in comparison to Xanthinolnikotinat. Das Deutsche Gesundheitswesen; 1981:171-4.

45. Tausche AK, Christoph M, Forkmann M, Richter U, Kopprasch S, Bielitz C, et al. As compared to allopurinol, urate-lowering therapy with febuxostat has superior effects on oxidative stress and pulse wave velocity in patients with severe chronic tophaceous gout. Rheumatology International 2014;34(1):101-9.

46. Teramoto S, Kume H, Yamaguchi Y, Yamamoto H, Hanaoka Y, Ishii M, et al. Improvemnt of endothelial function with allopurinol may oocur in selected patients with OSA: Effect of age and sex [3]. European Respiratory Journal 2007;29(1):216-7.

47. Terawaki H, Nakayama M, Miyazawa E, Murata Y, Nakayama K, Matsushima M, et al. Effect of allopurinol on cardiovascular incidence among hypertensive nephropathy patients: the Gonryo study. Clinical and Experimental Nephrology 2013;17(4):549-53.

48. Thomson WH, Smith I. X-linked recessive (Duchenne) muscular dystrophy (DMD) and purine metabolism: effects of oral allopurinol and adenylate. Metabolism: clinical and experimental; 1978:151-63.

49. Tiselius HG, Larsson L. Urine composition in patients with renal stone disease during treatment with allopurinol. Scand J Urol Nephrol 1980;14(1):65-71.

50. Toledopereyra LH, Simmons RL, Olson LC, Najarian JS. CLINICAL EFFECT OF ALLOPURINOL ON PRESERVED KIDNEYS - RANDOMIZED DOUBLE-BLIND-STUDY. Annals of Surgery 1977;185(1):128-31.

51. Tsuruta Y, Nitta K, Akizawa T, Fukuhara S, Saito A, Kurokawa K, et al. Allopurinol and all-cause mortality among hemodialysis (HD) patients (pts): Results from the dialysis outcomes and practice patterns study (DOPPS) in Japan. NDT Plus 2010;3 SUPPL. 3:iii92.

52. Tuta L, Stanigut A, Author A, Ovidius University of Constanta Fo, Medicine CR, Correspondence A, et al. Allopurinol therapy for hyperuricemia reduces inflammation and progression of renal disease in moderate chronic kidney disease. Nephrology Dialysis Transplantation 2014;29 SUPPL. 3:iii118.

53. von Haehling S, Bode-Boger SM, Martens-Lobenhoffer J, Rauchhaus M, Schefold JC, Genth-Zotz S, et al. Elevated levels of asymmetric dimethylarginine in chronic heart failure: a pathophysiologic link between oxygen radical load and impaired vasodilator capacity and the therapeutic effect of allopurinol. Clin Pharmacol Ther 2010;88(4):506-12.

54. Walters M. New therapeutic targets in stroke prevention: the effects of allopurinol on the cerebrovasculature of patients with subcortical stroke. Current Controlled Trials (http://www.controlled-trials.com/); 2005.

55. Walters M, Dawson J. Allo IMT study. The effect of allopurinol on carotid ultrasound intima-thickness and markers of endothelial function in patients with recent stroke. UK Clinical Research Network Portfolio Database (http://public.ukcrn.org.uk/search/); 2008.

56. Whelton A, MacDonald PA, Chefo S, Gunawardhana L. Preservation of Renal Function During Gout Treatment With Febuxostat: A Quantitative Study. Postgraduate Medicine 2013;125(1):106-14.

57. Whelton A, MacDonald PA, Zhao L, Hunt B, Gunawardhana L. Renal Function in Gout Long-Term Treatment Effects of Febuxostat. Jcr-Journal of Clinical Rheumatology 2011;17(1):7-13.

58. White WB, Chohan S, Dabholkar A, Hunt B, Jackson R. CArdiovascular Safety of Febuxostat and Allopurinol in Patients With Gout and Cardiovascular ComoRbiditiES. American Heart Journal 2012;164(1):14-20.

59. Wortmann RL, Becker MA, Schumacher HR, Jr., MacDonald PA, Hunt BJ, Joseph-Ridge N. Effect of febuxostat or allopurinol on the clinical manifestations of gout: Reduction in gout flares and tophus size over time in the EXCEL trial. Arthritis and Rheumatism 2006;54(9):S642-S.

60. Wright DF, Stamp LK, Merriman TR, Barclay ML, Duffull SB, Holford NH. The population pharmacokinetics of allopurinol and oxypurinol in patients with gout. Eur J Clin Pharmacol 2013;69(7):1411-21.

61. Wu AH, Ghali JK, Neuberg GW, O'Connor CM, Carson PE, Levy WC. Uric acid level and allopurinol use as risk markers of mortality and morbidity in systolic heart failure. American Heart Journal 2010;160(5):928-33.

62. Yelken B, Gorgulu N, Altun I, Yilmaz A, Caliskan YK, Yazici H, et al. Reduction of uric acid levels with allopurinol treatment improves endothelial function in the patients with chronic renal failure. NDT Plus 2010;3 SUPPL. 3:iii177-iii8.

63. Yiginer O, Ozcelik F, Aparci M, Isilak Z, Uz O, Author A, et al. The beneficial effects of allopurinol in cardiology practice: Decrease in uric acid and vascular oxidative stress/ the effects of lowering uric acid levels using allopurinol on markers of metabolic syndrome in end-stage renal disease patients: A pilot study ORIGINAL (NON-ENGLISH) TITLE Allopurinolun kardiyoloji pratigindeki faydali etkileri: Urik asitte ve vaskuler oksidatif streste azalma/ Urik asit seviyelerinin allopurinol ile azaltilmasinin son donem bobrek hastalarindaki metabolik sendrom belirleyicilerine etkisi: Pilot calisma. Anadolu Kardiyoloji Dergisi 2010;10(3):294-5.

64. Yu TF. The effect of allopurinol in primary and secondary gout. Arthritis Rheum 1965;8(5):905-6.

65. Zhang J, Liu XY, Du XL, Author A, Correspondence A, J. Zhang Ejsc. Febuxostat - A novel drug for the treatment of gout. Chinese Pharmaceutical Journal 2010;45(15):1197-8.

66. Zöllner N, Schattenkirchner M. [Allopurinol in the treatment of gout and uric acid nephrolithiasis]. Deutsche medizinische Wochenschrift (1946); 1967:654-60.

Repeated records

1. Apt Baruch W, Aguilera Martín X, Arribada C A, Pérez Baladrón C, Miranda Sallorenzo C, Zulantay Alfaro I, et al. Tratamiento de la enfermedad de Chagas humana crónica con itraconazol y alopurinol: informe preliminar. Treatment of chronic human Chagas disease with itraconazole and allopurinol: preliminary report. Rev Med Chil;122(4):420-7.

2. Apt W, Aguilera X, Arribada A, Perez C, Miranda C, Sanchez G, et al. Treatment of chronic Chagas' disease with itraconazole and allopurinol. American Journal of Tropical Medicine and Hygiene 1998;59(1):133-8.

3. Baggio B, Gambaro G, Paleari C, Author A, Universita di Padova P, Correspondence A, et al. Hydrochlorothiazide and allopurinol vs. placebo on urinary excretion of stone promoters and inhibitors. Current Therapeutic Research - Clinical and Experimental 1983;34(1 I):145-51.

4. Banks PA, Hughes M, Ferrante M, Noordhoek EC, Ramagopal V, Slivka A. Does allopurinol reduce pain of chronic pancreatitis? International Journal of Pancreatology 1997;22(3):171-6.

5. Banks PA, Hughes M, Ferrante M, Noordhoek EC, Ramagopal V, Slivka A, et al. Does allopurinol reduce pain of chronic pancreatitis. International Journal of Pancreatology 1997;22(3):171-6.

6. Becker MA, Schumacher HR, Jr., Wortmann RL, MacDonald PA, Palo WA, Eustace D, et al. Febuxostat, a Novel Nonpurine Selective Inhibitor of Xanthine Oxidase. Arthritis and Rheumatism 2005;52(3):916-23.

7. Berthoux FC, Juge J, Ducret F. DOUBLE-BLIND TRIAL OF ALLOPURINOL AS A PREVENTIVE TREATMENT IN RECURRENT CALCIUM NEPHROLITHIASIS. Mineral and Electrolyte Metabolism 1981;6(4-5):232-.

8. Butler R, Morris AD, Struthers AD. Allopurinol improves endothelial function in patients with non-insulin dependent diabetes mellitus [abstract]. Scottish medical journal; 1998:61.

9. Dawson J, Higgins P, Quinn T, Harrow C, Lees K, Walters M, et al. Effect of allopurinol upon cerebrovascular reactivity following subcortical stroke. Basic and Clinical Pharmacology and Toxicology 2009;105 SUPPL. 1:33.

10. Gianella A, Holzman A, Lihoshi N. Efficacy of Allopurinol in chronic Chagas disease. Results of a randomized controlled trial in Santa Cruz, Bolivia. Bol Cient CENETROP; 1997:25-9.

11. Goldfarb DS, MacDonald PA, Hunt B, Gunawardhana L. Febuxostat in Gout: Serum Urate Response in Uric Acid Overproducers and Underexcretors. Journal of Rheumatology 2011;38(7):1385-9.

12. Harrow C, Muir S, Lees K, Walters M. Safety, tolerability and pharmacodynamics of Allopurinol in acute stroke patients: a pilot study. Cerebrovascular diseases (Basel, Switzerland); 2005:74.

13. Horsfall PA, Plummer J, Allan WG, Girling DJ, Nunn AJ, Fox W. Double blind controlled comparison of aspirin, allopurinol and placebo in the management of arthralgia during pyrazinamide administration. Tubercle 1979;60(1):13-24.

14. Jahangard L, Soroush S, Haghighi M, Ghaleiha A, Bajoghli H, Holsboer-Trachsler E, et al. In a double-blind, randomized and placebo-controlled trial, adjuvant allopurinol improved symptoms of mania in in-patients suffering from bipolar disorder. European Neuropsychopharmacology 2014;24(8):1210-21.

15. Joelsson M, Andersson M, Bark T, Gullberg K, Hallgren T, Jiborn H, et al. Allopurinol as prophylaxis against pouchitis following heal pouch-anal anastomosis for ulcerative colitis - A randomized placebo-controlled double-blind study. Scandinavian Journal of Gastroenterology 2001;36(11):1179-84.

16. Kanbay M, Huddam B, Azak A, Solak Y, Kadioglu GK, Kirbas I, et al. A Randomized Study of Allopurinol on Endothelial Function and Estimated Glomular Filtration Rate in Asymptomatic Hyperuricemic Subjects with Normal Renal Function. Clinical Journal of the American Society of Nephrology 2011;6(8):1887-94.

17. Kao MP, Ang DS, Struthers AD, Author A, Division of Medical S, University of Dundee UK, et al. Allopurinol improves vascular function in patients with chronic kidney disease. NDT Plus 2010;3 SUPPL. 3:iii3.

18. Machado-Vieira R, Kapczinski F, Lara DR, Souza DO, Soares JC, Filho GM. Superior antimanic efficacy of allopurinol compared to dipyridamol and placebo as add-on treatment to lithium in unmedicated bipolar patients during a mania episode: a double-blind placebo-controlled trial. Bipolar Disorders 2007;9:69-70.

19. Machado-Vieira R, Kapczinski F, Souza DO, Lara DR, Soares JC. A double-blind controlled study of adjunctive allopurinol or dipyridamole to lithium in the treatment of bipolar mania: preliminary results. Bipolar Disorders 2005;7:73-.

20. Meirovich CI, Montrull HL, Gallerano RH, Sosa RR. [Allopurinol in the treatment of chronic Chagas' disease]. Arq Bras Cardiol 1985;45(3):217-23.

21. Muir SW, Harrow C, Dawson J, Lees KR, Weir CJ, Sattar N, et al. Allopurinol Use Yields Potentially Beneficial Effects on Inflammatory Indices in Those With Recent Ischemic Stroke A Randomized, Double-Blind, Placebo-Controlled Trial. Stroke 2008;39(12):3303-7.

22. Nakamura N. [Efficacy of Benzbromarone for Hyperuricemia (Part 2): Comparison with Allopurinol]. Rinsho to Kenkyu (The Japanese Journal of Clinical and Experimental Medicine); 1972:1381-4.

23. Naoyuki K, Shin F, Toshikazu H, Tatsuo H, Kenjiro K, Toshitaka N, et al. Placebo-Controlled, Double-Blind Study of the Non-Purine-Selective Xanthine Oxidase Inhibitor Febuxostat (TMX-67) in Patients With Hyperuricemia Including Those With Gout in Japan. Jcr-Journal of Clinical Rheumatology 2011;17(4):S19-S26.

24. Naoyuki K, Shin F, Toshikazu H, Tatsuo H, Kenjiro K, Toshitaka N, et al. An Allopurinol-Controlled, Multicenter, Randomized, Open-Label, Parallel Between-Group, Comparative Study of Febuxostat (TMX-67), a Non-Purine-Selective Inhibitor of Xanthine

Oxidase, in Patients With Hyperuricemia Including Those With Gout in Japan. Jcr-Journal of Clinical Rheumatology 2011;17(4):S44-S9.

25. Nct. Treatment of Uric Acid With the Xanthine Oxidase Inhibitor Febuxostat: Effects on Blood Pressure, Metabolic Markers, and Aortic Stiffness in Prehypertensive. ClinicalTrials.gov [http://clinicaltrials.gov]; 2011.

26. Nct. Febuxostat, Blood Pressure and the Intrarenal Renin-Angiotensin System (RAS). ClinicalTrials.gov [http://clinicaltrials.gov]; 2011.

27. Nct. A Phase 2, Double-Blind, Placebo-Controlled Study to Assess the Effect of Febuxostat 80 mg Once Daily Compared to Placebo on Ambulatory Blood Pressure in Subjects With Hyperuricemia and Hypertension. ClinicalTrials.gov [http://clinicaltrials.gov]; 2012.

28. Noman A, Ang DSC, Ogston S, Lang CC, Struthers AD. Effect of high-dose allopurinol on exercise in patients with chronic stable angina: a randomised, placebo controlled crossover trial. Lancet 2010;375(9732):2161-7.

29. Noman A, Rekhraj S, Lang CC, Struthers AD, Author A, University of D, et al. The effect of high dose allopurinol on the augmentation index in patients with coronary artery disease. European Heart Journal 2011;32 SUPPL. 1:574.

30. O'Driscoll JG, Green DJ, Rankin JM, Taylor RR, Author A, Department of Med a, et al. Nitric oxide-dependent endothelial function is unaffected by allopurinol in hypercholesterolaemic subjects. Clinical and Experimental Pharmacology and Physiology 1999;26(10):779-83.

31. O'Duffy JD, Scherbel AL. Treatment of gout and urate calculi with allopurinol. Cleve Clin Q 1968;35(3):145-54.

32. PerezRuiz F, AlonsoRuiz A, Calabozo M, RuizLucea E, Aretxabala I, Fraiz E. Comparative study of the efficacy of benzbromarone and allopurinol for the therapy of hyperuricemia in chronic primary gout. Arthritis and Rheumatism 1996;39(9):355-.

33. Perez-Ruiz F, Sundy J, Krishnan E, Hingorani V, Welp J, Rodgers T, et al. EFFICACY AND SAFETY OF LESINURAD (RDEA594), A NOVEL URAT1 INHIBITOR, IN COMBINATION WITH ALLOPURINOL IN ALLOPURINOL-REFRACTORY GOUT PATIENTS: RESULTS FROM A RANDOMIZED, BLINDED, PLACEBO-CONTROLLED, PHASE 2B EXTENSION STUDY. Annals of the Rheumatic Diseases 2012;71:439-.

34. Persson BE, Ronquist G, Ekblom M. Ameliorative effect of allopurinol on nonbacterial prostatitis: A parallel double-blind controlled study. Journal of Urology 1996;155(3):961-4.

35. Poor G, Josfay J, Judak A, Ludanyi E, Mituszova M. [The effect of allopurinol therapy on kidney processes concomitant with gout]. Orv Hetil 1987;128(37):1939-43.

36. Puntoni M, Branchi D, Argusti A, Zanardi S, Crosta C, Meroni E, et al. Randomized, presurgical study of allopurinol vs. placebo in subjects with colorectal adenomas. Cancer Prevention Research 2010;3:12 SUPPL. 2.

37. Rajendra NS, Ireland S, George J, Belch JJ, Lang CC, Struthers AD. Mechanistic insights into the therapeutic use of high-dose allopurinol in angina pectoris. J Am Coll Cardiol 2011;58(8):820-8.

38. Rashid MA, William-Olsson G. Influence of allopurinol on cardiac complications in open heart operations. Ann Thorac Surg 1991;52(1):127-30.

39. Reinders MK, Haagsma C, Jansen TLTA, Van Roon EN, Delsing J, Van De Laar MAFJ, et al. A randomised controlled trial on the efficacy and tolerability with dose escalation of allopurinol 300-600 mg/day versus benzbromarone 100-200 mg/day in patients with gout. Annals of the Rheumatic Diseases 2009;68(6):892-7.

40. Reinders MK, Roon EN, Jansen TL, Delsing J, Griep EN, Hoekstra M, et al. Efficacy and tolerability of urate-lowering drugs in gout: a randomised controlled trial of benzbromarone versus probenecid after failure of allopurinol. Annals of the rheumatic diseases; 2009:51-6.

41. Salim AS. Scavenging free radicals to prevent stress-induced gastric mucosal injury. Lancet; 1989:1390.

42. Salim AS. Oxygen-derived free radicals and the prevention of duodenal ulcer relapse: a new approach. American journal of the medical sciences; 1990:1-6; discussion 7-8.

43. Schumacher HR, Becker MA, Wortmann RL, MacDonald PA, Hunt B, Streit J, et al. Febuxostat vs allopurinol and placebo in subjects with hyperuricemia and gout: The 28-week APEX study. Arthritis and Rheumatism 2005;52(9):S680-S.

44. Scott JT, Hall AP, Grahame R. Allopurinol in treatment of gout. British medical journal; 1966:321-7.

45. Scott R, Lewi H. Therapeutic management of upper urinary tract stone disease in 172 subjects. Urology; 1989:277-81.

46. Sezai A, Soma M, Nakata K-i, Hata M, Yoshitake I, Wakui S, et al. Comparison of Febuxostat and Allopurinol for Hyperuricemia in Cardiac Surgery Patients (NU-FLASH Trial). Circulation Journal 2013;77(8):2043-9.

47. Shehab AM, Butler R, MacFadyen RJ, Struthers AD. A placebo-controlled study examining the effect of allopurinol on heart rate variability and dysrhythmia counts in chronic heart failure. Br J Clin Pharmacol 2001;51(4):329-34.

48. Siu YP, Leung KT, Tong MKH, Kwan TH. Use of allopurinol in slowing the progression of renal disease through its ability to lower serum uric acid level. American Journal of Kidney Diseases 2006;47(1):51-9.

49. Smith V. PLACEBO VERSUS ALLOPURINOL FOR RECURRENT URINARY CALCULI. Kidney International 1983;24(3):419-20.

50. Stern LM, Fewings JD, Bretag AH, Author A, Regency Park Cent P, Handicapped Child A, et al. The progression of Duchenne muscular dystrophy: Clinical trial of allopurinol therapy. Neurology 1981;31(4 I):422-6.

51. Taheraghdam AA. The effect of allopurinol on ischemic stroke functional prognosis. 2011.

52. Templeton JS. Azapropazone or allopurinol in the treatment of chronic gout and/or hyperuricaemia. A preliminary report. British journal of clinical practice; 1982:353-8.

53. Togha M, Ahmadi B, Akhondzadeh S, Razeghi S. Allopurinol in refractory epilepsy: A double blind study. Tehran University Medical Journal; 2008:23-7.

54. Togha M, Ahmadi B, Akhondzadeh S, Razeghi S, Author A, Department o, et al. Allopurinol in refractory epilepsy: A double blind study. Tehran University Medical Journal 2008;65(11):23-7.

55. Tousoulis D, Andreou I, Tentolouris C, Antoniades C, Papageorgiou N, Gounari P, et al. Comparative effects of rosuvastatin and allopurinol on circulating levels of matrix metalloproteinases and tissue inhibitors of metalloproteinases in patients with chronic heart failure. International Journal of Cardiology 2010;145(3):438-43.

56. Vogler WR, Drane JW. Effect of allopurinol on urinary ammonia excretion in patients with gout. Metabolism 1969;18(6):519-44.

57. Weiser M, Burshtein S, Gershon AA, Marian G, Vlad N, Grecu IG, et al. Allopurinol for mania: a randomized trial of allopurinol versus placebo as add-on treatment to mood stabilizers and/or antipsychotic agents in manic patients with bipolar disorder. Bipolar Disorders 2014;16(4):441-7.

58. Wells AF, MacDonald PA, Chefo S, Jackson RL. African American patients with gout: efficacy and safety of febuxostat vs allopurinol. Bmc Musculoskeletal Disorders 2012;13.

59. Whelton A, MacDonald PA, Hunt B, Gunawardhana L, Author A, Johns H, et al. The impact on renal function of quantitative serum urate reduction in gout patients. Arthritis and Rheumatism 2011;63:10 SUPPL. 1.

60. Yiginer O, Ozcelik F, Inanc T, Aparci M, Ozmen N, Cingozbay BY, et al. Allopurinol improves endothelial function and reduces oxidant-inflammatory enzyme of myeloperoxidase in metabolic syndrome. Clinical Research in Cardiology 2008;97(5):334-40.

61. Yiginer O, Ozcelik F, Inanc T, Aparci M, Ozmen N, Cingozbay BY, et al. Allopurinol improves endothelial function and reduces oxidant-inflammatory enzyme of myeloperoxidase in metabolic syndrome. Clinical Research in Cardiology 2008;97(5):334-40.

2) Second systematic literature search, from Sep 29, 2014 to January 11, 2016 (subtitles represent the reasons for exclusion).

Abstracts or full-text articles with no extractable data

1. Alem M, Alshehri A, Cahusac P, Walters M. Effect of xanthine oxidase inhibition on arterial stiffness in patients with chronic heart failure. Journal of hypertension; 2015:e47.

2. Babes EE, Babes VV, Popescu MI, Author A, Faculty of Medicine OR, Correspondence A, et al. Allopurinol and endothelial function. European Heart Journal 2014;35 SUPPL. 1:1125.

3. Separham A, Ghaffari S, Nafafi H, Ghojazadeh M, Author A, Cardiovascular Research Center T, et al. Allopurinol in patients with acute St elevation myocardial infarction undergoing thrombolytic therapy : A randomized trial. Cardiology 2015;(2015) 132 SUPPL. 1:205.

4. Simone VV, Cicoira M, Rossi A, Bergamini C, Zanolla L, Battaia E, et al. Xanthine oxidase inhibition in chronic heart failure: Effects on diastolic function and NT-proBNP levels in a double-blind, randomised, placebo-controlled study. European journal of heart failure; 2015:340.

5. Tani S, Nagao K, Hirayama A. Febuxostat, xanthine oxidase inhibitor, may improve cardiorenal interaction: a randomized controlled pilot study. European Heart Journal 2014;35:904-5.

Results could not be translated

1. Pour-Pouneh M, Narimani R, Mardani S, Momeni A, Nasri H, Author A, et al. Evaluation of the relationship between the reduction of serum uric acid level and control of blood pressure in patients with hypertension and hyperuricemia. Journal of Isfahan Medical School 2015;33(353):1672-85.

Follow-up or treatment duration <28 days

1. Sanchis-Gomar F, Salvagno GL, Lippi G. Inhibition of xanthine oxidase and exercise on serum uric acid, 25(OH)D3, and calcium concentrations. Clinical laboratory; 2014:1409-11.

2. Sanchis-Gomar F, Pareja-Galeano H, Gomez-Cabrera MC, Candel J, Lippi G, Salvagno GL, et al. Allopurinol prevents cardiac and skeletal muscle damage in professional soccer players. Scand J Med Sci Sports 2015;25(1):e110-5.

Different publications of studies already evaluated

1. Liu P, Chen Y, Wang B, Zhang F, Wang D, Wang Y. Allopurinol treatment improves renal function in patients with type 2 diabetes and asymptomatic hyperuricemia: 3-year randomized parallel-controlled study. Clinical endocrinology; 2015:475-82.

3) Third systematic literature search from Jan 11, 2016 to December 30, 2016 and hand searching process updated to December 30, 2016 (subtitles represent the reasons for exclusion).

OBS: Some of the articles presented in this section were already found in previous searches. The hand searching process was purposely redundant in order in increase sensitivity for eligible articles.

No placebo or no-treatment group

1. NCT01001338- Ardea Biosciences Incorporation. Lesinurad in combination with allopurinol: results of a phase 2, randomised, double-blind study in patients with gout with an inadequate response to allopurinol. In: ClinicalTrials.gov[Internet]. Bethesda (MD): National Library of Medicine (US). 2000- [cited 2016 Dec 27] . Available from: https://clinicaltrials.gov/ct2/show/NCT01001338.

2. Hosoya T, Ogawa Y, Hashimoto H, Ohashi T, Sakamoto R. Comparison of topiroxostat and allopurinol in Japanese hyperuricemic patients with or without gout: a phase 3, multicentre, randomized, double-blind, double-dummy,active-controlled, parallel-group study. J Clin Pharm Ther 2016;41:290-7.

3. Peixoto MR, Monego ET, Jardim PC, Carvalho MM, Sousa AL, Oliveira JS, et al. Diet and medication in the treatment of hyperuricemia in hypertensive patients. Arq Bras Cardiol 2001;76:463-72.

4. Viglioglia PA, Plante GE, Viglioglia J, Saraceno EF. Allopurinol in psoriasis. Dermatologica 1970;141:203-7.

5. Chunyan W, Lixun Z. The cloth division him for treatment of gout with high uric acid hematic disease analysis. Health Way 2016:15:15. (article in chinese)

6. Singh G, Kumar H, Soni U, Tyagi S, Aggarwal K, Verma K, et al. "COMPARISON OF URATE-LOWERING EFFICACY AND SAFETY OF FEBUXOSTAT AND ALLOPURINOL IN GOUT PATIENTS". Heterocyclic Letters 2016;6(1):133-47.

Intervention group received XOI or uricosuric at investigator’s discretion

1. Tan Y, Fu JZ, Liang M, Lin ZX, Huang J. Clinical observation of the effect of allopurinol to protect renal function in patients with diabetic nephropathy. Modern Hospital 2011;11:36–8. (article in chinese)

Review, editorial, observational or quasi-experimental studies

1. Smith MJ. Placebo versus allopurinol for recurrent urinary calculi. Proc Eur Dial Transplant Assoc 1983;20:422-6.

2. Smith MJ. Placebo versus allopurinol for renal calculi. J Urol 1977;117:690-2.

3. Smith MJ. Allopurinol and calcium-stone formers. Lancet 1973;1:315.

4. Zöllner N, Schattenkirchner M. Allopurinol in the treatment of gout and uric acid nephrolithiasis. Dtsch Med Wochenschr 1967;92:654-60.

5. Rundles RW, Metz EN, Silberman HR. Allopurinol in the treatment of gout. Ann Intern Med. 1966;64:229-58.

6. Bai W, Zhou W. Clinical research on diagnosis and treatment of patients with renal function damage induced by chronic uric acid nephropathy. China Medicine and Pharmacy 2014;7:214-216. http://caod.oriprobe.com/journals/zgyykx/%e4%b8%ad%e5%9b%bd%e5%8c%bb%e8%8d%af%e7%a7%91%e5%ad%a6.htm (article in chinese)

7. Satirapoj B, Wirajit O, Burata A, Supasyndh O, Ruangkanchanasetr P. Benefits of allopurinol treatment on blood pressure and renal function in patients with early stage of chronic kidney disease. Journal of the Medical Association of Thailand 2015;98(12):1155-61.

8. Steubl D, Michel MC. Experimental and clinical nephroprotection by the xanthine oxidase inhibitor febuxostat. Naunyn-Schmiedeberg's Archives of Pharmacology 2016;389(8):815-7.

9. Filiopoulos V, Hadjiyannakos D, Vlassopoulos D. Febuxostat renoprotection in CKD patients with asymptomatic hyperuricemia. American Journal of Kidney Diseases 2016;67(6):989-90.

Study protocol

1. Oyama J, Tanaka A, Sato Y, Tomiyama H, Sata M, Ishizu T, et al. Rationale and design of a multicenter randomized study for evaluating vascular function under uric acid control using the xanthine oxidase inhibitor, febuxostat: the PRIZE study. Cardiovasc Diabetol 2016;15:87.

2. Saddekni MB, Saag KG, Dudenbostel T, Oparil S, Calhoun DA, Sattui SE, et al. The effects of urate lowering therapy on inflammation, endothelial function, and blood pressure (SURPHER) study design and rationale. Contemp Clin Trials 2016;50:238-44.

3. Mackenzie IS, Ford I, Walker A, Hawkey C, Begg A, Avery A, et al. Multicentre, prospective, randomised, open-label, blinded end point trial of the efficacy of allopurinol therapy in improving cardiovascular outcomes in patients with ischaemic heart disease: protocol of the ALL-HEART study. BMJ Open 2016;6(9):e013774.

Inclusion of individuals <18 years-old

1. Apt W, Aguilera X, Arribada A, Pérez C, Miranda C, Sánchez G,et al. Treatment of chronic Chagas' disease with itraconazole and allopurinol. Am J Trop Med Hyg 1998;59:133-8.

2. Llanos-Cuentas A, Echevarría J, Cruz M, La Rosa A, Campos P, Campos M, et al. Efficacy of sodium stibogluconate alone and in combination with allopurinol for treatment of mucocutaneous leishmaniasis. Clin Infect Dis 1997;25:677-84.

3. Velez I, Agudelo S, Hendrickx E, Puerta J, Grogl M, Modabber F, et al. Inefficacy of allopurinol as monotherapy for Colombian cutaneous leishmaniasis. A randomized, controlled trial. Ann Intern Med 1997;126:232-6.

4. Horsfall PA, Plummer J, Allan WG, Girling DJ, Nunn AJ, Fox W. Double blind controlled comparison of aspirin, allopurinol and placebo in the management of arthralgia during pyrazinamide administration. Tubercle 1979;60:13-24.

Different publications of studies already evaluated

1. NCT02063997 - CymaBay Therapeutics Incorporation. Evaluate the Efficacy and Safety of Arhalofenate for Preventing Flares and Reducing Serum Uric Acid in Gout PatientsIn: ClinicalTrials.gov[Internet]. Bethesda (MD): National Library of Medicine (US). 2000- [cited 2016 Dec 27] . Available from: https://clinicaltrials.gov/ct2/show/NCT02063997.

2. NCT01228903 – University of Colorado, Denver. Uric Acid and the Endothelium in CKD. In: ClinicalTrials.gov[Internet]. Bethesda (MD): National Library of Medicine (US). 2000- [cited 2016 Dec 27]. Available from:http://www.clinicaltrial.co/ShowTrial/NCT01228903.

3. NCT02327754 – Sanwa Kagaku Kenkyusho Company. Effect of Topiroxostat on Urinary Albumin Excretion Early Stage Diabetic Nephropathy and Hyperuricemia With or Without Gout. In: ClinicalTrials.gov[Internet]. Bethesda (MD): National Library of Medicine (US). 200- [cited 2016 Dec 27] . Available from: https://clinicaltrials.gov/ct2/show/NCT02327754.

4 Dalbeth N, Saag K, Palmer W, et al. Multicenter, Randomized, Double-Blind, Phase 2 Study to Evaluate the Effect of Febuxostat Versus Placebo on Joint Damage in Hyperuricemic Subjects with Early Gout. Annals of the Rheumatic Diseases 2015;74:543.

5. Steinberg A, Chera H, Choi YJ, Martin R, McWherter C, Zhang Y, Boudes P. A Study to Evaluate the Efficacy and Safety of Arhalofenate for Preventing Flares and Reducing Serum Uric Acid in Gout Patients [abstract]. Arthritis Rheumatol. 2015; 67 (suppl 10). http://acrabstracts.org/abstract/a-study-to-evaluate-the-efficacy-and-safety-of-arhalofenate-for-preventing-flares-and-reducing-serum-uric-acid-in-gout-patients/

6. Dalbeth N, Saag KG, Palmer W, Choi H, Hunt B, MacDonald P, Thienel U, Gunawardhana L. Imaging and Safety Assessments Following Treatment with Febuxostat and Placebo for 2 Years in Subjects with Early Gout [abstract]. Arthritis Rheumatol. 2015; 67 (suppl 10). http://acrabstracts.org/abstract/imaging-and-safety-assessments-following-treatment-with-febuxostat-and-placebo-for-2-years-in-subjects-with-early-gout/.

7. Hill E, Higgs JBm Sky K, Sit M, Collamer NA, Author A, et al. Does strarting allopurinol porlong acute treated gout: Artthritis Rheum 2013,65(Suppl 10):S730

8. Gunawardhana L, Becker MA, Whelton A, Hunt B, Castillo M, Dong X, Saag K. A Phase 2 Study to Evaluate the Efficacy and Safety of Febuxostat Extended- Versus Immediate-Release Formulations in Patients with Gout and Moderate Renal Impairment [abstract]. Arthritis Rheumatol. 2016; 68 (suppl 10). http://acrabstracts.org/abstract/a-

phase-2-study-to-evaluate-the-efficacy-and-safety-of-febuxostat-extended-versus-immediate-release-formulations-in-patients-with-gout-and-moderate-renal-impairment

9. Saag K, Becker MA, Whelton A, Hunt B, Castillo M, Kisfalvi K, Gunawardhana L. A Phase 3 Study to Evaluate the Efficacy and Safety of Febuxostat Extended- Versus Immediate-Release Formulations in Patients with Gout [abstract]. Arthritis Rheumatol. 2016; 68 (suppl 10). http://acrabstracts.org/abstract/a-phase-3-study-to-evaluate-the-efficacy-and-safety-of-febuxostat-extended-versus-immediate-release-formulations-in-patients-with-gout

10. Dalbeth N, Saag KG, Palmer W, Choi HK, Hunt B, MacDonald P, Thienel U, Gunawardhana L. Overall Reduction in Acute Flares during Treatment with Febuxostat Compared with Placebo over 2 Years in Patients with Early Gout [abstract]. Arthritis Rheumatol. 2016; 68 (suppl 10). http://acrabstracts.org/abstract/overall-reduction-in-acute-flares-during-treatment-with-febuxostat-compared-with-placebo-over-2-years-in-patients-with-early-gout/.

11. Hara S, Ohno I, Nomura S, Hisatome I, Uchida S, Fujimori S, et al. Effects of topiroxostat on the serum urate levels and urinary albumin excretion in hyperuricemic stage 3 ckd patients with or without gout. Nephrology Dialysis Transplantation 2015;30:iii159.

12. NCT 01988402 –Wilford Hall Medical Center. Does Allopurinol prolong a treated, acute gout flare? In: ClinicalTrials.gov[Internet]. Bethesda (MD): National Library of Medicine (US). 2000- [cited 2016 Dec 27]. Available from: https://clinicaltrials.gov/ct2/show/NCT01988402

13. NCT 00512057 –University of Dundee. APEX Study: Effects of Allopurinol on Coronary and Peripheral Endothelial Function in Patients With Cardiac Syndrome X.In: ClinicalTrials.gov[Internet]. Bethesda (MD): National Library of Medicine (US). 2000- [cited 2016 Dec 27]. Available from: https://clinicaltrials.gov/ct2/show/NCT00512057?id=NCT00512057&rank=1&load=cart

14. NCT00997542 –National Heart and Lung Institute. A Double-blind, Placebo-controlled, Cross-over Study of the Effects of Allopurinol on Oxidative Metabolism, Peripheral Blood Flow and Immune Function in Patients With Advanced Chronic Heart Failure (CHF).In: ClinicalTrials.gov[Internet]. Bethesda (MD): National Library of Medicine (US). 2000- [cited 2016 Dec 27]. Available from: https://clinicaltrials.gov/ct2/show/NCT00997542

15. NCT01157936 –Instituto Nacional de Cardiologia Ignacio Chavez. Effect of Hyperuricemia Treatment on Hypertension and Metabolic Syndrome. In: ClinicalTrials.gov[Internet]. Bethesda (MD): National Library of Medicine (US). 2000- [cited 2016 Dec 27]. Available from: https://clinicaltrials.gov/ct2/show/NCT01157936

16. NCT00821392 - SK Chemicals Co.,Ltd. A Randomized, Multi-Center, Double Blinded, Allopurinol-Controlled, Placebo-Controlled Parallel Group, 5-Arm, Dose-Response,Bridging Study to Assess the Efficacy and Safety of Febuxostat in Subject With Gout. In: ClinicalTrials.gov[Internet]. Bethesda (MD): National Library of Medicine (US). 2000- [cited 2016 Dec 27]. Available from: https://clinicaltrials.gov/ct2/show/NCT00821392

17. NCT02038179 - University of Alabama at Birmingham. UAB Center of Research Translation (CORT) Project 2: The Effects of Urate Lowing Therapy (ULT) in Inflammation, Endothelial Function, and Blood Pressure. In: ClinicalTrials.gov[Internet]. Bethesda (MD): National Library of Medicine (US). 2000- [cited 2016 Dec 27]. Available from: https://clinicaltrials.gov/ct2/show/NCT02038179

18. NCT01082640 – Takeda. A Multicenter, Randomized, Double-Blind, Phase 2 Study to Evaluate the Effect of Febuxostat Versus Placebo on Renal Function in Gout Subjects With Hyperuricemia and Moderate to Severe Renal Impairment. . In: ClinicalTrials.gov[Internet]. Bethesda (MD): National Library of Medicine (US). 2000- [cited 2016 Dec 27]. Available from: https://clinicaltrials.gov/ct2/show/NCT01082640

Study duration less than 28 days

1. Von Haeling S, Bode-Böger SM, Martens-Lobenhoffer J, Rauchhaus M, Schefold JC, Genth-Zotz S, et al. Elevated levels of asymmetric dimethylarginine in chronic heart failure: a pathophysiologic link between oxygen radical load and impaired vasodilator capacity and the therapeutic effect of allopurinol. Clin Pharmacol Ther 2010;88:506-12.

2. Martinez S, Gonzalez M, Vernaza ME. Treatment of cutaneous leishmaniasis with allopurinol and stibogluconate. Clin Infect Dis 1997;24:165-9.

3. Ababei M, Campeanu A, Nistorescu D, Zaharia O, Gheorghe G, Nanea T. The effect of Allopurinol on endothelial function, serum uric acid and NTproBNP in acute decompensated heart failure. European Heart Journal 2016;37:204.

Duration of follow-up different from period on medication

1. Xiao J, Deng SB, She Q, Li J, Kao GY, Wang JS, Ma Y. Allopurinol ameliorates cardiac function in non-hyperuricaemic patients with chronic heart failure. Eur

Rev Med Pharmacol Sci. 2016;20(4):756-61.

2. Goicoechea M, Garcia de Vinuesa S, Verdalles U, Verde E, Macias N, Santos A, Pérez de Jose A, Cedeño S, Linares T, Luño J. Allopurinol and progression of CKD and cardiovascular events: long-term follow-up of a randomized clinical trial. Am J Kidney Dis 2015;65:543-9.

Ongoing studies or complete results not available

1. NCT02078219 - AstraZeneca. A Randomized, Double-Blind, Multicenter, Placebo-Controlled, Parallel Group, 24-Week Phase II Study to Evaluate Efficacy and Safety of RDEA3170 5 mg, 7.5 mg, 10 mg, 12.5 mg and 15 mg Versus Placebo and Open-Label Allopurinol 200 mg as a Reference Arm in Japanese Patients With Gout or Asymptomatic Hyperuricemia. In: ClinicalTrials.gov[Internet]. Bethesda (MD): National Library of Medicine (US). 2000- [cited 2016 Dec 27]. Available from: https://clinicaltrials.gov/ct2/show/NCT02078219

2. NCT00477789 - Universita di Verona. Effects of Allopurinol on Diastolic Function in Chronic Heart Failure Patients. In: ClinicalTrials.gov[Internet]. Bethesda (MD): National Library of Medicine (US). 2000- [cited 2016 Dec 27]. Available from: https://clinicaltrials.gov/ct2/show/NCT00477789

3. NCT01332799 - University of Iowa. Xanthine Oxidase Inhibition in Renal Transplant Recipients. In: ClinicalTrials.gov[Internet]. Bethesda (MD): National Library of Medicine (US). 2000- [cited 2016 Dec 27]. Available from: https://clinicaltrials.gov/ct2/show/NCT01332799

4. NCT01320722 - Brigham and Women's Hospital. Modifiable Effectors of Renin System Activation: Treatment Evaluation (MODERATE). In: ClinicalTrials.gov[Internet]. Bethesda (MD): National Library of Medicine (US). 2000- [cited 2016 Dec 27]. Available from: https://clinicaltrials.gov/ct2/show/NCT01320722

5. NCT01984749 - Freed Study Group. A Multicenter, Randomized, Comparative Trial on the Effect of Febuxostat in Preventing Cerebral and Cardiorenovascular Events in Patients With Hyperuricemia. . In: ClinicalTrials.gov[Internet]. Bethesda (MD): National Library of Medicine (US). 2000- [cited 2016 Dec 27]. Available from: https://clinicaltrials.gov/ct2/show/NCT01984749

6. NCT01472692 - University of California, San Diego. reatment of Uric Acid With the Xanthine Oxidase Inhibitor Febuxostat: Effects on Blood Pressure, Metabolic Markers, and

Aortic Stiffness in Prehypertensive. In: ClinicalTrials.gov[Internet]. Bethesda (MD): National Library of Medicine (US). 2000- [cited 2016 Dec 27]. Available from: https://clinicaltrials.gov/ct2/show/NCT01472692

7. NCT02866214 - Ain Shams University. Evaluation of the Effect of Febuxostat on Endothelial Dysfunction in Hemodialysis Patients. In: ClinicalTrials.gov[Internet]. Bethesda (MD): National Library of Medicine (US). 2000- [cited 2016 Dec 27]. Available from: https://clinicaltrials.gov/ct2/show/NCT02866214

8. NCT01328769 - Paul N. Hopkins. Febuxostat, Blood Pressure and the Intrarenal Renin-Angiotensin System (RAS). In: ClinicalTrials.gov[Internet]. Bethesda (MD): National Library of Medicine (US). 2000- [cited 2016 Dec 27]. Available from: https://clinicaltrials.gov/ct2/show/NCT01328769

9. NCT02017171 - Alessandro Doria. PERL: A Multicenter Clinical Trial of Allopurinol to Prevent GFR Loss in T1D. In: ClinicalTrials.gov[Internet]. Bethesda (MD): National Library of Medicine (US). 2000- [cited 2016 Dec 27]. Available from: https://clinicaltrials.gov/ct2/show/NCT02017171

10. Johnson D. A randomised Controlled trial of slowing of Kidney Disease progression From the Inhibition of Xanthine oxidase (CKD-FIX). In: aktn.org.au [Internet]. Australasian Kidney Trials Network (AU). - [cited 2016 Dec 27]. Available from: https://aktn.org.au/ckd-fix/

Abstracts or full-text articles with no extractable data

1. Li J, Zhang Y, Xu Y, Wang J, Xiao J. Allopurinol for Ameliorating Exercise Tolerance of Non - Hyperuricemic Patients with Chronic Heart Failure in 62 Cases. China Pharmaceuticals 2015;54:2129-2137. (article in chinese)

2. Zheng-Xiang T, Quan-qing L, Li Z. Clinical research on allopurinol lowering of uric acid level of chronic kidney disease patient. Journal of Hunan Normal University (Medical Science) 2015;12:54-56. (article in chinese)

3. Zhou L, Ren J, Li J. Effect of controlled-release nifedipine plus allopurinol on pulse pressure and atherosclerosis in patients with hypertension and high uric acid. : China Modern Doctor 2012;19:66-68. (article in chinese)

4. Yin G, Xia QL, Li YF. Curative effect of allopurinol in patients with acute myocardial infarction complicating hyperuricemia. China Journal Evidence Based Cardiovascular Medicine 2014;6:436-437. (article in chinese)

5. Junxia Z, Cheng C, Bilin Z, Yangyang C. Effect of allopurinol on the endothelial function of patients with impaired fasting glucose complicated with hyperuricemia. Chinese Journal of Diabetes Mellitus 2016;10:618-622. (article in chinese)

6. Yan CQ, Liu XY, Wang J, Chen T, Xiao X. Effect of allopurinol on heart function in chronic congestive heart failure. Journal of Jinggangshan University(Natural Science) 2009;2:81-82. (article in chinese)

7. Luo LM, Chen QK, Yang XJ. Influence of acid-lowering therapy on IL-1 Band NALP3 levels in gout patients with hyperuricemia. Acta Academiae Medicinae Jiangxi 2016;56:63-66. (article in chinese)

8. Miao X, Xu X. The research on the relationship between uric acid and type 2 diabetic nephropathy. Jilin Medical Journal 2014;22:4862-4864. (article in chinese)

9. Yang H, Zeng Y, Liao Q. Effects of allopurinol on vascular endothelial function in patients with chronic heart failure. Journal of Postgraduates of Medicine 2005;28:32-34. (article in chinese)

10. Zhou X, Yin Z. Clinical observation of febuxostat in the treatment of chronic kidney disease with hyperuricemia. Acta Academiae Medicinae Xuzhou 2015; 12:870-872. (article in chinese)

11. Leng L, Ma Y, Ma Y, Zheng H, Feng Y. The cardiac function effect of allopurinol for hyperuricemia combined with dilated cardiomyopathy patients. Chinese Journal of Postgraduates of Medicine 2014;14:48-51. (article in chinese)

12. Sui L, Yang R, Du J, Yang B, Yang M. Efficacy of Febuxostat in surgical treatment of tophus. China Journal of Modern Medicine 2015. 29:73-77. (article in chinese)

13. Xiao H, Chen G, Lin K, Nin G. Effect of allopurinol on exercise in patients with chronic stable angina. Chinese Journal of Thrombosis and Hemostasis 2014;3:117-120. (article in chinese)

14. Guo L, Liu L, Li T, Ma Y, Zhu M, Zhang S. Study of the Allopurinol on Atrial Fibrillation of the Patients with Heart Failure Complicated with Hyperuricemia. Medical Information 2016;24:109-10. (article in chinese)

15. Akiyama D, Furuya F, Wakasugi M, Kitamura K. Efficacy offebuxostat in type 2 diabetic patients with kidney impairment. Nephrology dialysis transplantation. Conference: 53rd ERA-EDTA congress. Vienna austria. Conference start: 20160521. Conference end: 20160524. Conference publication: (var.pagings); 2016:i202-i3.

16. Nagatomo Y, Funamizu T, Izumi Y, Yagawa M, Mahara K, Hen Y, et al. Effects of febuxostat on cardiorenal function in patients with heart failure and hyperuricemia: effect study. European journal of heart failure. Conference: heart failure 2016 and the 3rd world congress on acute heart failure. Florence italy. Conference start: 20160521. Conference end: 20160524. Conference publication: (var.pagings); 2016:191-2.

17. Omrani H, Sadeghi SMK, Raeisi D, Hashemian A. The effect of allopurinol treatment regimen on serum uric acid and arterial blood pressure in hemodialysis patients. Journal of kermanshah university of medical sciences; 2016:56-61.

18. Pichholiya M, Yadav AK, Luhadia SK, Tahashildar J, Aseri ML. A comparative study of efficacy and safety of febuxostat and allopurinol in pyrazinamide-induced hyperuricemic tubercular patients. Indian Journal of Pharmacology 2016;48(5):522-5.

19. NCT01763996- Takeda. The Influence of Febuxostat on Coronary Artery Endothelial Dysfunction in Subjects With Chronic Stable Angina: A Phase 4 Randomized, Placebo-Controlled, Double-Blind, Cross-Over Study. In: ClinicalTrials.gov[Internet]. Bethesda (MD): National Library of Medicine (US). 2000- [cited 2016 Dec 27]. Available from: https://clinicaltrials.gov/ct2/show/study/NCT01763996

Publications not obtained

1. Einaudi G, Marrazzi G, Pecchiura G. Observations on a random group of gout patients. Reumatismo 1972;24:21-9.

2. Muszkowska-Penson J, Chodorowski Z. Allopurinol in the treatment of gout. Pol Tyg Lek 1969;24:286-8.

3. Nyfos. Treatment of the gouty diathesis with a xanthine oxidase inhibitor. A clinical trial of allopurinol. Ugeskr Laeger 1967;129:253-7.

Results could not be translated

1. Eyman E, Brzozowska-Jurkowska A, Weryńska-Przybylska J. Clinical evaluation of Milurit. Reumatologia. 1973;11:299-304.

Already found in systematic literature search

1. Madero M, Rodríguez Castellanos FE, Jalal D, Villalobos-Martín M, Salazar J, Vazquez-Rangel A, et al. A pilot study on the impact of a low fructose diet and allopurinol on clinic blood pressure among overweight and prehypertensive subjects: a randomized placebo controlled trial. J Am Soc Hypertens 2015;9:837-44.

2. Robertson AJ, Struthers AD. A Randomized Controlled trial of Allopurinol in Patients With Peripheral Arterial Disease. Can J Cardiol 2016;32:190-6.

3. Kamatani N, Fujimori S, Hada T, Hosoya T, Kohri K, Nakamura T, et al. Placebo-controlled double-blind dose-response study of the non-purine-selective xanthine oxidase inhibitor febuxostat (TMX-67) in patients with hyperuricemia (including gout patients) in japan: late phase 2 clinical study. J Clin Rheumatol 2011;17:S35-43.

4. Tousoulis D, Andreou I, Tsiatas M, Miliou A, Tentolouris C, Siasos G, et al . Effects of rosuvastatin and allopurinol on circulating endothelial progenitor cells in patients with congestive heart failure: the impact of inflammatory process and oxidative stress. Atherosclerosis 2011;21:151-7.

5. George J, Carr E, Davies J, Belch JJ, Struthers A. High-dose allopurinol improves endothelial function by profoundly reducing vascular oxidative stress and not by lowering uric acid. Circulation 2006;114:2508-16.

6. Merimsky O, Inbar M, Chaitchik S. Treatment of advanced colorectal cancer by 5-fluorouracil-leucovorin combination with or without allopurinol: a prospective randomized study. Anticancer Drugs 1991;2:447-51.

7. Gibson T, Simmonds HA, Potter C, Rogers V. A controlled study of the effect of long term allopurinol treatment on renal function in gout. Adv Exp Med Biol 1980;122A:257-62.

8. Liu P, Wang H, Zhang F, Chen Y, Wang D, Wang Y. The Effects of Allopurinol on the Carotid Intima-media Thickness in Patients with Type 2 Diabetes and Asymptomatic Hyperuricemia: A Three-year Randomized Parallel-controlled Study. Intern Med 2015;54:2129-37.

9. Ivanov DD, Ivanova MD. Febuxostat improves GFR and BP in non-diabetic adults with CKD 2-3: 4 years follow-up. Nephrology Dialysis Transplantation 2015;30:iii486-iii7.

Fraudulent studies

1. Salim AS. Oxygen-derived free radicals and the prevention of duodenal ulcer relapse: a new approach. Am J Med Sci 1990;300:1-6; discussion 7-8.

2. Salim AS. The relationship between Helicobacter pylori and oxygen-derived free radicals in the mechanism of duodenal ulceration. Intern Med 1993;32:359-64.

3. Salim AS. Role of free radical scavengers in the management of refractory duodenal ulceration. A new approach. J Surg Res 1994;56:45-52.

4. Salim AS. Scavengers of oxygen-derived free radicals prolong survival in advanced colonic cancer. A new approach. Tumour Biol 1993;14:9-17.

5. Salim AS. Allopurinol and dimethyl sulfoxide improve treatment outcomes in smokers with peptic ulcer disease. J Lab Clin Med 1992;119:702-9.

6. Salim AS. Oxygen-derived free-radical scavengers prolong survival in gastric cancer. Chemotherapy 1992;38:135-44.

7. Salim AS. Oxygen-derived free-radical scavengers prolong survival in colonic cancer. Chemotherapy. 1992;38:127-34.

8. Salim AS. The role of oxygen-derived free radicals in the management of venous (varicose) ulceration: a new approach. World J Surg 1991;15:264-9.
