# Supplementary material for: Xanthine oxidase inhibitors for prevention of cardiovascular events: a systematic review and meta-analysis of randomized controlled trials
Source: BMC Cardiovasc Disord. 2018 Feb 7;18:24. doi: 10.1186/s12872-018-0757-9 (PMC5804046; doi:10.1186/s12872-018-0757-9)
Supplement: Supplementary file 4 — Study protocol. Study protocol registered in PROSPERO. (PDF 100 kb) [file 12872_2018_757_MOESM4_ESM.pdf]

Xanthine oxidase inhibitors for primary and secondary prevention of cardiovascular events

Markus Bredemeier, Guilherme Campos, Matheus Eisenreich, Fernando Stein, André Morsch

Citation

Markus Bredemeier, Guilherme Campos, Matheus Eisenreich, Fernando Stein, André Morsch. Xanthine oxidase inhibitors for primary and secondary prevention of cardiovascular events. PROSPERO 2015 CRD42015016073 Available from: http://www.crd.york.ac.uk/PROSPERO/display_record.php?ID=CRD42015016073

Review question

Do xanthine oxidase Inhibitors (XOI) exert a preventive effect (primary or secondary) on the incidence of major cardiovascular events and mortality?
What is the incidence of serious and non-serious adverse events associated with the use of XOI?
Is there an association between the dose of XOI and its possible effect on cardiovascular outcomes?
Are there specific subsets of patients who could benefit more from the use of XOI (individuals with and without cardiovascular risk factors; patients with and without established cardiovascular disease)?
Do purine-like and non-purine XOI exert the same protective effect for cardiovascular events?

Searches

Major electronic databases (PubMed, EMBASE, Web of Science, Cochrane Library, and Lilacs) were searched for published literature from their inception to September 29, 2014. Hand search performed scrutinizing the reference lists of the identified trials and review articles; the ClinicalTrials.gov, Clinicaltrialsregister.eu, and Google web sites. Missing or unpublished data will be actively sought by contacting authors via e-mail. No language restriction was applied. The literature search will be uptaded if necessary.

Types of study to be included

Inclusion criteria: randomized controlled trials with treatment duration longer than or equal to 28 days.
Exclusion criteria: inclusion of individuals less than 18 years old.

Condition or domain being studied

Major cardiovascular events and mortality.

Participants/population

Individuals aged 18 years or more participating in randomized controlled trials.

Intervention(s), exposure(s)

Treatment with purine-like XOI (allopurinol, oxypurinol, tisopurine) or non-purine XOI (febuxostat, topiroxostat).

Comparator(s)/control

Placebo or no treatment.

Primary outcome(s)

Major cardiovascular events: cardiovascular death, non-fatal myocardial infarction, unstable angina requiring urgent revascularization, and non-fatal stroke.
Death from any cause.

Secondary outcome(s)

Total thromboembolic events (TE), arterial TE (cardiac events: unstable angina and myocardial infarction; peripheral and visceral events: thrombosis or emboli causing critical ischemia; cerebral events: ischemic

stroke or transient ischemic attacks) and venous TE (deep venous thrombosis; thrombosis in central, visceral or cerebral veins; and pulmonary embolism).
Individual outcomes defined as major cardiovascular events and thromboembolic events, heart failure or worsening heart failure, serious ventricular arrhythmias (ventricular tachycardia, ventricular fibrillation), total cardiac arrhythmias, coronary heart disease (CHD, including episodes of angina), hypertension or worsening hypertension, and total adverse cardiovascular events (any of the above).
Total adverse events, skin rash, serious hypersensitivity reactions, serious adverse events (those requiring urgent medical procedures and/or hospitalization, life-threatening or leading to death), worsening renal function (including necessity of renal replacement therapy).
Withdrawal before study ending, early withdrawal due to adverse events.

Data extraction (selection and coding)

The references obtained using the search strategy will be evaluated (based on title and abstract) by two independent investigators. The articles selected by at least one of the investigators will be obtained in electronic or printed full-text format and will be re-evaluated for inclusion by two independent investigators. The final decision for inclusion will be made by consensus or discussion with a third observer in case of divergence. Multiple reports from a single study will be considered one study.
Data on cardiovascular events, adverse events, and withdrawal from study will be independently extracted by two researchers using a specifically designed protocol. Divergences were resolved by discussion with a third observer. In the case of cross-over trials, only the data of the first study period (before cross-over of patients) will be used.

Risk of bias (quality) assessment

The evaluation of risk of bias will be made by two independent assessors using the method described in the Cochrane Handbook (Higgins JPT, Green S [editors]. Cochrane Handbook for Systematic Reviews of Interventions Version 5.1.0 [updated March 2011]. The Cochrane Collaboration, 2011). Disagreements will be resolved by discussion envolving a third observer.

Strategy for data synthesis

Aggregate data extracted from RCTs will be analyzed using modified the intention-to-treat (considering patients who received at least one dose of the allocated treatment) or intention-to-treat results. If that is not possible, available case analysis will be employed.
The outcomes will be described in terms of odds-ratio (OR) and risk difference (RD), along with 95% confidence intervals. Considering the expected rarity of events, associations will be analyzed using the Peto OR or Mantel-Haenszel OR without zero-cell corrections and the Mantel-Haenszel methods for RD.
Heterogeneity will be evaluated using Cochran’s Q test and I-squared statistics and will be considered present when the Cochran’s test shows $P <= 0.10$ or the I-squared statistic is 40% or more. Random effects model will be used to account for heterogeneity; otherwise, the fixed-effects model will be used. P values less than or equal to 0.05 will be considered statistically significant. Sensitivity analyses will be conducted to account for a high risk of bias. Publication bias will be assessed using funnel plot analysis.
Meta-regression analyses are planned including independent variables as duration of treatment and follow-up, age, dosage of XOI, year of study beginning, percent of patients using anti-thrombotic agents and statins.

Analysis of subgroups or subsets

Separate analysis in patients with and without established vascular disease.
Among patients without established vascular disease, separate analysis of studies including patients with and without risk factors for cardiovascular disease.
Analysis in specific subset of patients: hypertension, diabetes, gout and hyperuricemia. renal failure, heart failure, CHD, use of statins, use of anti-thrombotic agents, male gender.

Contact details for further information

Dr Bredemeier
markbred@terra.com.br

Organisational affiliation of the review
Rheumatology Service at Hospital Nossa Senhora da Conceição - Grupo Hospitalar Conceição
www.ghc.com.br

Review team members and their organisational affiliations
Dr Markus Bredemeier. Rheumatology Service - Hospital Nossa Senhora da Conceição
Dr Guilherme Campos. Rheumatology Service at Hospital Nossa Senhora da Conceição
Dr Matheus Eisenreich. Rheumatology Service at Hospital Nossa Senhora da Conceição
Dr Fernando Stein. Internal Medicine Service at Hospital Nossa Senhora da Conceição
Dr André Morsch. Internal Medicine Service at Hospital Nossa Senhora da Conceição

Anticipated or actual start date
01 September 2014

Anticipated completion date
30 April 2015

Funding sources/sponsors
None

Conflicts of interest
None known

Language
English

Country
Brazil

Stage of review
Review_Ongoing

Subject index terms status
Subject indexing assigned by CRD

Subject index terms
Cardiovascular Diseases; Enzyme Inhibitors; Cardiovascular Diseases; Humans; Xanthine Oxidase

Date of registration in PROSPERO
22 January 2015

Date of publication of this version
22 January 2015

Details of any existing review of the same topic by the same authors

Stage of review at time of this submission

Stage	Started	Completed
Preliminary searches	Yes	Yes
Piloting of the study selection process	Yes	Yes
Formal screening of search results against eligibility criteria	Yes	No
Data extraction	No	No
Risk of bias (quality) assessment	No	No
Data analysis	No	No

Versions

22 January 2015

PROSPERO
This information has been provided by the named contact for this review. CRD has accepted this information in good faith and registered the review in PROSPERO. CRD bears no responsibility or liability for the content of this registration record, any associated files or external websites.
